# The organophosphorus synthesis triangle: introducing methods for the missing quaternization and de-quaternization routes

**DOI:** 10.1039/d5sc04496k

**Published:** 2025-10-24

**Authors:** Anna C. Vetter, Yannick Ortin, Kirill Nikitin, Declan G. Gilheany

**Affiliations:** a School of Chemistry, University College Dublin Belfield Dublin 4 Ireland kirill.nikitin@ucd.ie declan.gilheany@ucd.ie

## Abstract

In organophosphorus chemistry, several established reactions, such as the conversion of phosphorus trichloride into tertiary phosphines, followed by oxidation and quaternization to form phosphine oxides and phosphonium salts, are widely recognized and routinely applied. In contrast, other potentially valuable transformations, including reverse or complementary versions of these standard synthetic routes, remain largely unexplored or technically challenging. This work introduces two new reaction pathways that broaden the scope of organophosphorus synthesis. The first involves a P–C bond-forming process that enables interconversion of symmetrical phosphine oxides, such as triphenylphosphine oxide (Ph_3_PO), into P-stereogenic phosphine oxides and quaternary phosphonium salts. The second transformation is based on the distinctive reactivity of methoxymethyl (MOM)-substituted quaternary phosphonium salts. These compounds undergo a P–C bond cleavage reaction that results in de-quaternization, allowing the synthesis of mixed-substituent tertiary phosphines from triphenylphosphine as a common precursor. Together, these two processes provide multiple efficient synthetic routes to phosphines, phosphine oxides, and quaternary phosphonium salts. The overall synthetic approach is flexible, so that the target compounds can be obtained through several pathways using different substituent combinations as starting materials.

## Abstract 2

New reverse organophosphorus transformations –A and –B offer versatile synthetic approaches to all types of alkyl–aryl phosphines, their oxides and phosphonium salts from widely available Ph_3_P and Ph_3_PO.

## Graphical abstract

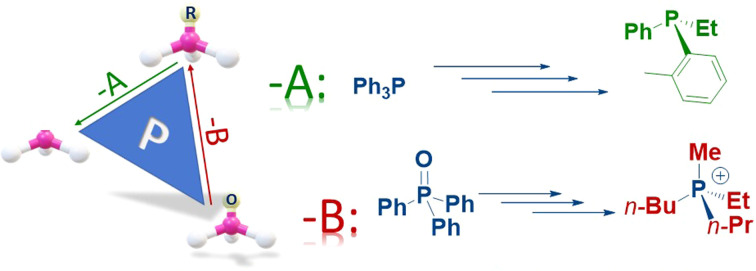

## Introduction

**Scheme 1:**
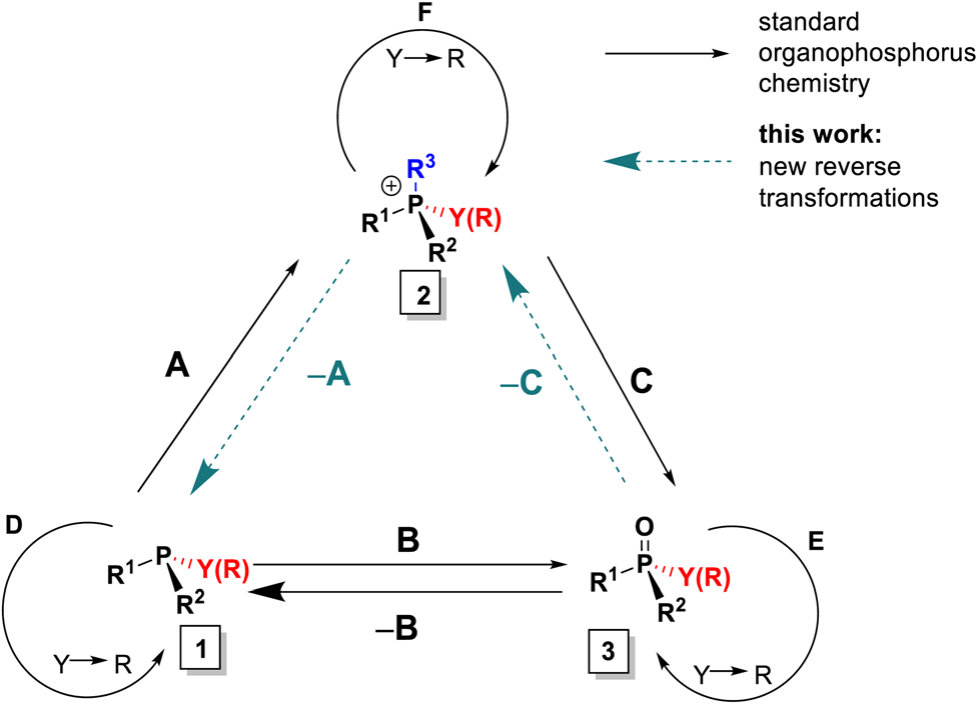
The “P-triangle” interconnects P(iii) and P(v) derivatives: 1 (*e.g.* phosphines, halophosphines), 2 (phosphonium salts) and 3 (*e.g.* phosphine oxides, phosphinates). Y – a leaving group; R – a C-linked organic group. Transformations between classes: A – quaternization; B – oxidation; C – olefination or hydrolysis.

In the context of the importance of phosphorus compounds in Nature, industrial materials and synthesis,^1^ the synthetic chemistry of organophosphorus compounds assumed a standing from the earliest days of organic synthesis^2^ and today is explored extensively.^3^ However, despite all of that activity, there are still a few basic interconversions that are difficult or impossible to achieve and some phosphorus substitution patterns that are inaccessible. To illustrate the issues, we introduce here the Organophosphorus Synthesis Triangle (or P-triangle, Scheme 1). This reflects that, historically, organophosphorus synthesis developed around a trio of key molecular motifs: P(iii) compounds 1 and their P(v) quaternized, 2, and oxidized derivatives, 3 (Scheme 1).^2*b*,3*d*,*f*^ Structure 1 is represented largely by tertiary phosphines R_3_P but also includes halophosphines and certain P–O structures: for example phosphonous acid derivatives (R = organic group, Y = leaving group, *e.g.* alkoxy, halide). Similarly, structure 3 describes both phosphine oxides (PO) and phosphon(in)ic acid derivatives. Structure 2 is mostly associated with quaternary phosphonium salts, (QPS, R = organic group) but also encompasses their oxo- and halo-analogues. The interconnectivity, both between 1–3 (transformations A–C) and within these three classes (replacing Y with an organic R, transformations D–F), has served many lines of synthetic chemistry in accessing, for example: important building blocks in organic synthesis,^4^ indispensable ligands^5^ for transition metal catalysis and, notably, chiral non-racemic variants of such ligands along with their organo- and phase transfer counterparts.^6^ Thus, adding new, and developing existing, efficient routes within the P-triangle is an important and topical task.

When we examine the P-triangle in more detail, it illustrates the close synthetic relationships of the key organophosphorus classes 1–3. Triorganophosphines 1, being often the entry point, are commonly, albeit not exclusively,^7^ accessed from chlorophosphines or, more recently, arylphosphines, by sequential introduction of organic groups R according to path D.^8^ From this point, established forward quaternization A^9*a*–*c*^ and oxidation B^9*d*^ processes allow relatively easy access to tetrahedral 2 and 3, respectively. In turn, facile cleavage of P–C bonds in 2, by, for example, hydrolysis or Wittig reaction^10^ path C, can also be used to access 3. The set of available P(v) derivatives 3 including P-stereogenic ones can be further expanded through replacement of suitable leaving groups Y (halide, alkoxide and even Ar) *via* path E.^11^ Analogous interconversions of cationic species 2, path F, play a pivotal role in highly selective substitution reactions at the phosphorus centre.^12^

In practice, there are several critical caveats associated with implementing certain reactions of the P-triangle. The first limitation is poor availability of mixed-group phosphines PR^1^R^2^R^3^ from phosphorus trihalides, especially in the case of compact, sterically innocent substituents R.^8^ As a redress for this, versatile alternative methods involving inverse polarity P–C coupling of phosphides have been introduced to access such mixed-group 1.^13^ However, these alternatives often suffer from unwanted quaternization of the desired P(iii) target compound, whereas analogous inverse polarity approaches to mixed P(v) structures 3 are generally less problematic.^14^ The second limitation is that the direct quaternization of phosphines 1, path A, is often quite slow and, for less reactive electrophiles, requires extreme conditions or the use of metal catalysts.^15^

The significance of the P-triangle to the present work is that it illustrates the relationships between direct and reverse interconversions of compound types 1–3. In almost all cases, the reverse reactions –A, –B and –C, are much more demanding than their forward counterparts, which makes their study both more challenging and potentially rewarding. Of these, by far the best known and studied is the deoxygenation –B which is thermochemically disfavored (due to the P=O bond enthalpy: 130–150 kcal mol^−1^ ^16^). This has been, and remains, a significant challenge in organophosphorus chemistry to which many workers,^17^ including ourselves^18^ have contributed solutions that have led to a variety of symmetrical and P-stereogenic 1 and 2.^19–21^

This study focuses on two additional reverse transformations. Although there are many efficient synthetic routes to compounds 1 and 2,^8–12,22^ the applications of reverse transformation “–A” remains underdeveloped. Classical electrochemical reductive cleavage of phosphonium salts to phosphines is well known,^9*a*,23,24^ but it is rarely applied preparatively for unsymmetrical cases due to uncertainty regarding which group is cleaved.^9*a*^ More recently, reports of debenzylation and dearylative reactions^25^ at phosphorus include examples of –A transformations that provide mixed-group compounds 1 with improved selectivity. The second transformation, –C, involves the conversion of phosphine oxides 3 to quaternary structures 2 (particularly where Y = R). This reaction would enable direct utilization of phosphine oxides, which are typically inert by-products of stoichiometric^10,12*a*–*e*,26^ and catalytic^19–21,27^ processes rather than reducing PO to P(iii) structures 1. Prior to this work, such a transformation had not been demonstrated.

**Scheme 2:**
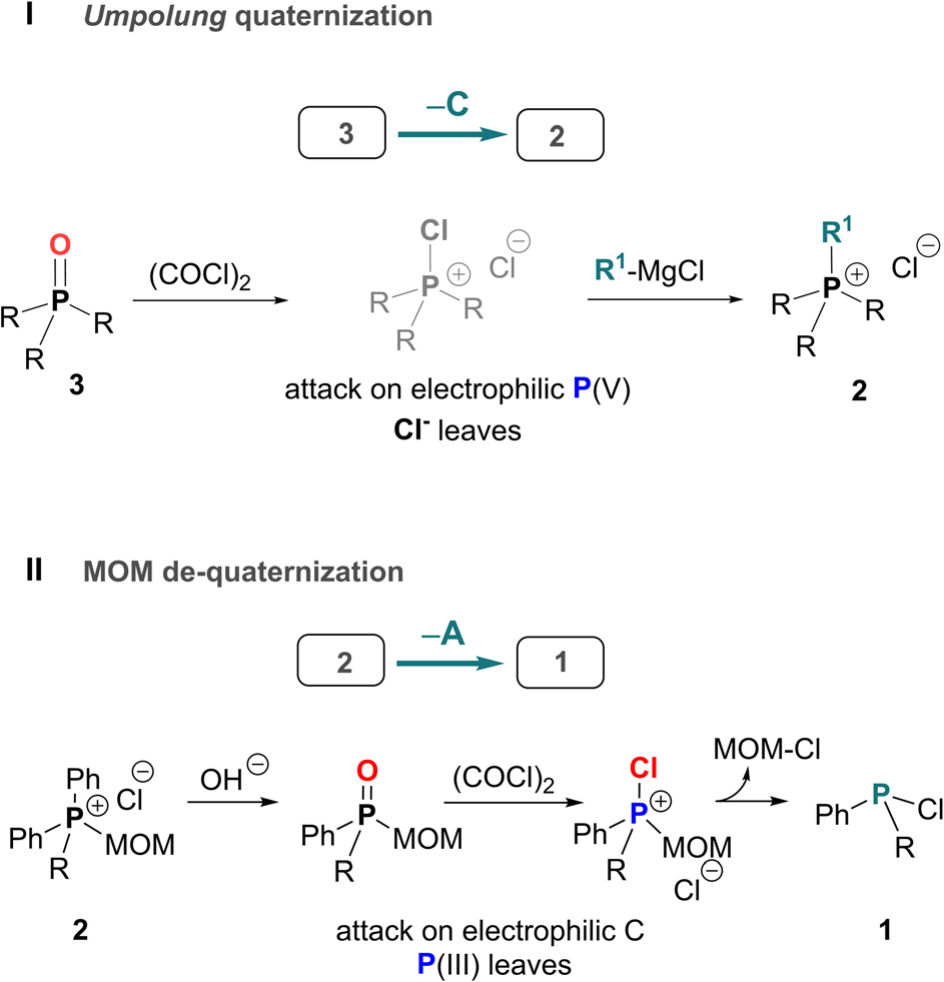
Novel reverse transformations presented in this report: (I) – Umpolung quaternization “–C” of phosphine oxides 3; (II) – spontaneous reverse quaternization “–A” (de-quaternization) of MOM-phosphonium salts 2.

Here, we present and develop solutions to the missing synthetic transformations –A and –C which arise through two new reactions discovered in our laboratory (Scheme 2(I and II)). The first new reaction, described in a preliminary communication,^28^ involves Umpolung quaternization of phosphine oxides 3. This transformation belongs to class –C shown in Scheme 1 and is a general reverse route that enables efficient conversion of phosphine oxides 3 into quaternary salts 2*via* a P-chlorophosphonium cation intermediate followed by treatment with a Grignard reagent. When combined with standard P–C bond cleavage such as hydrolysis or Wittig reaction, this approach allows controlled, stepwise interconversion between different types of achiral and P-stereogenic quaternary salts 2 and phosphine oxides 3.

The second transformation (Scheme 2(II)), involves a direct spontaneous de-quaternization (reversal of quaternization corresponding to path –A) of MOM-derived phosphonium cations 2. Hydrolytic treatment of the quaternary salt (MOM-protected phosphine), followed by chlorination and cleavage of the MOM–P bond (MOM deprotection), produces mixed-group P-chlorophosphines 1. This new high-yielding one-pot transformation enables stepwise replacement of the organic groups of triarylphosphines Ar_3_P with other organic groups and is at the core of our novel approach to formal organic group-substitution at phosphorus^25^ connecting the initial Ar_3_P-based triangle to other parallel P-triangles, including P-stereogenic variants. From an environmental standpoint, an important feature of these reverse transformations is that they use readily available starting materials such as the widely available Ph_3_P 1.0-a and its commonly discarded oxide Ph_3_PO 3.0-a.

## Results and discussion

### Design of reaction sequence to access mixed-group structures

**Scheme 3:**
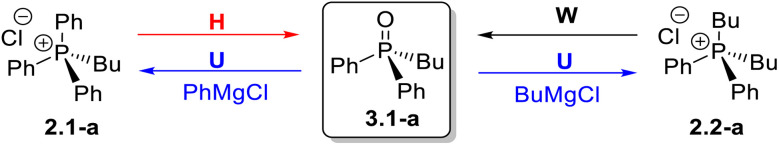
Complementary approaches to phosphine oxide 3.1-a using hydrolysis (H, red) or Wittig olefination (W, black) are mirrored by its Umpolung quaternization (U, blue) with different nucleophiles.

The Umpolung quaternization provides an effective route for constructing P–C bonds and, as demonstrated here, for synthesizing mixed aryl–alkyl P(v) compounds, including quaternary salts 2 and oxides 3. This approach can be combined with classical transformation “C”, involving selective cleavage of existing P–C bonds through hydrolysis^10*a*^ or Wittig reaction.^10*b*^ Controlled P–C bond cleavage has a long-established role^10*c*,*d*^ in organophosphorus synthesis and has been used to good effect recently.^10*e*–*g*^ Clearly, P–C cleavage gets the synthesis off the ground when combined with the Umpolung quaternization allowing the formation of mixed-group and P-stereogenic compounds in a controlled stepwise fashion. This process is exemplified by the transformation of Ph_2_n-BuPO 3.1-a into the two related phosphonium structures 2.1-a and 2.2-a, and back, as shown in Scheme 3.

The alkaline hydrolysis of phosphonium cation Ph_3_*n*-BuP^+^2.1-a (compound type PAB_3_) proceeds through hydrolytic cleavage (H, red arrow) of the group most stable as carbanion^10*a*^ – a Ph-substituent. In contrast, the Wittig olefination (W, black arrow) of the quaternary cation 2.2-a (type PA_2_B_2_) can only result in de-alkylation ultimately yielding the same parent oxide 3.1-a. Through Umpolung quaternization of 3.1-a, it becomes possible to regenerate either of the starting cations, 2.1-a or 2.2-a, from this common intermediate by employing two different Grignard reagents (U, blue arrows) in high yields.

**Scheme 4:**
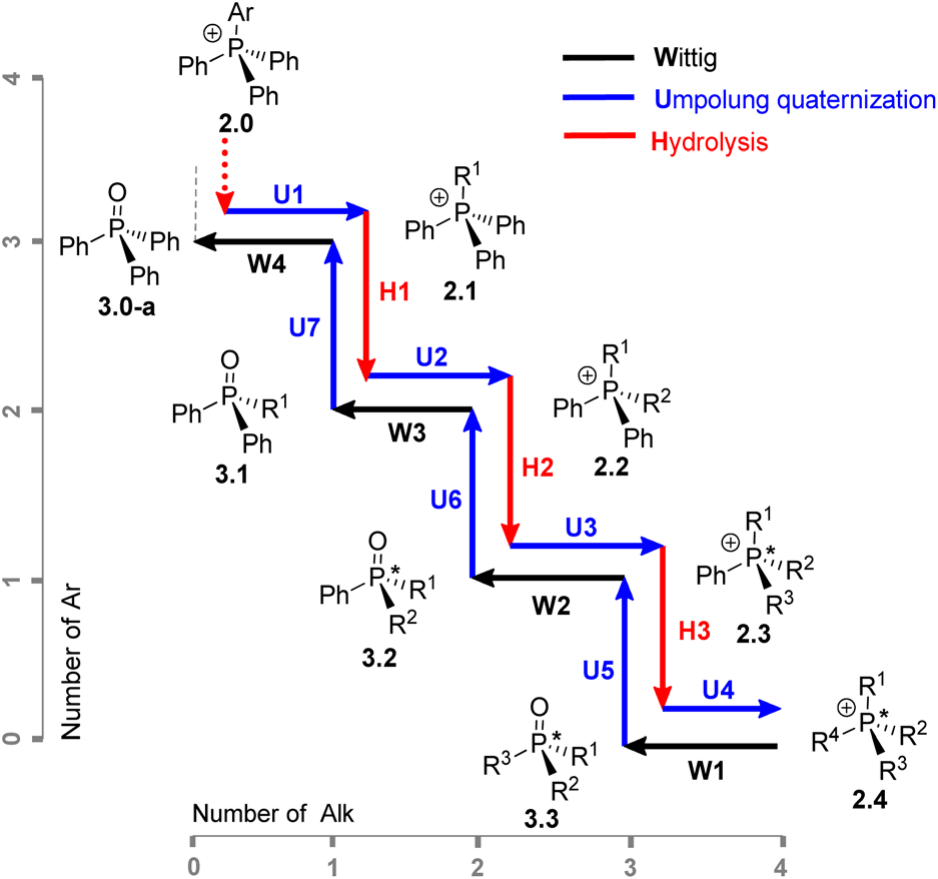
2D-sequence reaction featuring Umpolung quaternization in the aryl–alkyl coordinates provides access to a wide variety of quaternary phosphonium salts 2 and phosphine oxides 3 (digit after the dot indicates alkyl group count). P-stereogenic structures are marked with an asterisk.

The combination of H, W, and U steps provides a versatile synthetic approach for the controlled formation and cleavage of P–C bonds. This sequence enables access to a wide variety of mixed-group phosphonium salts and phosphine oxides from a single common precursor. To demonstrate the scope of this method, several examples of the sequence illustrated in Scheme 4 were carried out. The stepwise replacement of different groups can be viewed in the 2D aryl–alkyl space as shown in Scheme 4 (which we ironically termed the “Umpolung Walk” in our laboratory). The readily available Ph_3_PO 3.0-a was selected as the common starting material, a compound often regarded as a “phosphorus sink” in synthetic chemistry.^29^ The sequence of H, W, and U steps allows straightforward preparation of diverse phosphonium salts and phosphine oxides. Notably, the reactions proceed in high yield, exhibit good selectivity, and avoid the need to handle or isolate air-sensitive intermediates.

The first step of the sequence, U1, converts 3.0-a into a generic alkyltriphenylphosphonium salt Ph_3_R^1^P^+^Cl^−^2.1. Alkaline hydrolysis H1 of this salt yields Ph_2_R^1^PO (3.1), which serves as the substrate for a second quaternization U2, producing a mixed-group salt 2.2 (type PABC_2_). The subsequent hydrolysis step H2 affords a dialkylphenyl phosphine oxide PhR^1^R^2^PO (3.2), which becomes P-stereogenic when R_1_ ≠ R_2_. The following U3 step generates a P-stereogenic salt PhR^1^R^2^R^3^P^+^ (2.3), and the subsequent H3 and U4 steps yield the P-stereogenic oxide 3.3 and the tetraalkylphosphonium salt 2.4, respectively. This sequence of high-yielding quaternization and hydrolysis reactions enables the preparation of P-stereogenic compounds from the commonly discarded Ph_3_PO.

An alternative route, starting from the opposite end of the sequence, Alk_4_P^+^ (2.4), produces, *via* dealkylative olefination W1, the corresponding symmetrical phosphine oxide Alk_3_P(O) 3.3. This oxide can then be used to prepare phosphonium salts Ar^1^Alk_3_P^+^ (2.3, where Ar_1_ = Ph in Scheme 4) *via* the U5 reaction with an aromatic Grignard reagent Ar^1^MgCl. Extending this sequence through steps U6–U7 affords mixed aryl–alkyl salts Ar^1^Ar^2^Ar^3^AlkP^+^Cl^−^ (2.1), which upon olefination yield P-stereogenic triarylphosphine oxides 3.0 containing, in principle, three distinct aromatic groups Ar^1^–Ar^3^.

The structural diversity accessible through this approach is extensive and extends beyond the scope of the present study. The examples below provide a representative overview of the Umpolung transformation sequence, emphasizing substitution patterns that were previously difficult to achieve.

### Exploring the sequence from all-aryl structures 3.0

In our earlier report^28^ the quaternization U1 was examined and shown to be an effective method for forming P–C bonds, proceeding efficiently at low or ambient temperature to yield monoalkylphosphonium salts 2.1 of the general formula Ar_3_RP^+^Cl^−^. The present study extends this approach through Umpolung steps U2–U4, enabling the preparation of di-, tri-, and tetraalkylphosphonium salts 2.2–2.4, including asymmetric (P-stereogenic) derivatives.

**Scheme 5:**
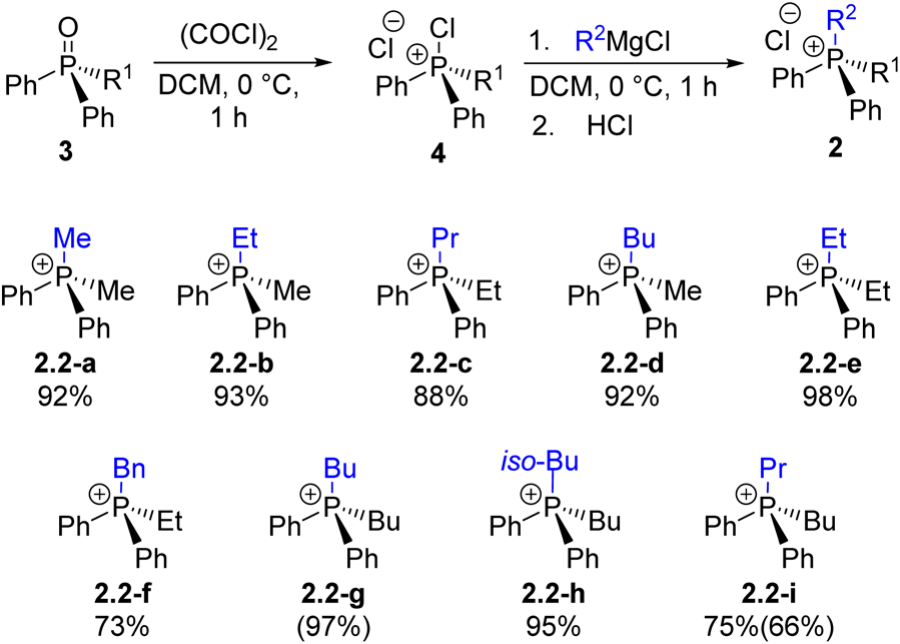
Exploration of step U2: preparation of quaternary phosphonium chlorides 2*via* the stage of chlorophosphonium species 4 followed by the introduction of an alkyl substituent R^2^; yield determined by ^31^P NMR spectroscopy (isolated yield).

Diarylalkylphosphine oxides 3.1a–3.1c were prepared quantitatively (R^1^ = *n*-Bu, Et, Me; see SI for details). These well-known 3.1 species were formally synthesized *via* step H1 and subsequently used in the quaternization U2 reaction to generate a series of mixed Ph_2_Alk_2_P^+^2.2 through the corresponding intermediates 4.1a–c (Scheme 5). As shown in Scheme 5, most U2 reactions produced the desired salts 2.2 in high yields. The slightly reduced yield observed for 2.2-f is likely due to partial deprotonation at the benzylic position. The U1–H1–U2 sequence demonstrates good scalability; for instance, compound 2.2-i was obtained in 75% yield on a 14 mmol scale by sequential introduction of *n*-Bu and *n*-Pr groups.

**Scheme 6:**
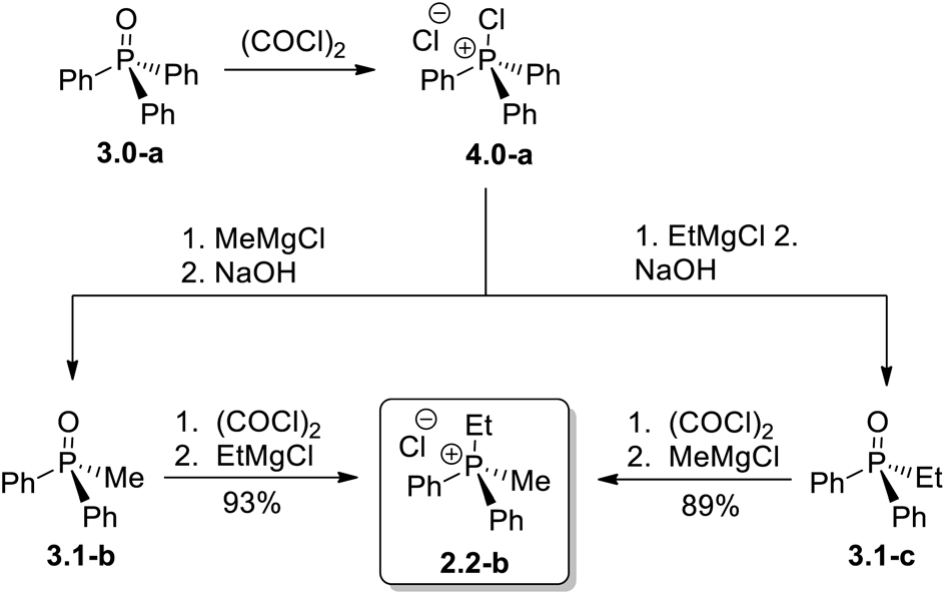
Two alternative Umpolung approaches to mixed dialkyldiaryl phosphonium chloride 2.2-b from the same parent phosphine oxide 3.0-a.

A representative example of the alternative routes available through the new transformation sequence is shown in Scheme 6 for compound 2.2-b. Notably, this target can be obtained in high yield through two distinct convergent pathways, both originating from the inexpensive and readily available Ph_3_PO. This outcome underscores the synthetic flexibility and efficiency of the Umpolung-based methodology.

### Formerly inaccessible synthetic targets

With the establishment of Umpolung-hydrolysis sequence (Scheme 4) described so far, a range of potential compounds can now be accessed using the U and H transformations. To illustrate this capability, several targets that were previously considered particularly challenging were selected for synthesis.

Previously, asymmetric (P-stereogenic) organophosphorus compounds have often featured PhMeP- or *tert-*BuMe-type motifs. While exceptions exist, these groups were favoured because they could be introduced relatively easily from readily available PhPCl_2_ or *t*-BuPCl_2_*via* the Mislow route^10*c*^ involving Arbuzov rearrangement of the corresponding phosphonous methyl (or ethyl) esters followed by nucleophilic displacement of the remaining ester group. This approach allowed the introduction of a single Me group (or Et with more difficulty) and overcame the substantial challenge of selectively substituting one chlorine atom in RPCl_2_ with a sterically unencumbered nucleophile. Incorporation of substituents other than Ph, Me, or *tert-*Bu required additional steps such as quaternization, hydrolysis, or Wittig reactions of the intermediate salts to phosphine oxides, followed by reduction to phosphines. These multistep processes often involved handling air-sensitive intermediates. The Umpolung sequence now removes many of these limitations.

Alkaline hydrolysis (H2) was applied to selectively cleave one aryl group from the mixed alkyl–aryl phosphonium salts 2.2-g and 2.2-i, generating phosphine oxides Ph*n*-Bu_2_PO (3.2-a) and Ph*n*-Bu*n*-PrPO (3.2-b) in high yield. The latter is an example of a species previously not easily accessible.^30^

The oxides 3.2-a and 3.2-b were then subjected to step U3*via* the corresponding chlorophosphonium intermediates 4.2-a and 4.2-b, using four different Grignard reagents to produce phosphonium salts 2.3-a through 2.3-d. As illustrated in Table 1, step U3 successfully furnished mixed-substituent phosphonium salts ArAlk_3_PCl. For example, reactions with *n*-BuMgCl and iso-BuMgCl yielded salts 2.3-a and 2.3-b (entries 1 and 2) in high yields, while the use of bulkier iso-PrMgCl (entry 3) resulted in a lower yield (60%) of 2.3-c. In contrast, the synthesis of the asymmetric phosphonium salt 2.3-d, containing a phenyl and three distinct *n*-alkyl groups, proceeded in high yield (entry 4). The overall yield of 2.3-d (68%) from Ph_3_PO over five steps, corresponding to an average of 93% per transformation, demonstrates the practicality and efficiency of the Umpolung sequence. The quaternary salt 2.3-a can also be obtained by quaternization of commercially available but air-sensitive *n*-Bu_3_P, whereas 2.3-b and 2.3-c would require prior synthesis of Ph*n*-Bu_2_P from PhPCl_2_. For compound 2.3-d, however, the quaternization U3 route we propose appears to be the only practical synthetic pathway.

**Table 1:** Efficient preparation of mixed-group quaternary salts 2.3 from phosphine oxides 3.2*via* the intermediate stage of 4.2

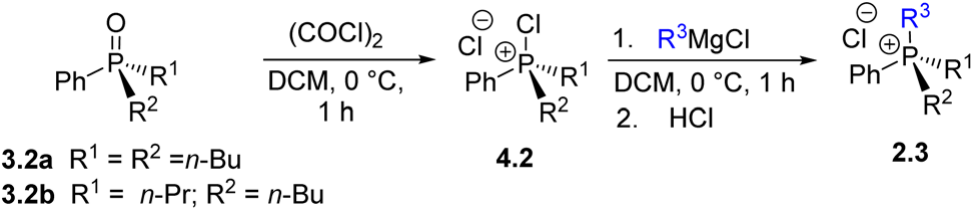					
Entry	R^1^	R^2^	R^3^	Product^a^	Yield^b^, %
1	*n*-Bu	*n*-Bu	*n*-Bu	2.3-a	89 (80)
2	*n*-Bu	*n*-Bu	iso-Bu	2.3-b	91 (84)
3	*n*-Bu	*n*-Bu	iso-Pr	2.3-c	60^c^
4	*n*-Bu	*n*-Pr	Et	2.3-d	90

a Using 2 equivalents of Grignard reagent, see SI for details.

b Yields determined by ^31^P NMR analysis, isolated yields in brackets.

c ^31^P NMR analysis indicated the presence of *n*-Bu_2_PhP 1.2-a (20%).

Steps H3 and U4 (Scheme 4) formally complete the Umpolung transformation sequence, leading to all-alkyl P-stereogenic structures 2.4 (Scheme 4, R^1^–R^4^ = Alk). Alkaline hydrolysis (H3) of 2.3-a (*n*-Bu_3_PhPCl) proceeded in high yield to give *n*-Bu_3_PO, which was then subjected to quaternization U4*via* the corresponding chlorophosphonium intermediate 4.3-a using a vinyl Grignard reagent, affording the trialkylvinyl cation 2.3-e in high yield.
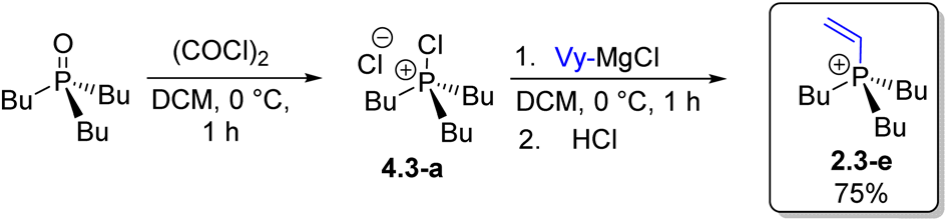


The example of the vinyl group is significant because vinylphosphonium salts are valuable intermediates in organic synthesis but are typically difficult to prepare.^31^ Standard quaternization of phosphines with vinyl halides is generally not feasible, except in certain cases under Pd catalysis. The main method of choice is Peterson reaction of alpha-silyl phosphonium ylides,^31^ which often produces Wittig-type side products and cannot be applied to the unsubstituted vinyl case achieved here.^32^ The Umpolung quaternization method now provides a more straightforward and general route to a variety of vinylphosphonium salts.

**Scheme 7:**
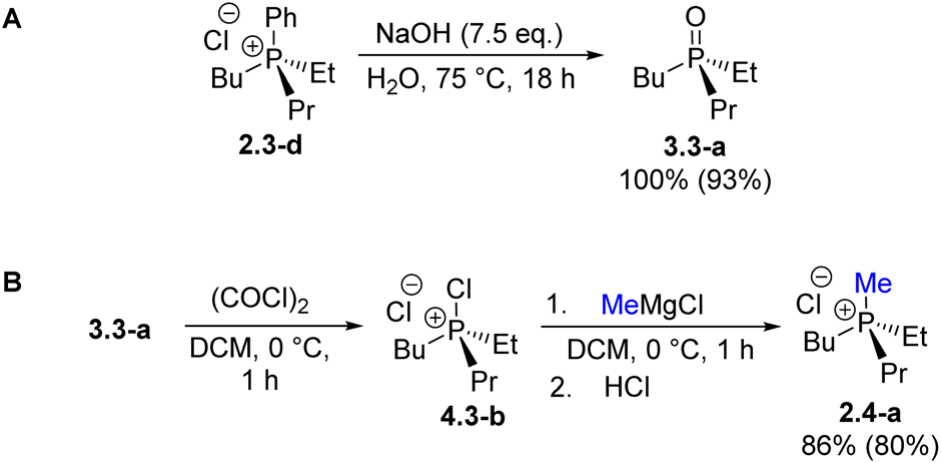
First synthesis of phosphonium salt 2.4-a. (A) hydrolysis of 2.3-d leads to the trialkyl structure 3.3-a. (B) Umpolung quaternization of 3.3-a furnished 2.4-a in high yield.

Alkaline hydrolysis, H3, of the asymmetric (P-stereogenic) 2.3-d required a 7.5-fold excess of NaOH at 75 °C resulting in selective cleavage of the Ph–P bond and formation of the phosphine oxide *n*-BuEt*n*-PrPO (3.3-a) in 93% yield (Scheme 7A). The transformation sequence can be accessed at multiple points (Scheme 4); the synthesis of 3.3-a described here proceeds in six steps from Ph_3_PO, while a shorter five-step route can be envisioned from commercially available Ph_2_PCl^33^ entering the sequence at compound 2.2 through classical organometallic treatment followed by quaternization.

The phosphine oxide 3.3-a enables access to uncommon P-stereogenic phosphonium structures of the all-alkyl type (PABCD). Umpolung quaternization (U4) of 3.3-a with methyl Grignard reagent shown in Scheme 7B afforded *n*-butyl(ethyl)(methyl)(*n*-propyl)phosphonium chloride 2.4-b in 86% yield (54% overall from Ph_3_PO). This represents the first authentic preparation of the smallest P-stereogenic tetra-*n*-alkylphosphonium cation.^34^ While the motivation for phosphonium structure 2.4-b may appear trivial, its synthesis *via* known quaternization procedures is challenging because the required P-stereogenic trialkylphosphines R^1^R^2^R^3^P are difficult to access even as racemic materials.

### Exploring the sequence from the all-alkyl structure 3.3

**Scheme 8:**
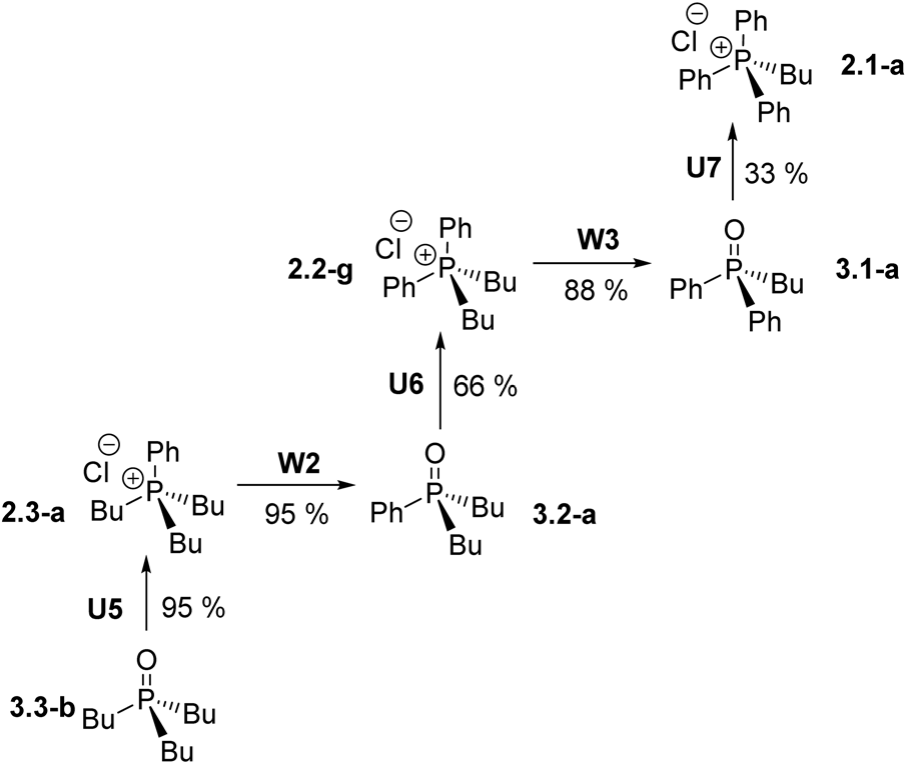
Exploration of the sequence featuring Umpolung quaternization from the all-alkyl side: alternative (see Scheme 4) preparation of phosphonium salts 2 and oxides 3 from symmetrical 3.3-b.

For illustrative purposes, we explored this solely with phenyl Grignard reagent. As shown in Scheme 8, the sequence begins with symmetrical tetraalkylphosphonium structures such as *n*-Bu_4_PCl (2.4-b). If desired, a simple olefination with acetaldehyde (W1) quantitatively affords *n*-Bu_3_PO (3.3-b). From this intermediate, reaction U5 with PhMgBr produces the phosphonium chloride 2.3-a in 95% yield,^28^ and its subsequent olefination W2 (see SI) proceeds in similarly high yield to give the corresponding oxide 3.2-a.

Further along the sequence, the unoptimized Umpolung steps show somewhat lower yields. For example, the diaryldialkylphosphonium salt 2.2-g was obtained in 66% yield (U6, Scheme 8), and its olefination produced the oxide 3.1-a in 88% yield. The final quaternization of 3.1-a (U7) afforded the triaryl structure 2.1-a in 33% yield. Although the later steps exhibit modest, unoptimized performance these results clearly demonstrate that the sequence proceeding from the all-alkyl structure 3.3 is feasible and it mirrors the transformations observed when starting from Ph_3_PO.

The Umpolung transformation sequence offers several advantages that challenge conventional expectations in synthetic organophosphorus chemistry. First, the reactions proceed as selective single-step processes, typically providing high yields of the desired products. Second, the methodology is highly flexible, allowing optimization of a specific synthetic route by varying the order of Umpolung quaternization U, olefination W and hydrolysis H steps. This adaptability enables alternative pathways that complement traditional synthetic strategies. A third and particularly important advantage is the self-limiting nature of each step: once the formation or cleavage of a specific P–C bond is achieved, the resulting products remain stable and do not undergo further transformation under the chosen reaction conditions. Another practical benefit is that both the target compounds and intermediates can be purified by standard recrystallization or chromatographic methods without the need for air-free techniques. Dry conditions are required only when conventional Grignard reagents are employed. Finally, the sequence relies on readily available and easy-to-handle starting materials such as Ph_3_PO or *n*-Bu_3_PO, further enhancing its practicality and accessibility for synthetic applications.

### Spontaneous reverse quaternization of certain MOM-phosphonium salts (transformation –A)

During the investigation of the Umpolung quaternization sequence, an important question arose: can methoxymethyl (MOM)-derived phosphine oxides 5 and quaternary phosphonium salts 6 serve as viable intermediates within the sequence? Until now, the use of MOM-derived phosphonium salts, such as Ph_3_PMOM^+^Cl^−^6.0-a, in synthesis has been largely confined to aldehyde homologation and chain-extension reactions.^35^ In this study, it is demonstrated for the first time that the MOM group can be readily removed as MOM–Cl, effectively acting as a temporary substituent at the phosphorus centre. This behaviour parallels the conventional protecting-group function of MOM in organic synthesis, establishing its role as a removable placeholder within the phosphorus framework.

**Scheme 9:**
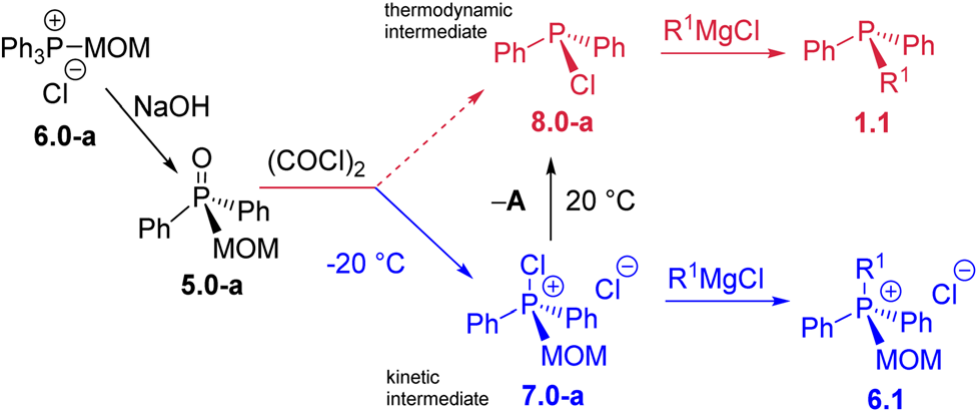
Unexpected P(v)–P(iii) dichotomy: at low temperature the kinetic P(v) intermediate 7.0-a leads to salts 6.1; at ambient temperature, the thermodynamic intermediate 8.0-a leads to monoalkylphosphines 1.1.

The MOM-derived 5.0-a (Scheme 9) was obtained in 95% yield *via* hydrolysis of the easily accessible^36^6.0-a. When 5.0-a was treated with oxalyl chloride at −20 °C (see SI) it furnished the corresponding kinetic product – chlorophosphonium species 7.0-a. At ambient temperature, this MOM-derived phosphonium cation underwent a spontaneous transformation yielding the well-known P(iii) derivative, chlorodiphenylphosphine 8.0-a as thermodynamic product, and releasing the free MOM–Cl. This reaction represents an unprecedented, facile collapse of a P(v) compound to a P(iii) chlorophosphine as the sole product, with no direct analogues.^37^ Unlike recently reported de-arylation processes,^25^ such clean unassisted de-alkylation at the phosphorus centre is the exact reversal of standard quaternization process A and thus corresponds to the “–A” pathway in Scheme 1. Due to its mechanistic resemblance to the classical Arbuzov collapse of alkoxyphosphonium species,^38^ the new –A process could also be termed a pseudo-Arbuzov collapse, as it involves nucleophilic cleavage of a C–P rather than a C–O bond. Treatment of 8.0-a with R^1^MgCl produces mixed-group phosphines 1.1 (the second “1” denoting one alkyl group R^1^). Conversely, at temperatures below 0 °C, where 7.0-a remains kinetically stable, reaction with R^1^MgX under Umpolung quaternization conditions affords new phosphonium structures 6.1.

This temperature-dependent behavior, illustrated in Scheme 9, can be described as an intriguing example of phosphorus oxidation-state thermal dichotomy.^39^ There is a notable parallel between the two alternative transformations of 7.0-a. At room temperature, the reaction pathway results in the net conversion of Ph_3_P (1.0-a) into monoalkyldiphenylphosphines 1.1, whereas at low temperature, the process proceeds from the MOM-protected triphenylphosphonium species 6.0-a to new quaternary structures 6.1 in which one phenyl group is replaced by an alkyl substituent. This dual reactivity underscores the fine balance between kinetic and thermodynamic control in phosphorus-centered transformations and highlights the versatility of the Umpolung framework in accessing distinct oxidation states and substitution patterns from a single precursor.

**Fig. 1:**
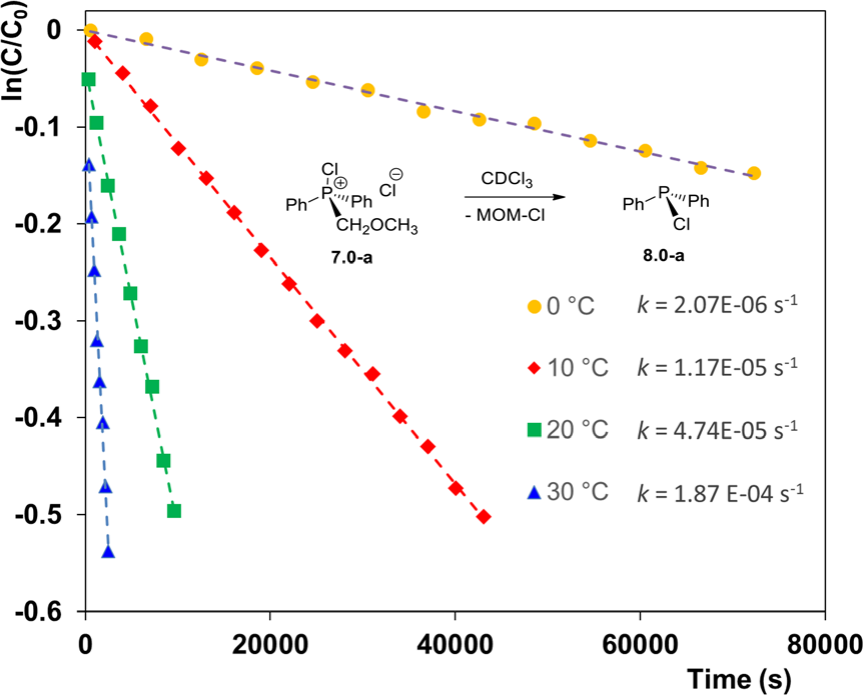
Pseudo-Arbuzov collapse of MOM-derived phosphonium chloride 7.0-a at different temperatures (solvent CDCl_3_).

The novelty of the pseudo-Arbuzov collapse and its potential synthetic significance warranted a more detailed study. When the collapse of 7.0-a was monitored by ^31^P NMR spectroscopy it demonstrated first-order kinetic as illustrated in Fig. 1. The reaction is dramatically accelerated at higher temperature such that at 30 °C (blue line) the rate constant *k*(30) = 1.87 × 10^−4^ s^−1^*i.e.* some 180 times greater than at 0 °C.

Eyring analysis of the kinetic data provided an activation enthalpy Δ*H*^‡^ = 24.0 kcal mol^−1^, which appears to be the dominant contributor to the activation barrier within the temperature range studied. This is consistent with a small positive activation entropy Δ*S*^‡^ = 3.7 cal K^−1^ mol^−1^, indicating that the transition state 7.0-TS is slightly more disordered than the relatively compact ion pair 7.0-a in the ground state.

**Fig. 2:**
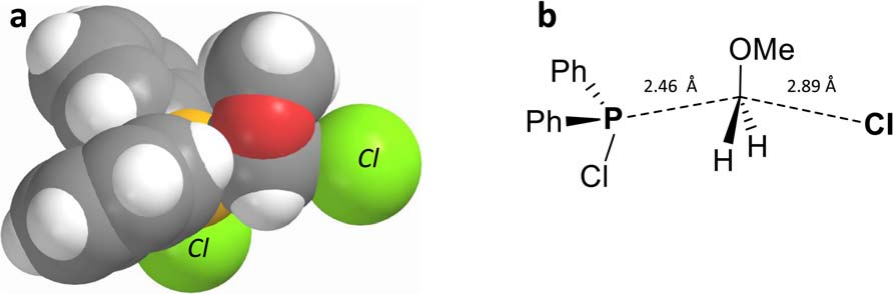
DFT (B3LYP-631G*) calculated structure of transition state 7.0-TS: (a) space-fill view; (b) the nearly planar MOM fragment is marked by significantly elongated P–C and C–Cl bonds.

This assumption is supported by the results of a DFT computational study of the transition state 7.0-TS (Fig. 2) which is characterized by a bent geometry with the bond angle P–C–Cl *ca.* 152° and elongated P–C and C–Cl bonds as shown. The more uniform charge distribution in the transition state is further evidenced by solvent effects: in more polar solvents, such as CH_3_CN, the reaction proceeds significantly more slowly than in CDCl_3_, yielding only a small amount of chlorodiphenylphosphine 8.0-a after 18 hours. Overall, these findings confirm that the pseudo-Arbuzov collapse of the MOM-derived chlorophosphonium species 7.0-a proceeds *via* an intramolecular nucleophilic mechanism, representing a well-defined transformation within phosphorus chemistry.

### Exploring the new reverse transformation –A: diverse reactivity of MOM derivatives

The spontaneous de-quaternization of 7.0-a (Scheme 8) complements the P-triangle (Scheme 1) with the missing reverse transformation –A. Here we show that this novel reaction is potentially general and, apart from a purely academic interest, is highly applicable as it leads from quaternary phosphonium P(v) structures back to P(iii) derivatives. Importantly, this means that collapse of mixed-group chlorophosphonium species 7.1 (“1” – one alkyl group) would afford non-symmetrical monochlorophosphines R^1^PhPCl 8.1 and can provide a selective route to P(iii) derivatives incorporating different small organic groups. Formerly, such species comprising sterically innocent groups were virtually inaccessible preparatively in good yields.^8*a*^

To examine this possibility, the required alkyl–aryl MOM-derived phosphine oxides 5.1 were prepared by MOM-protection of alkyldiphenylphosphines 1.1-a (R^1^ = Me) and 1.1-b (R^1^ = Et) followed by hydrolytic cleavage of the Ph. Treatment of 5.1-a/b with oxalyl chloride (Table 2) quantitatively gives methyl-, 7.1-a, and ethyl-substituted 7.1-b. The thermal collapse of the ethyl species 7.1-b was too slow at ambient temperature (Table 2, entry 1) and at 40 °C. The unwanted P(v) compound 9.1-b was mainly observed using dry chloroform as solvent (entry 2). Switching to the less polar benzene and toluene (entries 3 and 4) led to improved conversion to chlorophosphine EtPhPCl 8.1-b. However, using thoroughly degassed chloroform as solvent the collapse gave 8.1-b in excellent yield (sealed tube, 90 °C, 6 h, entry 5). Under such conditions, the collapse of Me-substituted 8.1-a gives chloride PhMePCl 9.1-a in 95% yield too (95 °C, 18 h, entry 6). These findings are consistent with the earlier observation that, in chloroform solution, chlorinated phosphonium species tend to exist in the form of reactive ion-pairs.^28*a*^

**Table 2:** Formation and collapse of MOM-derived alkyl–aryl species 7.1^a^

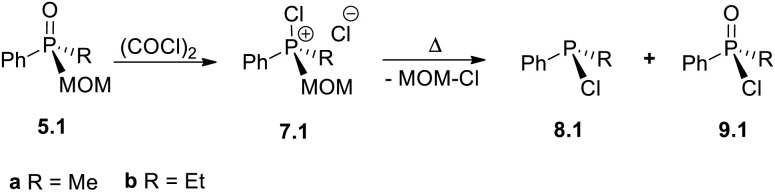							
Entry	R	Solvent	*t*, h	*T*, °C	7.1^b^, %	8.1, %	9.1,%
1	Et	CDCl_3_	18	25	98	0	2
2^c^	Et	CDCl_3_	16	40	47	6	(26)
3	Et	C_6_D_6_	96	60	4	61	(6)
4^d^	Et	C_7_H_8_	24	75	9	79	(12)
5^d^	Et	CDCl_3_	6	90^f^	5	93	(4)
6^d^	Me	CDCl_3_	5	95^f^	5	81	(14)
7^e^	Et	SOCl_2_	12	75	8	0	(92)
8^e^	Me	SOCl_2_	12	75	3	0	(97)

a 7.1 generated by using COCl_2_ or SOCl_2_ in sealed tube.

b Determined by ^31^P NMR analysis of reaction mixture.

c Using 1.5 eq. (COCl)_2_.

d Using 1.2 eq. (COCl)_2_.

e Using excess SOCl_2_.

f Sealed tube.

**Scheme 10:**
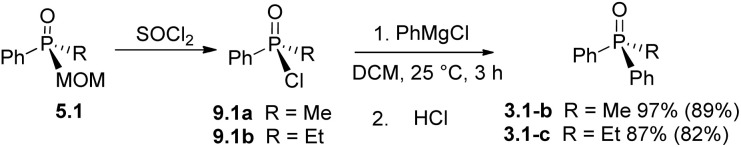
Oxidative MOM-deprotection of 5.1 followed by introduction of a Ph-group.

Remarkably, the unwanted formation of phosphinic chlorides 9.1 by oxidative MOM-deprotection, can become dominant: switching the source of chlorine to SOCl_2_ gave 9.1-b and 9.1-a virtually quantitatively (entries 7 and 8). This new unusual transformation corresponds to another interesting sub-class which can be termed “–E” (Scheme 1) because, it can be argued, it entails a conversion of a MOM-derived 5.1 into a phosphinic chloride 9.1*i.e.* formal replacement of an alkyl group (MOM) with a chlorine. Here, again, we are looking at a reversal of standard reactivity as phosphinic species 9.1 themselves are commonly used for accessing phosphine oxide 5.1. Accordingly, treatment of 9.1 with PhMgCl for example (Scheme 10) leads to mixed-group tertiary phosphine oxides 3.1-b and 3.1-c in high yields. Oxidative MOM-deprotection using SOCl_2_ as an alternative reagent further broadens the scope of this methodology. Depending on the chlorination reagent employed, either P(iii) chlorinated phosphines 8 or the corresponding P(v) phosphinic chlorides 9 can be obtained from MOM-derived phosphine oxides 5 in high yields.

**Scheme 11:**
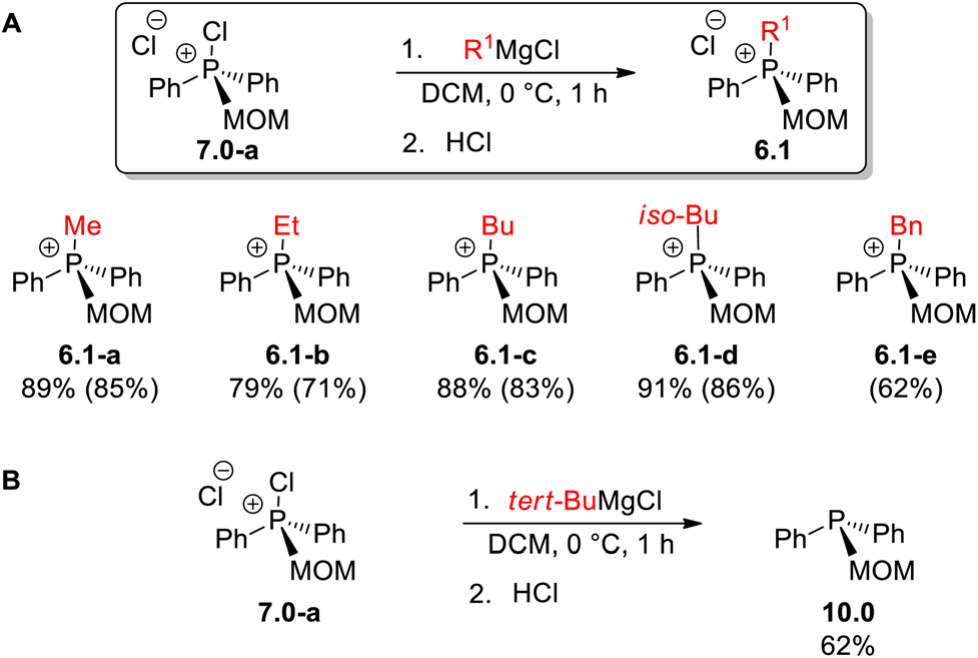
Umpolung quaternization: (A) preparation of MOM-substituted 6.1; (B) preparation of MOM-substituted 10.0.

The synthetic versatility of MOM-derived intermediate 7.0-a was further examined in the Umpolung quaternization to prepare MOM-substituted phosphonium salts (Scheme 11A). When 7.0-a was treated with alkylmagnesium chlorides at 0 °C, the mixed-substituent structures 6.1a–d were obtained, mostly in high yields; for instance, the reaction of iso-BuMgCl leads to 6.1-d in 91% yield (86% isolated).

Importantly, all quaternary phosphonium products were isolated by easy crystallization, including 6.1-e (62% isolated). Remarkably, the reaction of 7.0-a with *tert*-BuMgCl gave MOM-derived phosphine 10.0 (Scheme 11B) providing a new route to the poorly explored tertiary phosphines containing an alkoxyalkyl group at the phosphorus center. We believe this phosphine is produced *via* an attack^28*a*^ of the Grignard reagent on the electron-deficient covalently bound chlorine of 7.0-a.

**Scheme 12:**
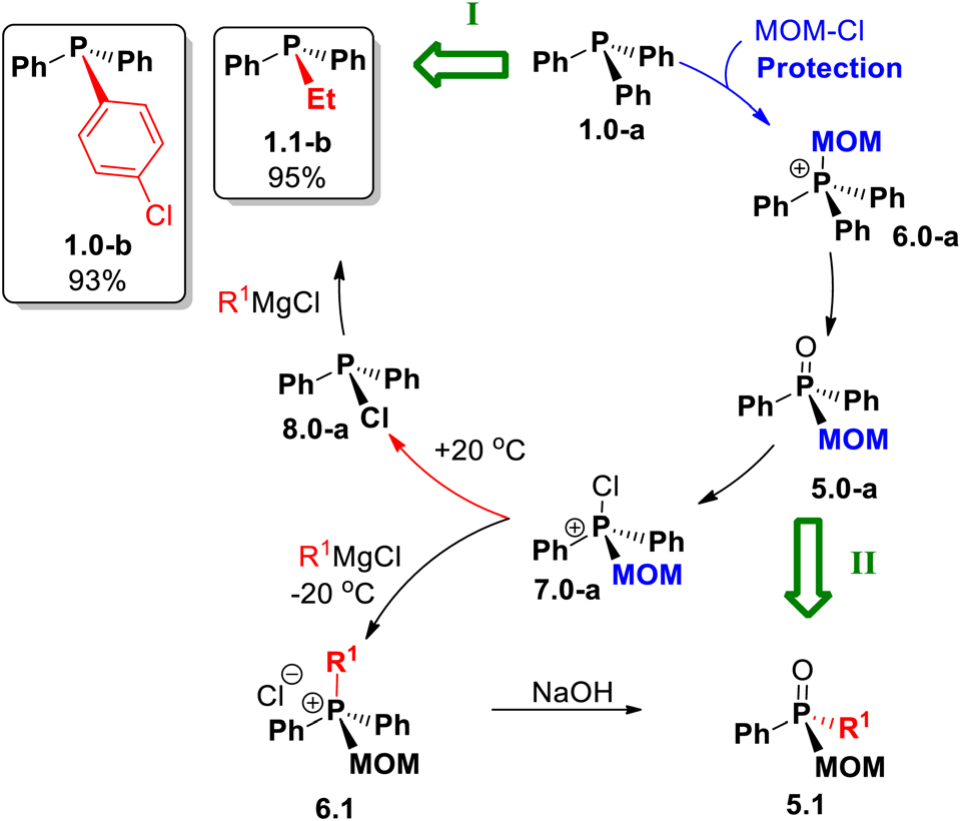
Efficient net transformations (green block arrows) of Ph_3_P enabled by MOM: (I) alkyl–aryl phosphines Ph_2_P–R^1^*via* the room temperature route. (II) mixed-group phosphine oxides 5.1 at low temperature.

Turning now to new synthetic opportunities presented by MOM-protection, a variety of compounds can now be made from triphenylphosphine 1.0-a as the starting point. As summarized in Scheme 12, this basic structure can be converted to mixed-group tertiary phosphines Ph_2_RP, 1.1, *via* the stages of MOM-derived 5 and 6 followed by MOM-deprotection of the key chlorinated intermediate 7.0-a. Finally, standard replacement of Cl affords phosphines 1.1-b and 1.0-b in high yields of 95% and 93%, respectively, based on the original Ph_3_P. Crucially, using MOM-derived chlorophosphonium compound 7.0-a, a different reaction sequence (path II, Scheme 12) leads, *via* the stage of quaternary 6.1, to MOM-derived phosphine oxides 5.1.

**Scheme 13:**
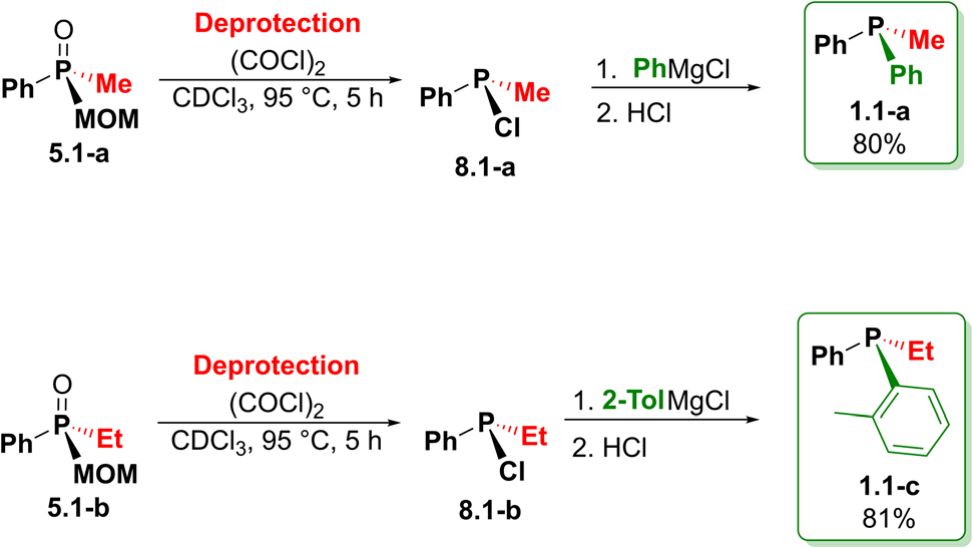
Two-step synthesis of phosphines enabled by MOM-deprotection of 5.1 (see Scheme 12).

The mixed-group phosphine oxide 5.1, as a key intermediate, opens the access to even more structures shown in Scheme 13.

As a prototypical example, the oxide 5.1-a, after chlorination–deprotection, leads to P(iii) compound 8.1-a and, upon treatment with PhMgCl, gives the mixed-group phosphine 1.1-a in high overall yield (80%). Of course, in this example the re-introduction of a Ph group is degenerate and simply demonstrates the viability of a new MOM-protection approach. Significantly, preparation of the P-stereogenic 1.1-c from 5.1-b clearly demonstrates the synthetic value of the new MOM chemistry. Here, MOM-deprotection (*via* collapse of 7.1-b) leads to chlorophosphine 8.1-b (93% yield, Table 2) which, upon treatment with *o*-tolylmagnesium chloride, affords 1.1-c. Overall, this P-stereogenic phosphine has been prepared from symmetrical Ph_3_P in 56% yield over six steps (93% per step on average).

The results presented here are significant for phosphorus chemistry in two ways: firstly, the previously unknown pseudo-Arbuzov thermal collapse of MOM-derived chlorophosphonium species 7 leading to P-chlorophosphines is distinctly different from other organophosphorus chemistry reactions. It is highly complementary to the existing transformations forming the P-triangle (Scheme 1) as it belongs to the poorly explored reverse quaternization processes “–A”. Secondly, since the new route to P-chlorophosphines is selective and high yielding, it is very valuable in the design of new pathways to access a variety of asymmetric and P-stereogenic phosphines.

### MOM-enabled sequential strategy

**Scheme 14:**
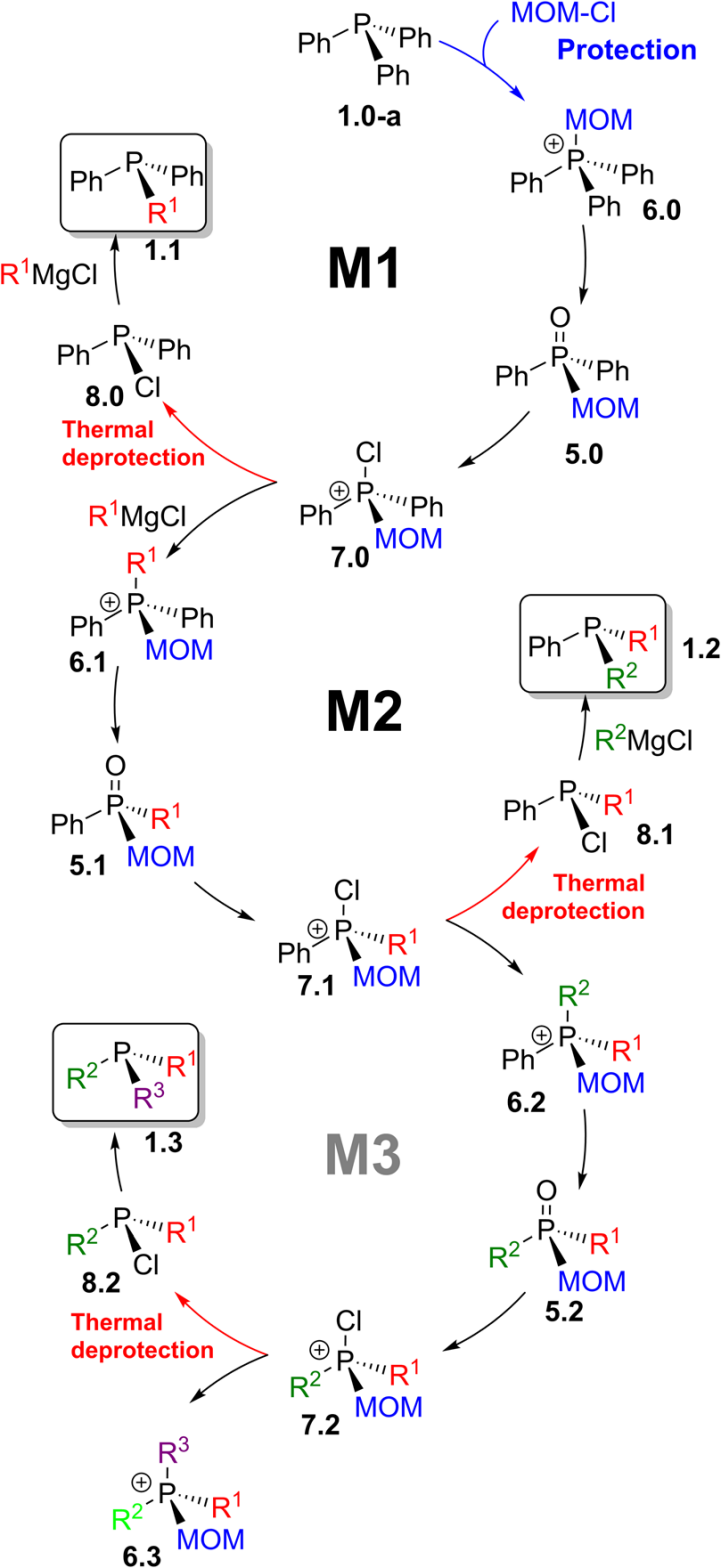
MOM-enabled chemistry: sequence M1 leads to phosphines 1.1; M2 gives stereogenic 1.2; M3 is a potential route to trialkylphosphines 1.3.

A broader synthetic framework was developed as an extension of the transformations shown in Scheme 12. It is represented in Scheme 14 as a sequence of interconnected reaction cycles. Starting with triphenylphosphine 1.0-a, through the first cycle (M1), the pathway reaches compound 7.0 – thermal branching point I. At elevated temperature, this route affords mixed tertiary phosphines 1.1, whereas maintaining the temperature below 0 °C leads, *via* inverse quaternization with Grignard reagents, to the MOM-derived phosphonium structure 6.1.

This compound proceeds through the next cycle (M2), where a similar sequence of reactions leads to 7.1 – another thermally controlled branching point II. Upon heating, chlorinated intermediate 7.1 yields 8.1, which in turn produce mixed tertiary phosphines 1.2. Alternatively, at lower temperature, new mixed-group MOM-derived phosphonium species 6.2 are formed. These serve as entry point to another proposed cycle (M3), extending the methodology toward more extensively substituted phosphines 1.3 in which all three alkyl groups are introduced through successive replacement of phenyl substituents in Ph_3_P 1.0-a.

This synthetic approach relies on MOM-quaternization of phosphines 1, hydrolysis of phosphonium salts 6, and Grignard reagent metathesis with P-chlorophosphines 8. Fundamentally, by combining the Umpolung quaternization step (Scheme 2, A) with MOM-based protection–deprotection, symmetrical triphenylphosphine and its oxide can be efficiently transformed into a wide range of mixed-group and P-stereogenic phosphines 1.2 and 1.3 in a few steps.

## Conclusions

The new systematic approach to the synthesis of P(v) compounds features high-yielding reverse transformations combined with established reactions of organophosphorus chemistry. Centred on the Umpolung quaternization process –C, this methodology enables the preparation of diverse mixed and P-stereogenic phosphine oxides and phosphonium salts from readily available and often discarded triphenylphosphine oxide using inexpensive and convenient reagent–condition combinations. The synthesis of the smallest P-stereogenic quaternary tetraalkylphosphonium structure from triphenylphosphine oxide exemplifies access to a fundamental chemical structure that was previously difficult or impossible to obtain.

In addition, the key elements of this approach were combined with an unprecedented reverse quaternization of MOM-derived species –A. This process provides an efficient route from MOM-derived tertiary phosphine oxides to chlorinated P(iii) compounds. For the first time, a multi-step protection–deprotection strategy was achieved at the nucleophilic phosphorus centre using standard MOM–Cl as the protecting reagent which can be recycled.

The missing connections, –A and –C, between the three key classes of organophosphorus compounds 1, 2 and 3 have been added to the P-triangle (compare to Scheme 1) as follows:
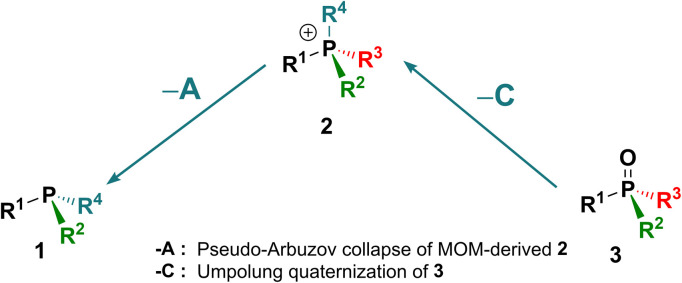


This simple diagram illustrates how the new findings complete the existing network of organophosphorus transformations by introducing two reverse processes. The reactions are complementary: path –A is a P–C bond-breaking process, while path –C is a P–C bond-forming process. Moreover, the P–C bond created in process –C is typically not the same bond cleaved in reaction –A, making their combination a powerful synthetic tool for constructing diverse organophosphorus architectures.
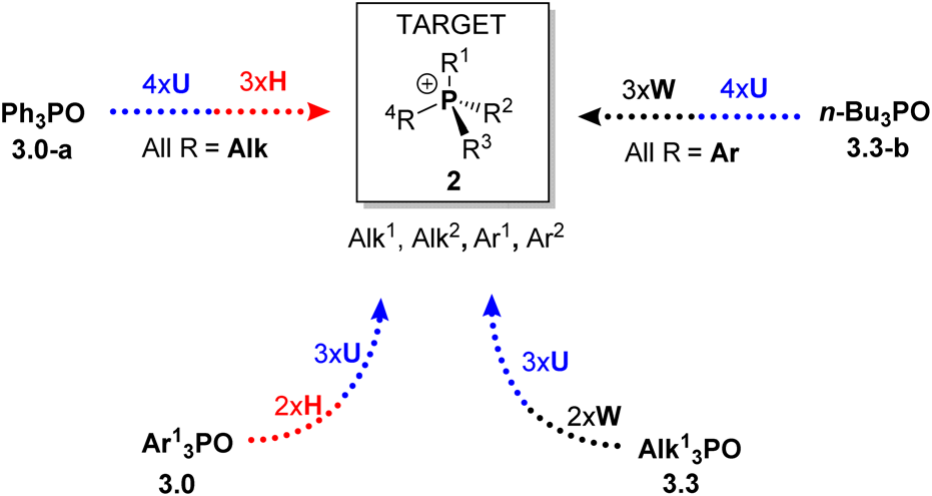


For instance, new structures R_4_P (where R represents alkyl or aryl groups, or =O for phosphine oxides 3) can be synthesized through iterative application of U and H steps. All-alkyl species 2 can be obtained from Ph_3_PO (3.0-a) *via* four U and three H steps, while all-aryl structures can be accessed from symmetrical *n*-Bu_3_PO (3.3-b) using U and W steps. For mixed structures lacking Ph or *n*-Bu substituents, the corresponding symmetrical triaryl 3.0 or trialkyl 3.3 derivative can serve as the starting point, following then the same iterative approach.

In summary, two distinct and complementary missing links have been established between the principal classes of organophosphorus compounds. Through these transformations, many mixed aryl–alkyl phosphines can be prepared from Ph_3_P, and asymmetric (P-stereogenic) quaternary phosphonium salts can be synthesized from Ph_3_PO and other phosphine oxides in only a few steps. These new synthetic tools provide straightforward access to a wide range of mixed and P-stereogenic phosphines, their oxides, and phosphonium salts from inexpensive and widely available phosphorus precursors. The results presented here, together with earlier findings, indicate that these transformations open extensive new possibilities in organophosphorus chemistry, with broad potential for the synthesis of valuable P(iii) and P(v) target compounds.

## Author contributions

Conceptualization and design: A. C. V., K. N., D. G. G. Data collection: A. C. V., Y. O. (supporting), K. N.; Writing: A. C. V., K. N., DGG (supporting); supervision and funding acquisition: D. G. G.

## Conflicts of interest

There are no conflicts to declare.

## Supplementary Material

SC-OLF-D5SC04496K-s001

## Data Availability

The data supporting this article have been included as part of the supplementary information (SI). Supplementary information: details of experimental methods, characterisation data. See DOI: https://doi.org/10.1039/d5sc04496k.
