# Supplementary material for: The organophosphorus synthesis triangle: introducing methods for the missing quaternization and de-quaternization routes
Source: Chem Sci. 2025 Oct 24;17(1):164–75. doi: 10.1039/d5sc04496k (PMC12550599; doi:10.1039/d5sc04496k)
Supplement: SC-017-D5SC04496K-s001 [file SC-017-D5SC04496K-s001.pdf]

Supplementary Information (SI) for Chemical Science.
This journal is © The Royal Society of Chemistry 2025

Supporting Information

The Organophosphorus Synthesis Triangle: Introducing Methods for the Missing Quaternization and De-quaternization Routes

Anna C. Vetter, Yannick Ortin, Kirill Nikitin* and Declan G. Gilheany*

[School of Chemistry, University College Dublin, Belfield, Dublin 4, Ireland

Contents

1. **General** 2
2. **Umpolung Walk** 4
3. ***P*-Chlorophosphonium Chlorides: Characterization Data** 6
4. **Quaternary Phosphonium Salts 2: Synthesis and Characterization Data** 8
5. **Phosphine Oxides: Synthesis and Characterization Data** 26
6. **MOM-derived Quaternary Phosphonium Salts** 33
7. **Preparation of MOM-derived phosphine oxides** 40
8. **MOM-derived *P*-chlorophosphonium salts** 43
9. **Phosphines: Synthesis and Characterization Data** 49
10. **Computational Data** 53
11. **References** 55

1. General techniques

1.1 Experimental

All commercially available solvents were used as supplied unless stated otherwise. All “dry” solvents were dried and distilled by standard procedures or were processed through a Grubbs-type *PureSolv-400-3-MD* solvent purification system. Dry degassed solvents were stored in Young-type flasks over molecular sieves 4Å (MS) which were activated as follows: in a Schlenk, Young’s or Strauss flask the as-received MS were heated over a butane gas Bunsen burner for two minutes and then placed under reduced pressure and allowed to cool. This cycle was repeated twice. The water content in all solutions as monitored by titration on an *Aquamax CouLo* instrument was less than 10 ppm v/v. Oxygen-free nitrogen was obtained from *BOC gases* and passed over dry MS.

All reactions were performed in a dry Schlenk tube, Young’s flask or tube, crimp-cap vial or an NMR tube under nitrogen using Schlenk technique. All glassware but NMR tubes was flame-dried prior to use. Air/moisture sensitive liquids and solutions were transferred *via* nitrogen-flushed plastic stopper less syringes 1-20 mL as appropriate. For amounts less than 0.100 mL gas-tight 25, 50 and 100 microliter teflon-capped micro syringes were used.

Flash column chromatography was performed on *Davisil* particle size 0.040-0.063 mm. Mass spectra were run on an Agilent 6546 QTOF Mass Spectrometry system equipped with an AJS (Agilent Jet Stream) ESI source and Agilent 1260 Infinity Prime II LC system. Mass spectrometry reported in this publication was supported by The Comprehensive Molecular Analysis Platform (CMAP) under The SFI Research Infrastructure Programme, reference 18/RI/5702. NMR spectra were recorded on *Agilent* VNMRS spectrometers at 25 °C. Chemical shifts are reported as δ-values in ppm relative to internal standard tetramethylsilane (TMS) for ^{1}H and ^{13}C NMR spectra. ^{31}P NMR chemical shits are relative to an internal pre-calibrated standard. Coupling constants (*J*) are reported in hertz (Hz). ^{31}P NMR spectra recorded using a relaxation delay ≥ 3 seconds with suppression of heteronuclear NOE. Peak integrations were determined by using a MestreNova software package. Assignments were based on standard ^{1}H-^{1}H and ^{1}H-^{13}C two-dimensional techniques such as HSQC, HMBC and COSY. Where full structures were assigned, the experimentally observed chemical shifts (as determined through the 2D experiments) are depicted in the structures provided. Where multiplicities were observable in the 1D experiments, these are included in the signal listings.

1.2 Alphanumerical coding of the compounds

The proposed methodology interconnects three major classes of compounds (Scheme 1, main text). This led us to the concept of a meaningful coding convention for compounds in this report. We have adopted it as follows:

1. a single numerical code signifies **a class** of compounds as shown in Scheme 1 (e.g. **3** for phosphine oxide) or a group of compounds pertaining to this class.
2. a two-digit code **X.Y** with a dot separator signifies a subtype of compounds and the numerical on the right of the dot stands for the **number of alkyl groups** attached to the P centre
3. a code containing a dash signifies one **specific structure**. Example: **3.1-c** means "phosphine oxide containing one alkyl group, compound c".
4. In cases where only *one alkyl* is attached to the P centre, **a** stands for n-butyl, **b** for Me and **c** for ethyl. For example, compound **3.1-c** is

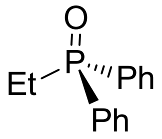

2. **Umpolung Walk**

2.1. General procedure A: preparation of *P*-chlorophosphonium salt (CPS) **4** stock solutions from phosphine oxides **3**

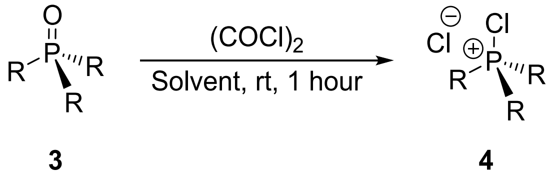

Unless stated otherwise, all *P*-chlorophosphonium salts **4** (typically 40-60 mL of a 0.2 M solution) were prepared at ambient temperature as follows: a 100-mL Young's flask equipped with a stir bar was charged with phosphine oxide **3** (1.00 equivalent). The material was dried under vacuum and enough dichloromethane (DCM) was added to make a 0.2 M solution of **4**. This was followed by dropwise addition of oxalyl chloride (1.02 equivalents) via an evacuated, nitrogen-flushed syringe. Typically, effervescence was immediately observed, which ceased within one hour. After this time, ^{31}P NMR spectroscopy indicated complete conversion of **3** to **4.**

2.2. General procedure B: preparation of quaternary phosphonium salts (QPS) **2** from **4** (reactions **U1-U7**, Scheme 4 Main text):

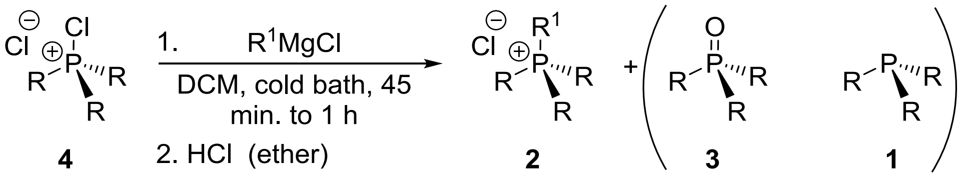

A 50 mL Schlenk tube equipped with a stir bar was charged with a 0.2 DCM solution of **4** (1.00 equivalent) and cooled to the specified reaction temperature. A solution of the appropriate organomagnesium chloride in THF or ether (R^1MgCl, 2.00 equivalents) was added slowly, typically over the course of five minutes, down the side of the flask. The resulting solution was stirred at the specified temperature for 45 minutes to one hour. After this time, the reaction mixture was quenched by the addition of a 2.0 M solution of HCl in diethyl ether (2.00 equivalents) and stirred for ten minutes prior to being removed from the cold bath (where applicable).

Analysis: a small volume (ca. 0.4-0.5 mL) of the quenched reaction mixture was withdrawn, the solvent removed *in vacuo* and $CDCl_3$ (1.00 mL) was added to give, after filtration through a plug of Celite on cotton wool (to remove insoluble inorganic salt), a transparent solution containing QPS **2** and, where applicable, side products.

Workup: the reaction mixture was washed with brine typically 3 times. The organic layer was dried with sodium sulfate, evaporated to give an oily residue, which in most cases crystallised to form **2** which could be recrystallized from chloroform/ethyl acetate.

2.3. General procedure **C** (reactions **H1-H3**, Scheme 4 Main text): preparation of phosphine oxides **3** via hydrolysis of quaternary phosphonium salts **2**

To an appropriately sized flask, quaternary phosphonium salt **2** (1.00 equivalent) was added. Under vigorous agitation, an aqueous solution of NaOH (typically 5 M, 5 equivalents, but varied as noted in each case) was added and the mixture stirred, typically at ambient temperature for 18 hours but sometimes requiring heating as noted in each case. After this time, dichloromethane (ca. 2 mL/mmol QPS) was added, and the aqueous layer was extracted. Extraction was repeated twice more, and the organic phases combined, dried over magnesium sulfate and concentrated *in vacuo* to give **3** as a solid/oil which typically crystallized on standing.

2.4. General procedure **D** (reactions **W1-W4**, Scheme 4 Main text): preparation of phosphine oxides **3** via olefination of quaternary phosphonium salts **2**

To a flame-dried Schlenk tube, quaternary phosphonium salt **2** (1.00 equivalent) was added and the material was dried under vacuum at 80 °C. Under nitrogen, dry THF (5.00 mL per one mmol of **2**) was added to give a 0.2 M suspension of the salt, which was cooled to -25 °C. To this, KHMDS (1.0 M solution in THF, 1.00 eq.) was added and the resultant mixture was stirred at -25 °C for 30 minutes, when the reaction mixture appeared strongly orange, acetaldehyde (1.05 eq.) was added, causing the orange colour to fade instantly and the mixture was stirred for 1 hour at -25 °C. After this time, the flask was removed from the cold bath and the solvent and alkene product removed in vacuo to give product **3** which can be purified by crystallisation or chromatography.

3. *P*-Chlorophosphonium Chlorides: Characterization Data

3.1. *P*-Chlorotriphenylphosphonium chloride **4.0-a** from **3.0-a**

This was prepared and characterized as described elsewhere.[1]

3.2. *P*-Chloro(methyl)diphenylphosphonium chloride **4.1-b** from **3.1-b**

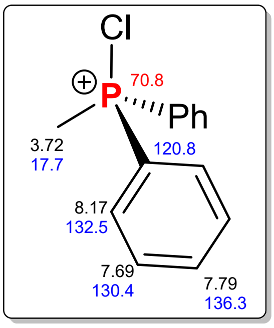

^{31}**P NMR** (162 MHz, $CDCl_3$) δ 70.8 ppm. ^{1}**H NMR** (400 MHz, $CDCl_3$) δ 8.17 (dd, *J* = 15.0, 7.0 Hz, 4H), 7.79 (td, *J* = 7.0, 2.0 Hz, 2H), 7.69 (td, *J* = 7.0, 5.0 Hz, 4H), 3.72 (d, *J* = 13.0 Hz, 3H) ppm. ^{13}**C NMR** (101 MHz, $CDCl_3$) δ 136.3 (d, *J* = 3.0 Hz), 132.5 (d, *J* = 14.0 Hz), 130.4 (d, *J* = 15.0 Hz), 120.8 (d, *J* = 92.0 Hz), 17.7 (d, *J* = 53.0 Hz) ppm.

3.3. *P*-Chloro(ethyl)diphenylphosphonium chloride **4.1-c from 3.1-c**

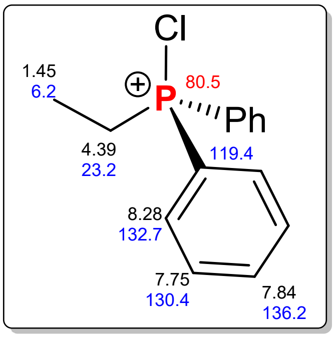

^{1}**P NMR** (162 MHz, $CDCl_3$) δ 80.5 ppm. ^{1}**H NMR** (400 MHz, $CDCl_3$) δ 8.28 (dd, *J* = 15.0, 7.0 Hz, 4H), 7.84 (td, *J* = 7.0 Hz, 2H), 7.75 (td, *J* = 7.0, 5.0 Hz, 4H), 4.39 (dq, *J* = 7.0, 7.0 Hz, 2H), 1.45 (dt, *J* = 25.0, 7.0 Hz, 3H) ppm. ^{13}**C NMR** (101 MHz, $CDCl_3$) δ 136.2 (d, *J* = 3.0 Hz), 132.7 (d, *J* = 12.0 Hz), 130.4 (d, *J* = 15.0 Hz), 119.4 (d, *J* = 87.0 Hz), 23.2 (d, *J* = 44.0 Hz), 6.2 (d, *J* = 7.0 Hz) ppm.

3.4. *P*-Chloro(n-butyl)diphenylphosphonium chloride **4.1-a** from **3.1-a**

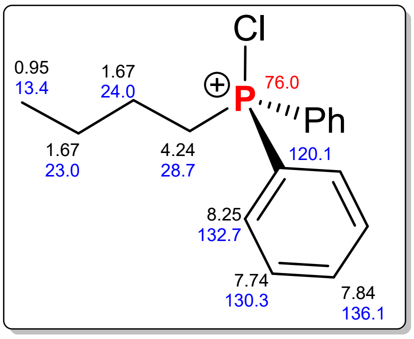

^{31}**P NMR** (162 MHz, CD_2Cl_2) δ 76.0 ppm. ^{1}**H NMR** (400 MHz, CD_2Cl_2) δ 8.46 – 8.10 (m, 4H), 7.89 – 7.80 (m, 2H), 7.81 – 7.63 (m, 4H), 4.69 – 3.67 (m, 2H), 1.93 – 1.44 (m, 4H), 0.95 (t, *J* = 7.1 Hz, 3H) ppm. ^{13}**C NMR** (101 MHz, CD_2Cl_2) δ 136.1 (d, *J* = 3.4 Hz), 132.7 (d, *J* = 13.2 Hz), 130.3 (d, *J* = 14.5 Hz), 120.1 (d, *J* = 87.5 Hz), 28.7 (d, *J* = 44.3 Hz), 24.0 (d, *J* = 5.9 Hz), 23.0 (d, *J* = 19.0 Hz), 13.4 (d, *J* = 1.2 Hz) ppm.

3.5. *P*-Chloro(di-*n*-butyl)phenylphosphonium chloride **4.2-a** from **3.2-a**

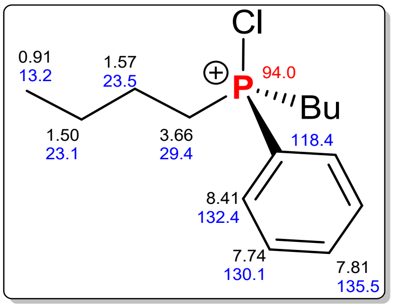

^{31}P NMR (162 MHz, CD_2Cl_2) δ 94.0 ppm. **^{1}H NMR** (400 MHz, CD_2Cl_2) δ 8.51 – 8.32 (m, 2H), 7.86 – 7.78 (m, 1H), 7.78 – 7.68 (m, 2H), 3.71 – 3.61 (m, 4H), 1.65 – 1.55 (m, 4H), 1.54 – 1.43 (m, 4H), 0.91 (t, *J* = 7.2 Hz, 6H) ppm.**^{13}C NMR** (101 MHz, CD_2Cl_2) δ 135.5 (d, *J* = 3.4 Hz), 132.4 (d, *J* = 12.5 Hz), 130.1 (d, *J* = 14.1 Hz), 118.4 (d, *J* = 81.4 Hz), 29.4 (d, *J* = 41.5 Hz), 23.5 (d, *J* = 6.5 Hz), 23.1 (d, *J* = 18.3 Hz), 13.2 (d, *J* = 1.0 Hz) ppm.

3.6. *P*-Chloro(*n*-butyl)(*n*-propyl)phenylphosphonium chloride **4.2-b** from **3.2-b**

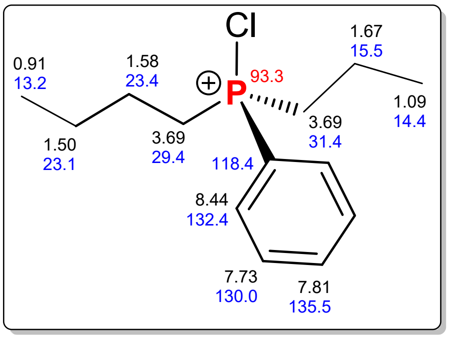

^{31}P NMR (162 MHz, CD_2Cl_2) δ 93.3 ppm. **^{1}H NMR** (400 MHz, CD_2Cl_2) δ 8.49 – 8.36 (m, 2H), 7.85 – 7.78 (m, 1H), 7.77 – 7.68 (m, 2H), 3.81 – 3.58 (m, 4H), 1.74 – 1.63 (m, 2H), 1.62 – 1.54 (m, 2H), 1.55 – 1.44 (m, 2H), 1.09 (td, *J* = 7.3 Hz, *J* = 1.7 Hz, 3H), 0.91 (t, *J* = 7.2 Hz, 3H) ppm.**^{13}C NMR** (101 MHz, CD_2Cl_2) δ 135.5 (d, *J* = 3.5 Hz), 132.4 (d, *J* = 12.6 Hz), 130.0 (d, *J* = 14.2 Hz), 118.4 (d, *J* = 81.3 Hz), 31.4 (d, *J* = 41.2 Hz), 29.4 (d, *J* = 41.2 Hz), 23.4 (d, *J* = 6.5 Hz), 23.1 (d, *J* = 18.3 Hz), 15.5 (d, *J* = 6.3 Hz), 14.4 (d, *J* = 19.0 Hz), 13.2 (d, *J* = 1.0 Hz) ppm.

3.7. *P*-Chlorotri-*n*-butylphosphonium chloride **4.3-a** from tri-*n*-butylphosphine oxide

This was prepared and characterized as described elsewhere.[1]

3.8. *P*-Chloro(*n*-butyl)(*n*-propyl)ethylphosphonium chloride **4.3-b** from **3.3-a**

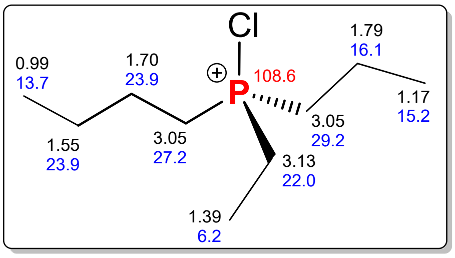

^{31}P NMR (162 MHz, CD_2Cl_2) δ 108.6 ppm. **^{1}H NMR** (400 MHz, CD_2Cl_2) δ 3.17 – 3.03 (overlapping m, 2H), 3.07 – 2.99 (overlapping m, 4H), 1.85 – 1.74 (overlapping m, 2H), 1.75 – 1.65 (overlapping m, 2H), 1.55 (apparent dq, *J* = 14.4 Hz, *J* = 7.2 Hz, 2H), 1.39 (dt, *J* = 22.6 Hz, *J* = 7.5 Hz, 3H), 1.17 (td, *J* = 7.3 Hz, *J* = 1.8 Hz, 3H), 0.99 (t, *J* = 7.3 Hz, 3H) ppm.**^{13}C NMR** (101 MHz, CD_2Cl_2) δ 29.2 (d, *J* = 40.8 Hz), 27.2 (d, *J* = 41.2 Hz), 23.9 (d, *J* = 6.4 Hz), 23.9 (d, *J* = 17.7 Hz), 22.0 (d, *J* = 42.7 Hz), 16.1 (d, *J* = 6.1 Hz), 15.2 (d, *J* = 18.6 Hz), 13.7, 6.2 ppm.

4. Quaternary Phosphonium Salts: Synthesis and Characterization Data

4.1. *n*-Butyltriphenylphosphonium chloride **2.1-a**

Walk from **3.0-a** via **4.0-a** (reaction **U1**).

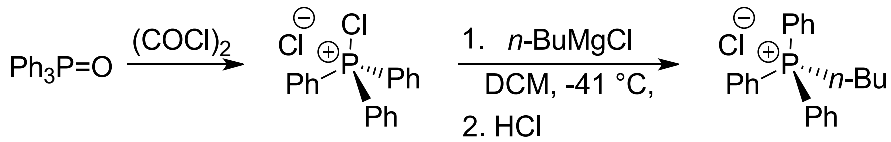

3.0-a **4.0-a** **2.1-a**

This was prepared in 86% spectroscopic yield and characterized as described elsewhere.[1]

Walk from **3.1-a** via **4.1-a** (reaction **U7**).

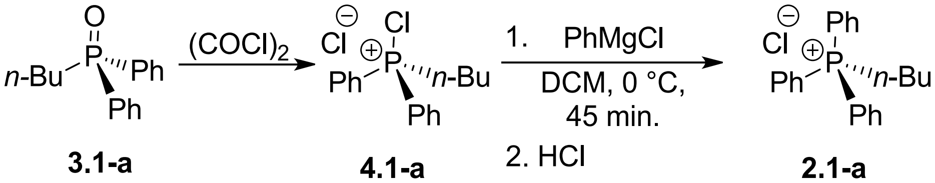

3.1-a **4.1-a** **2.1-a**

Using a 0.2 M DCM solution of **4.1-a** and phenylmagnesium chloride according to general procedure **B**. Observation: on addition of Grignard, the reaction mixture developed an orange colour, which faded upon quench. ^{31}P NMR (121 MHz, $CDCl_3$) δ 29.4 (unknown, 13%), 27.7 (unknown, 5%), 25.6 (unknown, 15%), 24.5 (**2.1-a**, 33%).

4.2. Methyltriphenylphosphonium chloride **2.1-b** from **4.0-a**

This was prepared (reaction **U1**) and characterized as described elsewhere.[1]

4.3. Ethyltriphenylphosphonium chloride **2.1-c** from **4.0-a**

This was prepared (reaction **U1**) and characterized as described elsewhere.[1]

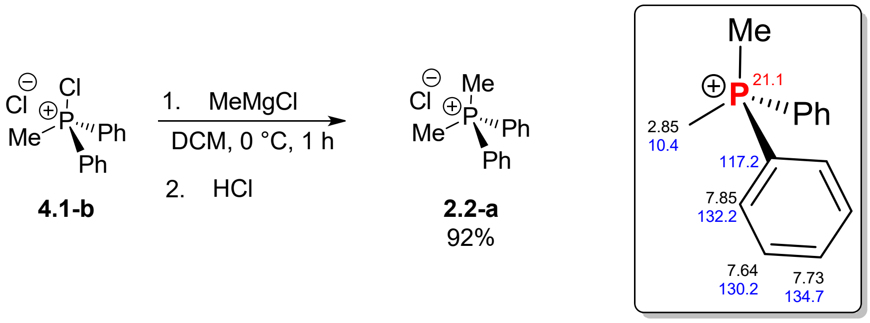

4.4. Dimethyldiphenylphosphonium chloride **2.2-a** from **4.1-b** (reaction **U2**)

Prepared in 92% spectroscopic yield following general procedure B at 0 °C, characterized without further purification. ^{31}P NMR (121 MHz, $CDCl_3$) δ 21.1 ppm; ^{1}H NMR (500 MHz, $CDCl_3$) δ 7.90 – 7.81 (m, 4H), 7.79 – 7.69 (m, 2H), 7.64 (td, *J* = 7.6 Hz, *J* = 3.2 Hz, 4H), 2.85 (d, *J* = 14.1 Hz, 6H) ppm; ^{13}C NMR (126 MHz, $CDCl_3$) δ 134.7 (d, *J* = 2.9 Hz), 132.2 (d, *J* = 10.6 Hz), 130.2 (d, *J* = 12.8 Hz), 117.2 (d, *J* = 87.1 Hz), 10.4 (d, *J* = 56.3 Hz) ppm. This phosphonium salt was previously described elsewhere.[2]

4.5. Ethyl(methyl)diphenylphosphonium chloride **2.2-b**

4.5.1. From **4.1-b** (reaction **U2**)

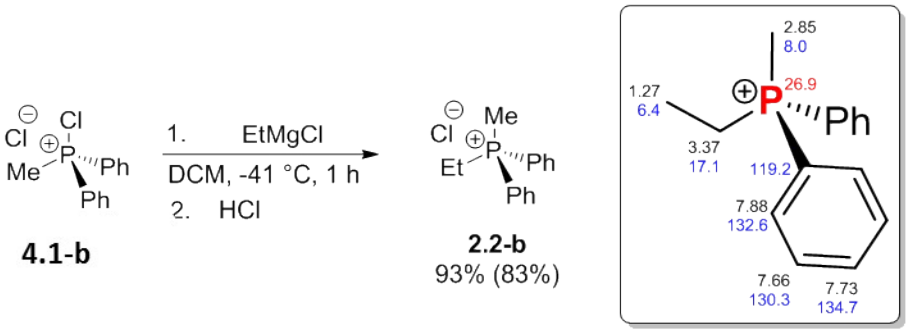

Prepared in 93% spectroscopic yield following general procedure B over 1 h at -41 °C. Recrystallization from chloroform/ethyl acetate afforded **2.2-b** as fine, off-white crystalline needles (659 mg, 83%): HRMS (ES$^+$) m/z: calculated for $C_{15}H_{18}P^+$ 229.1146, found 229.1141. ^{31}P NMR (162 MHz, $CDCl_3$) δ 26.9 ppm; ^{1}H NMR (400 MHz, $CDCl_3$) δ 7.92 – 7.82 (m, 4H), 7.80 – 7.70 (m, 2H), 7.69 – 7.59 (m, 4H), 3.37 (dq, *J* = 13.2 Hz, *J* = 7.5 Hz, 2H), 2.85 (d, *J* = 13.7 Hz, 3H), 1.27 (dt, *J* = 20.6, 7.5 Hz, 3H) ppm; ^{13}C NMR (101 MHz, $CDCl_3$) δ 134.7 (d, *J* = 3.0 Hz), 132.6 (d, *J* = 10.0 Hz), 130.3 (d, *J* = 12.4 Hz), 119.2 (d, *J* = 84.6 Hz), 17.1 (d, *J* = 52.1 Hz), 8.0 (d, *J* = 55.0 Hz), 6.4 (d, *J* = 5.2 Hz) ppm.

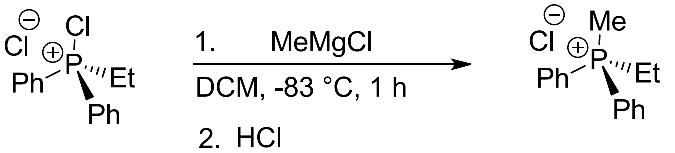

4.1-c

2.2-b
89% (80%)

4.5.2. From **4.1-c** (reaction **U2**)

Prepared in 89% spectroscopic yield following general procedure B over 1 h at -83 °C. Recrystallization from chloroform/ethyl acetate gave 635 mg, 80% of **2.2-b**.

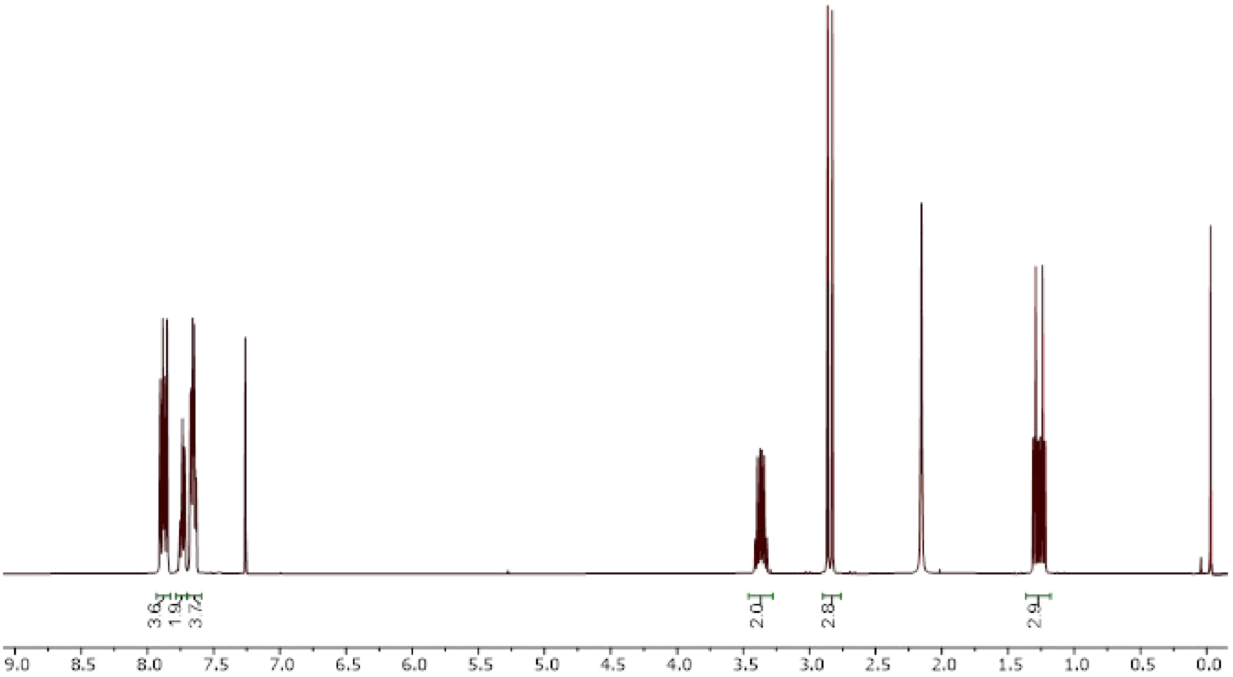

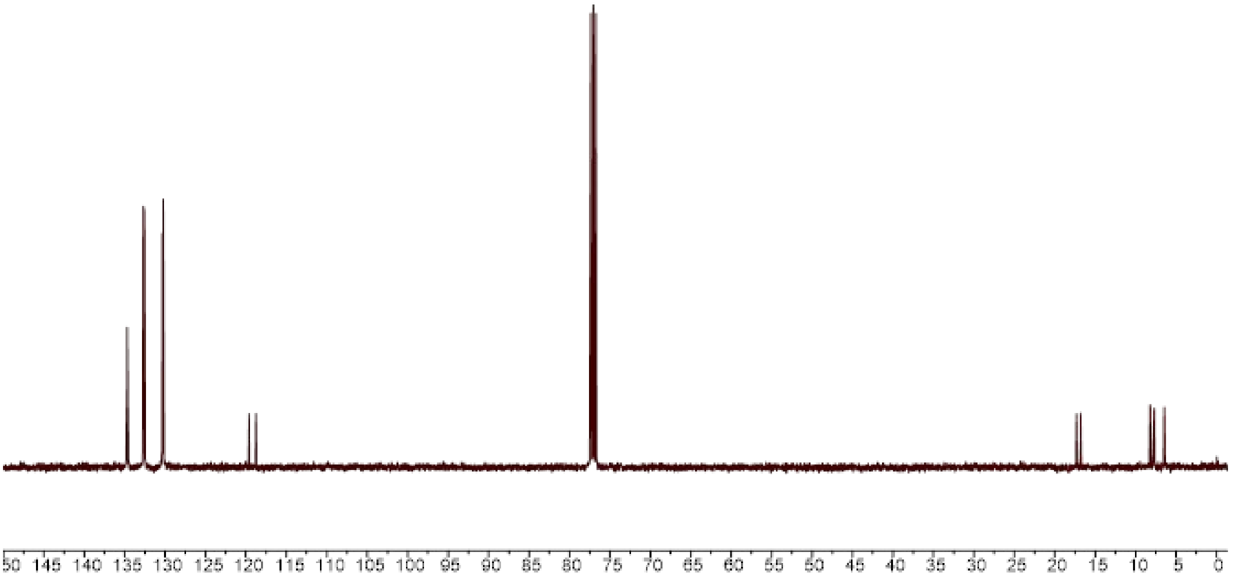

Figure S1. Top: ^{1}H NMR spectrum of **2.2-b**; bottom: ^{13}C NMR spectrum of **2.2-b.**

4.6. Ethyldiphenyl(*n*-propyl)phosphonium chloride **2.2-c** from **4.1-c** (reaction **U2**)

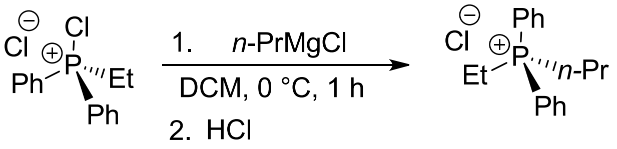

4.1-c

2.2-c
88%

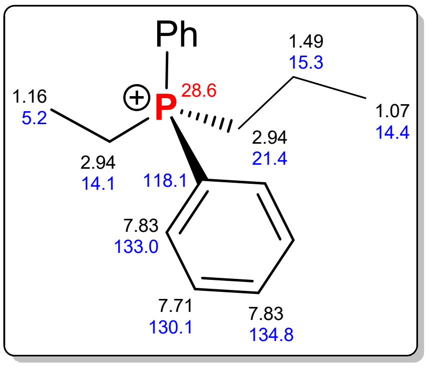

Prepared in 88% spectroscopic yield following general procedure B over 1 h at 0 °C and characterized without further purification: HRMS (ES^{+}) m/z: calculated for $C_{17}H_{22}P^{+}$ 257.1459, found 257.1455. ^{31}P NMR (162 MHz, CD_3CN) δ 28.6 ppm; ^{1}H NMR (400 MHz, CD_3CN) δ 7.86 – 7.76 (m, 6H), 7.71 (apparent ddd, *J* = 8.1, 7.0, 3.3 Hz, 4H), 3.05 – 2.83 (m, 4H), 1.59 – 1.42 (m, 2H), 1.16 (dt, *J* = 19.7, 7.5 Hz, 1H), 1.07 (td, *J* = 7.3, 1.6 Hz, 3H) ppm; ^{13}C NMR (101 MHz, CD_3CN) δ 134.8 (d, *J* = 3.0 Hz), 133.0 (d, *J* = 9.5 Hz), 130.1 (d, *J* = 12.4 Hz), 118.1 (d, *J* = 83.3 Hz), 21.4 (d, *J* = 49.7 Hz), 15.3 (d, *J* = 3.8 Hz), 14.4 (d, *J* = 17.1 Hz), 14.1 (d, *J* = 51.5 Hz), 5.2 (d, *J* = 4.9 Hz) ppm.

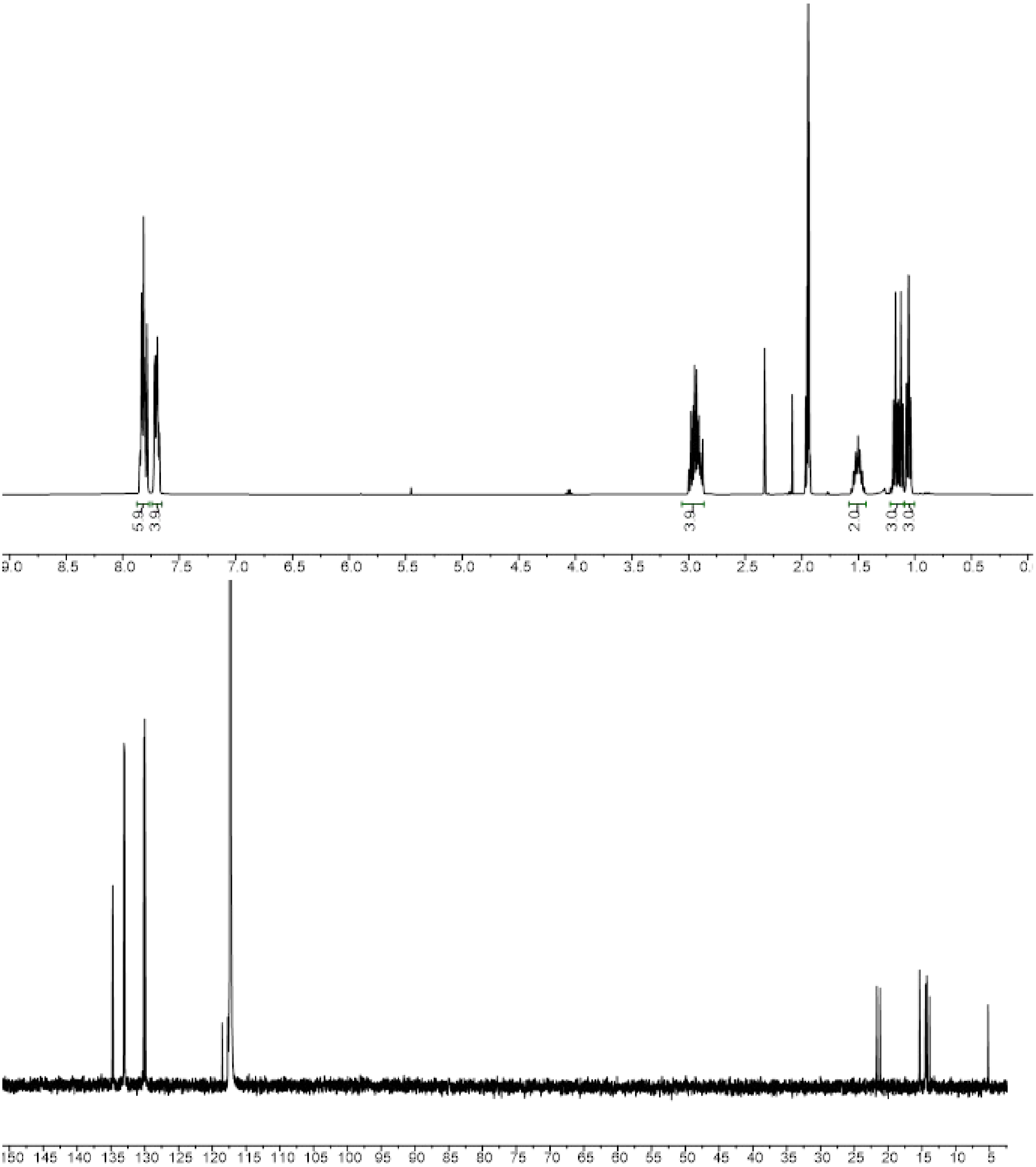

Figure S2. Top: ^{1}H NMR spectrum of **2.2-c**; bottom: ^{13}C NMR spectrum of **2.2-c.**

4.7. *n*-Butylmethyl(diphenyl)phosphonium chloride **2.2-d** from **4.1-b** (reaction **U2**)

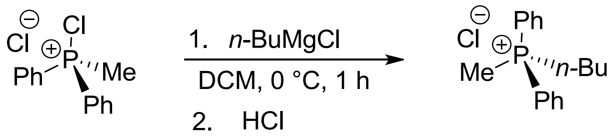

4.1-b **2.2-d** 92%

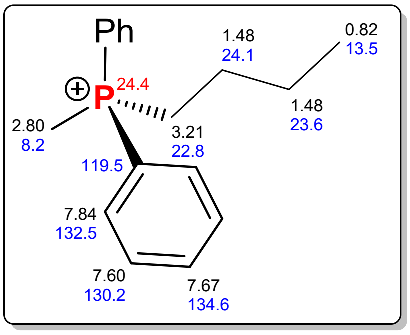

Prepared in 92% spectroscopic yield following general procedure B over 1 h at 0 °C and characterized without further purification: HRMS (ES$^+$) m/z: calculated for $C_{17}H_{22}P^+$ 257.1459, found 257.1470. ^{31}P NMR (162 MHz, $CDCl_3$) δ 24.4 ppm; ^{1}H NMR (400 MHz, $CDCl_3$) δ 7.92 – 7.78 (m, 4H), 7.74 – 7.64 (m, 2H), 7.65 – 7.53 (m, 4H), 3.30 – 3.10 (m, 2H), 2.80 (d, *J* = 13.7 Hz, 3H), 1.60 – 1.35 (m, 4H), 0.82 (t, *J* = 7.1 Hz, 3H) ppm; ^{13}C NMR (101 MHz, $CDCl_3$) δ 134.6 (d, *J* = 3.0 Hz), 132.5 (d, *J* = 10.0 Hz), 130.2 (d, *J* = 12.5 Hz), 119.5 (d, *J* = 84.6 Hz), 24.1 (d, *J* = 4.3 Hz), 23.6 (d, *J* = 16.8 Hz), 22.8 (d, *J* = 50.9 Hz), 13.5, 8.2 (d, *J* = 55.2 Hz) ppm.

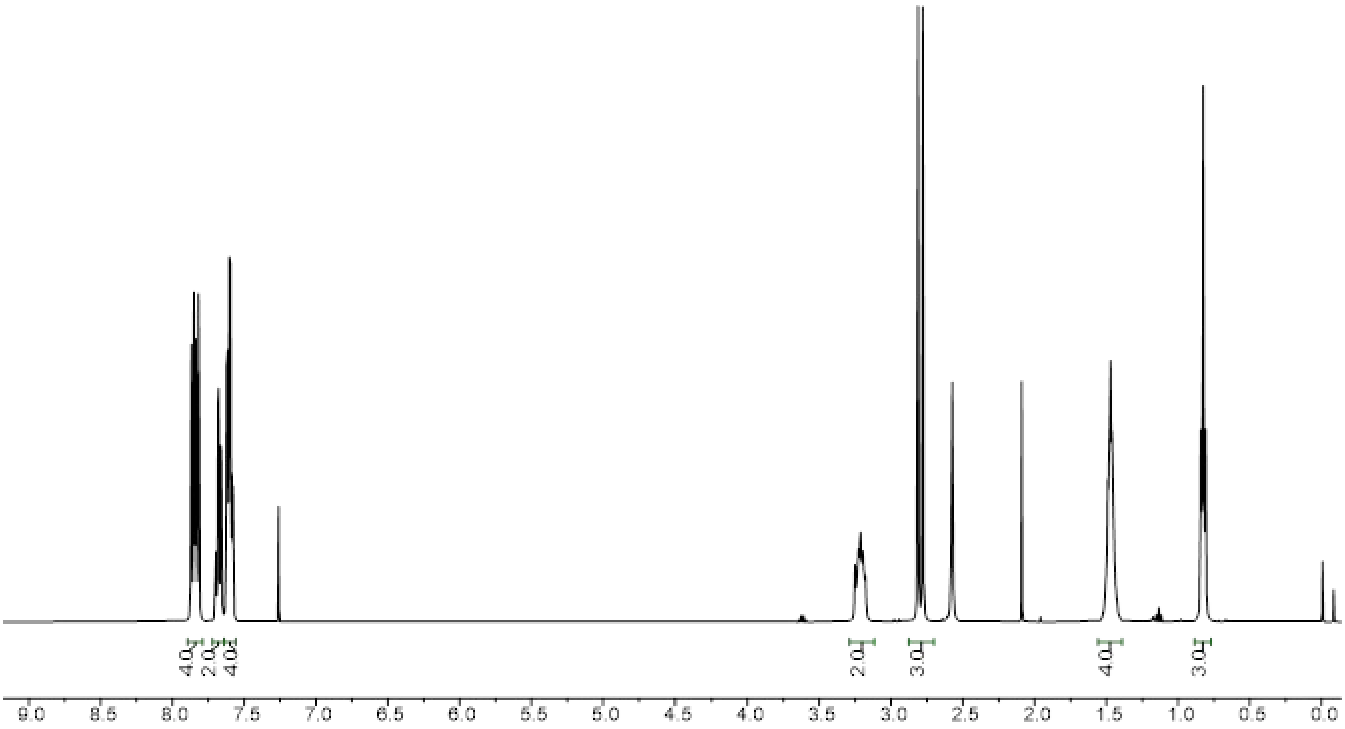

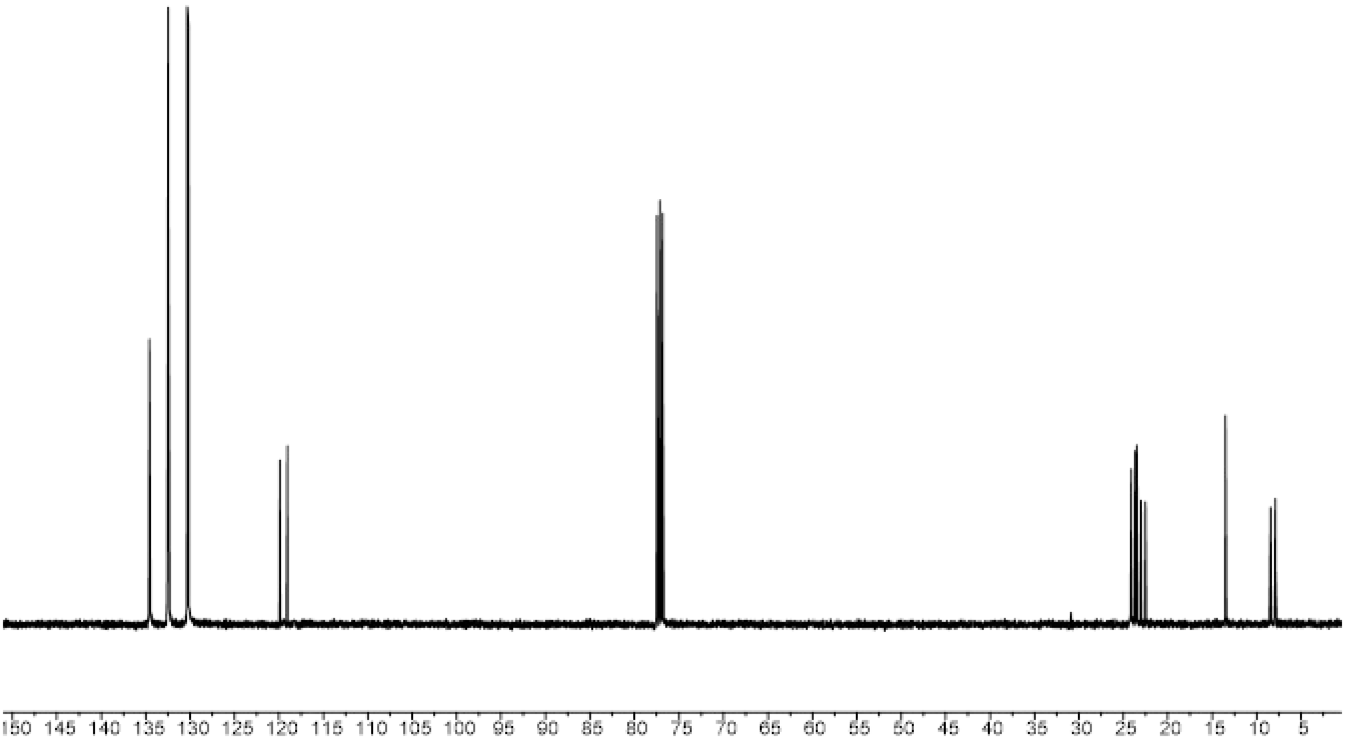

Figure S3. Top: ^{1}H NMR spectrum of **2.2-d**; bottom: ^{13}C NMR spectrum of **2.2-d.**

4.8. Diethyl(diphenyl)phosphonium chloride **2.2-e** from **4.1-c** (reaction **U2**)

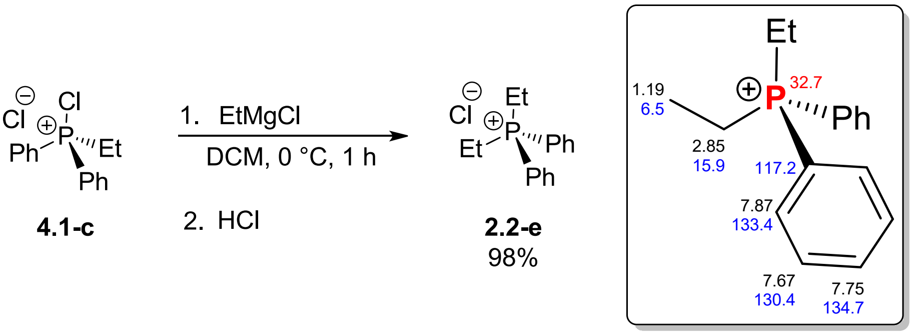

Prepared in 98% spectroscopic yield following general procedure B over 1 h at 0 °C and characterized without further purification. ^{31}P NMR (162 MHz, $CDCl_3$) δ 32.7 ppm; ^{1}H NMR (500 MHz, $CDCl_3$) δ 7.95 – 7.80 (m, 4H), 7.78 – 7.70 (m, 2H), 7.70 – 7.58 (m, 4H), 2.85 (dq, J = 12.8, 7.5 Hz, 4H), 1.19 (dt, J = 19.6, 7.5 Hz, 6H) ppm; ^{13}C NMR (101 MHz, $CDCl_3$) δ 134.7 (d, J = 2.99 Hz), 133.4 (d, J = 9.2 Hz), 130.4 (d, J = 11.9 Hz), 117.2 (d, J = 82.0 Hz), 15.9 (d, J = 50.5 Hz), 6.5 (d, J = 5.3 Hz) ppm. This phosphonium salt was previously described elsewhere.[3]

4.9. Benzyl(ethyl)diphenylphosphonium chloride **2.2-f** from **4.1-c** (reaction **U2**)

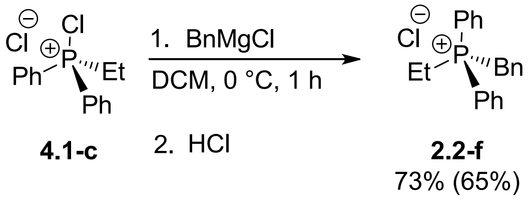

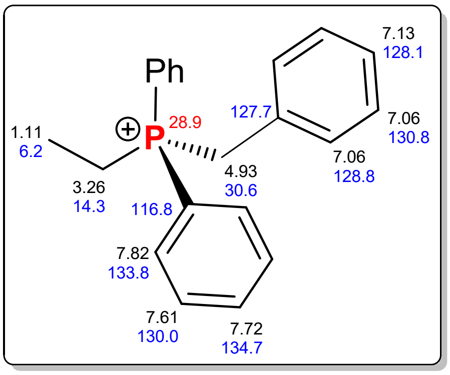

Prepared in 73% spectroscopic yield following general procedure B over 1 h at 0 °C. Recrystallization from chloroform/ethyl acetate afforded **2.2-f** as white crystals (440 mg, 65%): HRMS (ES$^+$) m/z: calculated for $C_{21}H_{22}P^+$ 305.1459, found 305.1474. ^{31}P NMR (162 MHz, $CDCl_3$) δ 28.9 ppm; ^{1}H NMR (400 MHz, $CDCl_3$) δ 7.88 – 7.78 (m, 4H), 7.76 – 7.68 (m, 2H), 7.61 (td, *J* = 7.6, 3.2 Hz, 4H), 7.17 – 7.11 (m, 1H), 7.11 – 7.01 (m, 4H), 4.93 (d, *J* = 15.0 Hz, 2H), 3.26 (dq, *J* = 14.8, 7.3 Hz, 2H), 1.11 (dt, *J* = 19.8, 7.4 Hz, 3H) ppm; ^{13}C NMR (101 MHz, $CDCl_3$) δ 134.7 (d, *J* = 3.0 Hz), 133.8 (d, *J* = 9.0 Hz), 130. (d, *J* = 5.6 Hz), 130.0 (d, *J* = 12.1 Hz), 128.8 (d, *J* = 3.4 Hz), 128.1 (d, *J* = 3.8 Hz), 127.7 (d, *J* = 8.8 Hz), 116.8 (d, *J* = 82.0 Hz), 30.6 (d, *J* = 46.0 Hz), 14.3 (d, *J* = 50.8 Hz), 6.2 (d, *J* = 5.4 Hz) ppm.

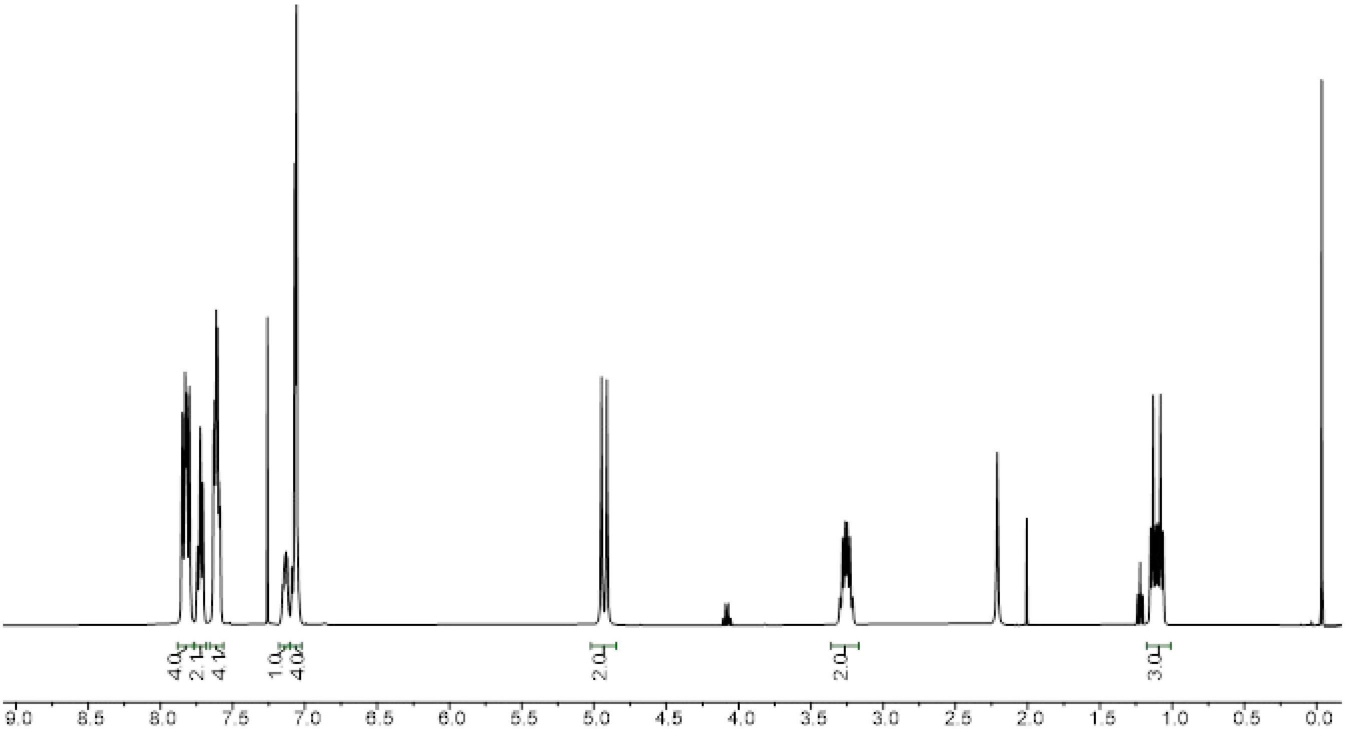

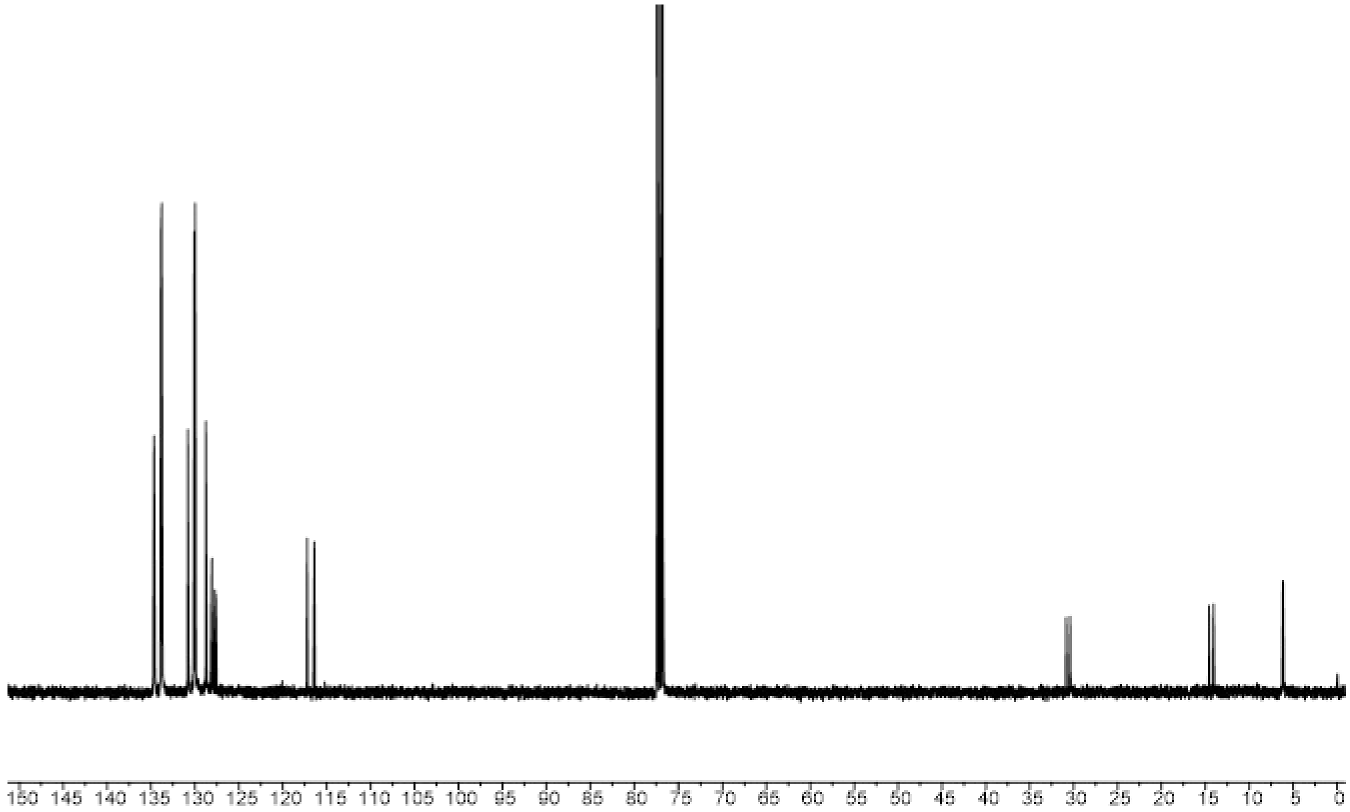

Figure S4. Top: ^{1}H NMR spectrum of **2.2-f**; bottom: ^{13}C NMR spectrum of **2.2-f.**

4.10. Di-*n*-butyldiphenylphosphonium chloride **2.2-g**

2.2-g from **4.1-a** (reaction **U2**)

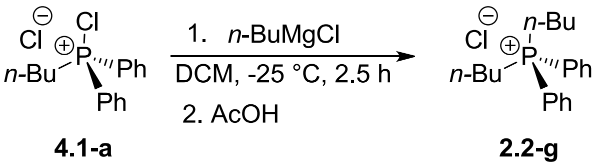

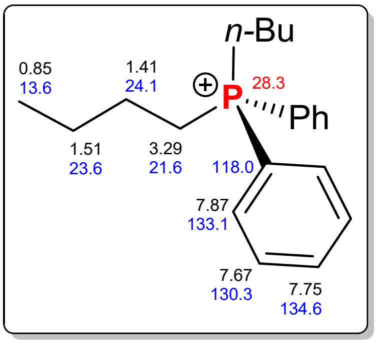

Prepared following general procedure B over 2.5 h at -25 °C and using AcOH to quench the reaction mixture. Recrystallization from chloroform/ethyl acetate afforded **2.2-g** as white crystals (2.80 g, 95%): HRMS (ES$^+$) calculated for $C_{20}H_{28}P^+$ = 299.1929, found 299.1918. ^{31}P NMR (162 MHz, $CDCl_3$) δ 28.3 ppm; ^{1}H NMR (400 MHz, $CDCl_3$) δ 7.91 – 7.83 (m, 4H), 7.78 – 7.71 (m, 2H), 7.71 – 7.62 (m, 4H), 3.36 – 3.22 (m, 4H), 1.57 – 1.45 (m, 4H), 1.46 – 1.33 (m, 4H), 0.85 (t, *J* = 7.1 Hz, 6H) ppm; ^{13}C NMR (101 MHz, $CDCl_3$) δ 134.6 (d, *J* = 3.0 Hz), 133.1 (d, *J* = 9.3 Hz), 130.3 (d, *J* = 12.1 Hz), 118.0 (d, *J* = 82.0 Hz), 24.1 (d, *J* = 4.6 Hz), 23.6 (d, *J* = 16.4 Hz), 21.6 (d, *J* = 49.1 Hz), 13.6 ppm. This phosphonium salt was previously described elsewhere.[4]

When this material was prepared following general procedure B over 1 h at 0 °C, a slightly lower yield of 90% was obtained.

2.2-g from **3.2-a** via **4.2-a** (reaction **U6**)

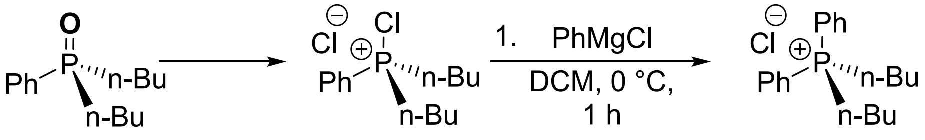

Prepared following general procedure B over 1 h using a 0.2 M DCM solution of **4.2-a** and phenylmagnesium chloride (2.0 M in THF, 1.00 mL, 2.00 mmol). ^{31}P NMR (121 MHz, $CDCl_3$) analysis indicated δ 51.3 (**3.2-a**·HCl, 18%), 34.6 (d, *J* = 6.2 Hz, unknown, 3%), 34.1 (unknown, 2%), 32.5 (d, *J* = 6.2 Hz, unknown, 3%), 30.2 (unknown, 2%), 28.3 (**2.2-g**, 66%).

4.11. *n*-Butyl(*iso*-butyl)diphenylphosphonium chloride **2.2-h** from **4.1-a** (reaction **U2**)

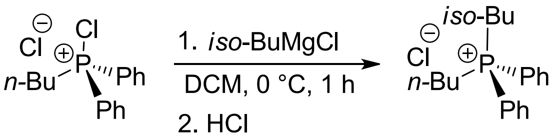

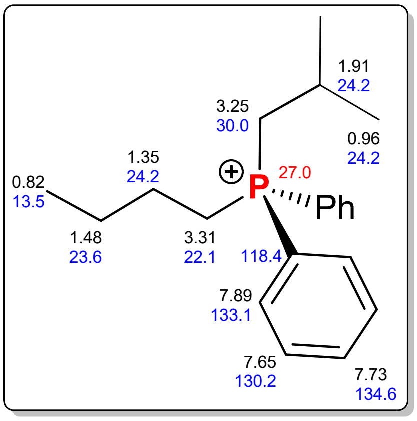

Prepared in 95% spectroscopic yield following general procedure B over 1 h at 0 °C. After column chromatography (MeCN:2-propanol 95:5 to 50:50), **2.2-h** was obtained as clear oil (284 mg, 85%): HRMS (ES$^+$) calculated for $C_{20}H_{28}P^+$ = 299.1929, found 299.1920. ^{31}P NMR (162 MHz, $CDCl_3$) δ 27.0 ppm; ^{1}H NMR (400 MHz, $CDCl_3$) δ 7.95 – 7.83 (m, 4H), 7.77 – 7.69 (m, 2H), 7.69 – 7.58 (m, 4H), 3.35 – 3.29 (m, 2H), 3.25 (dd, *J* = 13.0, 6.5 Hz, 2H), 1.97 – 1.82 (m, 1H), 1.48 (apparent p, *J* = 8.1 Hz, 2H), 1.41 – 1.28 (m, 2H), 0.96 (dd, *J* = 6.7, 1.0 Hz, 6H), 0.82 (t, *J* = 7.2 Hz, 3H) ppm; ^{13}C NMR (101 MHz, $CDCl_3$) δ 134.6 (d, *J* = 3.0 Hz), 133.1 (d, *J* = 9.3 Hz), 130.2 (d, *J* = 12.0 Hz), 118.4 (d, *J* = 81.6 Hz), 30.0 (d, *J* = 47.0 Hz), 24.2 (d, *J* = 4.3 Hz), 24.2 (d, *J* = 3.0 Hz), 23.6 (d, *J* = 16.2 Hz), 22.1 (d, *J* = 48.9 Hz), 13.5 (d, *J* = 0.9 Hz) ppm.

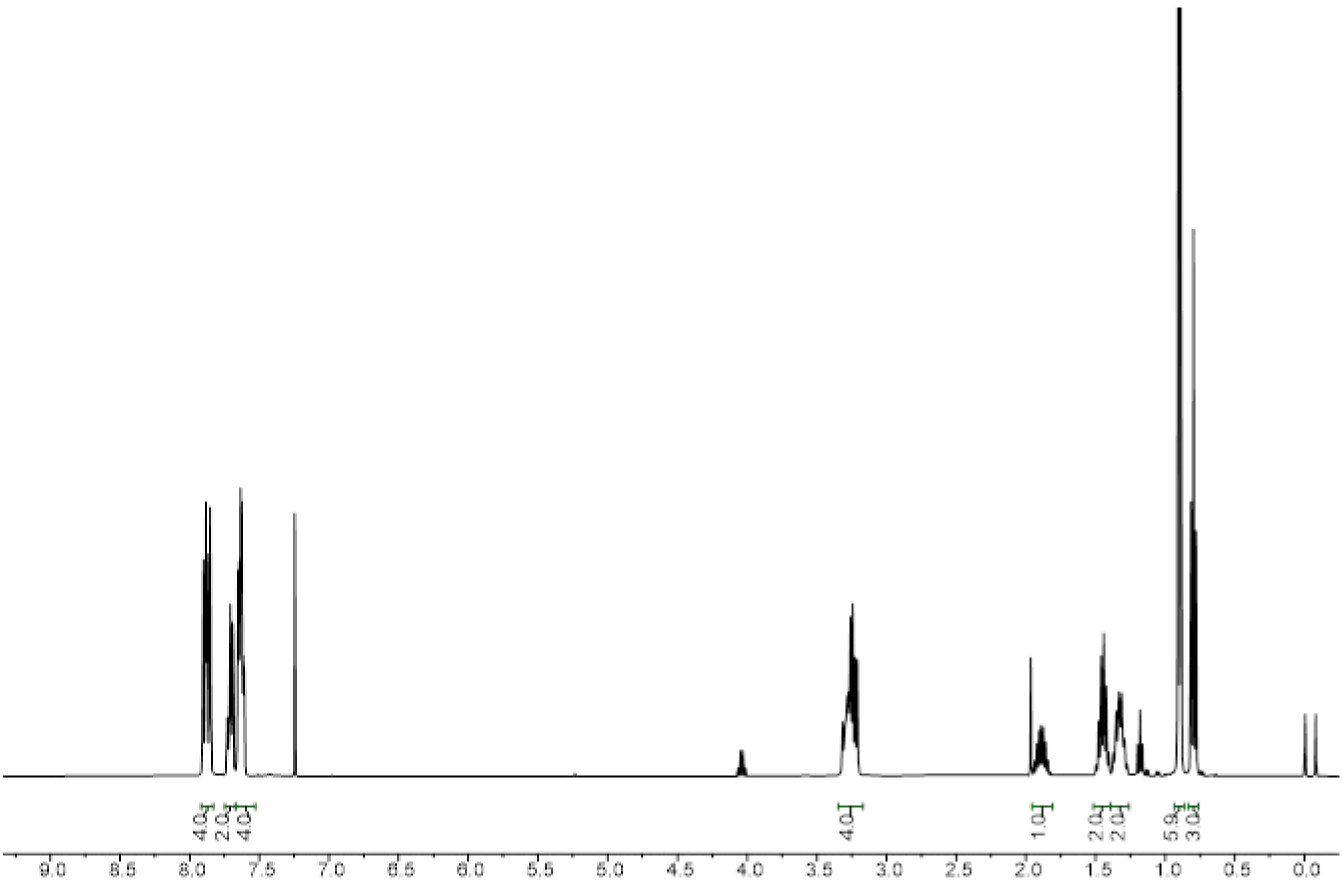

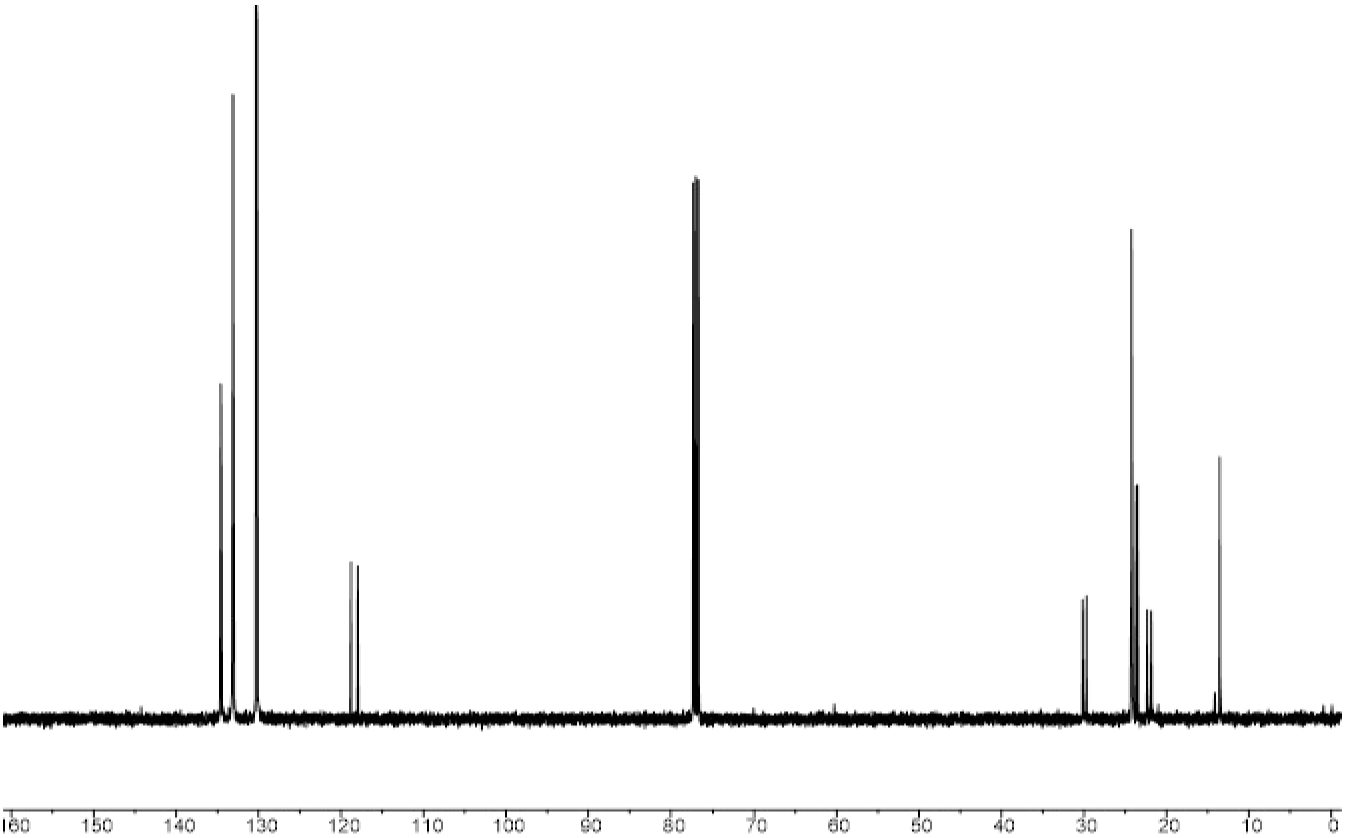

Figure S5. Top: ^{1}H NMR spectrum of **2.2-h**; bottom: ^{13}C NMR spectrum of **2.2-h**.

4.12. *n*-Butyl(diphenyl)(*n*-propyl)phosphonium chloride **2.2-i** from **4.1-a** (reaction **U2**)

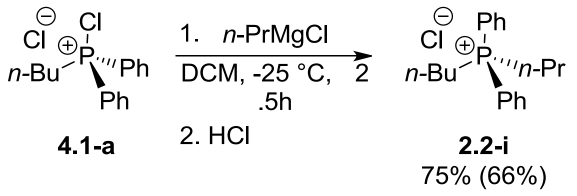

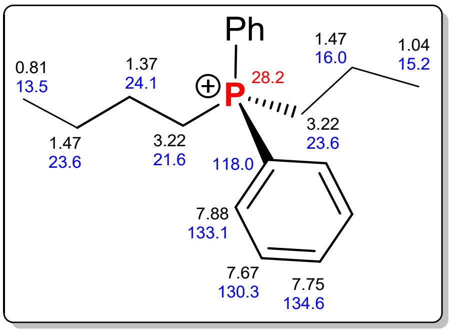

Prepared in 75% yield following general procedure B over 2.5 h at -25 °C. Recrystallization from MeCN/ethyl acetate afforded **2.2-i** as fine crystalline material (2.901 g, 66%): HRMS (ES$^+$) calculated for $C_{19}H_{26}P^+$ = 285.1772, found 285.1760. ^{31}P NMR (121 MHz, $CDCl_3$) δ 28.2 ppm; ^{1}H NMR (500 MHz, $CDCl_3$) δ 7.89 – 7.80 (m, 4H), 7.75 (td, *J* = 7.1, 1.4 Hz, 2H), 7.67 – 7.57 (m, 4H), 3.30 – 3.15 (m, 4H), 1.54 – 1.40 (m, 4H), 1.42 – 1.32 (m, 2H), 1.04 (td, *J* = 7.3, 1.6 Hz, 3H), 0.81 (t, *J* = 7.2 Hz, 3H) ppm; ^{13}C NMR (126 MHz, $CDCl_3$) δ 134.6 (d, *J* = 2.9 Hz), 133.1 (d, *J* = 9.2 Hz), 130.3 (d, *J* = 12.2 Hz), 118.0 (d, *J* = 82.1 Hz), 24.1 (d, *J* = 4.6 Hz), 23.6 (d, *J* = 16.1 Hz), 23.6 (d, *J* = 49.1 Hz), 21.6 (d, *J* = 49.1 Hz), 16.0 (d, *J* = 4.3 Hz), 15.2 (d, *J* = 16.8 Hz), 13.5 ppm.

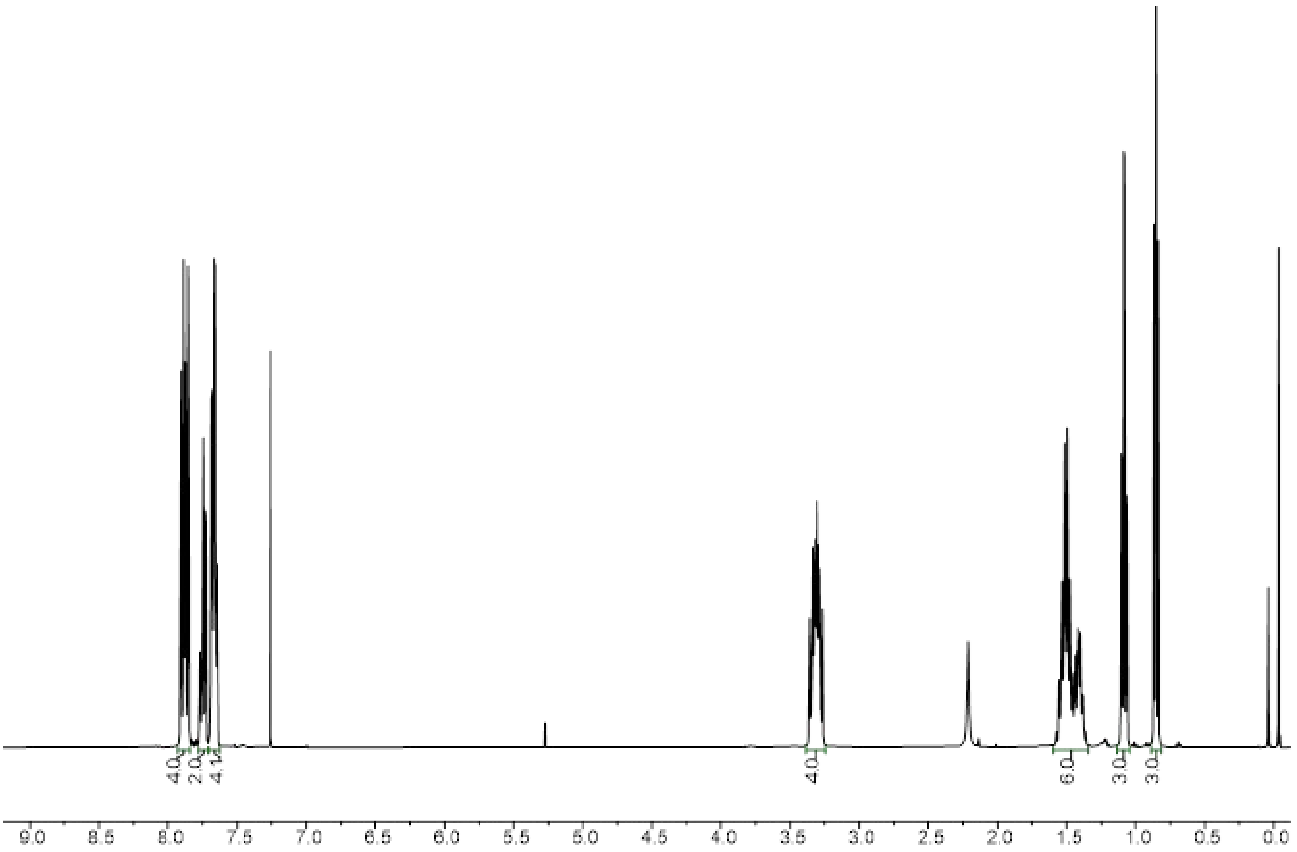

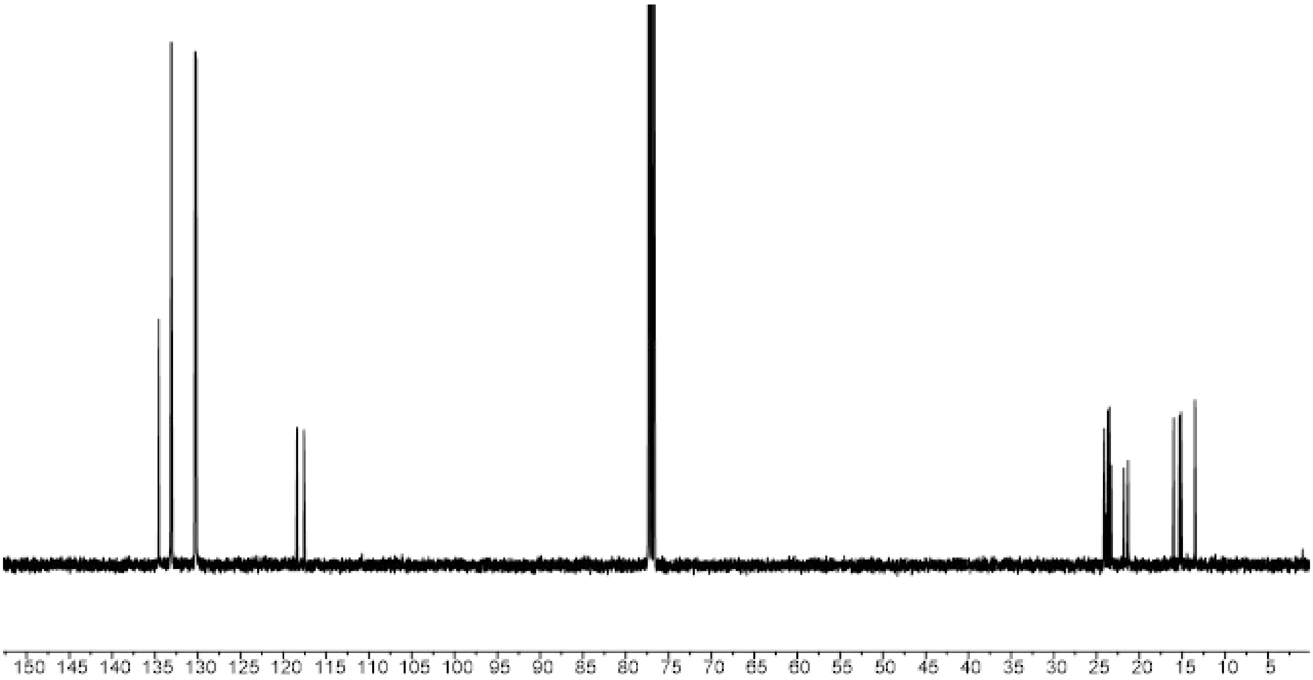

Figure S6. Top: ^{1}H NMR spectrum of **2.2-i**; bottom: ^{13}C NMR spectrum of **2.2-i.**

4.13. Vinyltri-*n*-butylphosphonium chloride **2.3-e** from **4.3-a** (reaction **U4**)

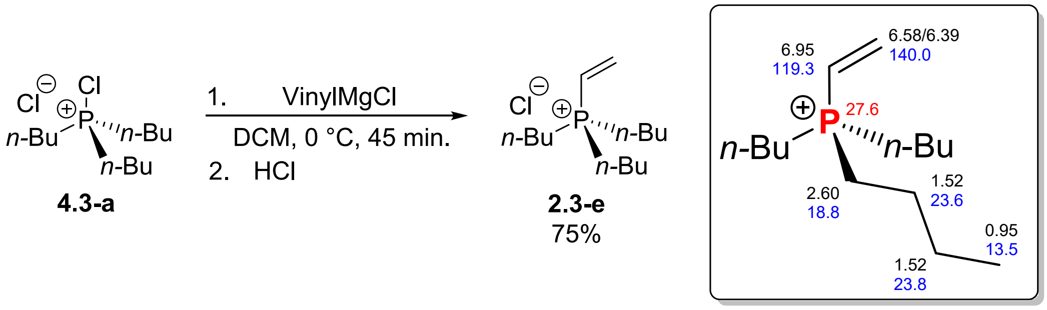

Prepared in 75% spectroscopic yield following general procedure B over 45 min. at 0 °C and characterized without further separation from tri-*n*-butylphosphine oxide. ^{31}P NMR (121 MHz, $CDCl_3$) δ 27.6 ppm; ^{1}H NMR (400 MHz, $CDCl_3$) δ 6.95 (ddd, *J* = 20.3 Hz, *J* = 18.9 Hz, *J* = 13.0 Hz, 1H), 6.58 (dd, *J* = 44.1 Hz, *J* = 13.0 Hz, 1H), 6.39 (dd, *J* = 23, 18.9 Hz, 1H), 2.74 – 2.48 (m, 6H), 1.64 – 1.39 (m, 12H), 0.95 (t, *J* = 7.1 Hz, 9H) ppm; ^{13}C NMR (101 MHz, $CDCl_3$) δ 140.0, 119.3 (d, *J* = 72.5 Hz), 23.8 (d, *J* = 15.6 Hz), 23.6 (d, *J* = 4.6 Hz), 18.8 (d, *J* = 49.3 Hz), 13.5 ppm. The chemical shifts and coupling constants are in perfect agreement with the data published elsewhere.[5]

4.14. Phenyl(tri-*n*-butyl)phosphonium chloride **2.3-a**

Walk from **3.2-a** via **4.2-a** (reaction **U3**).

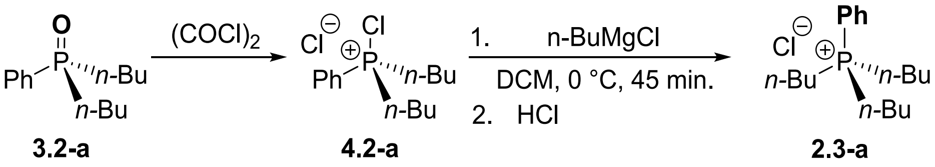

This was prepared in 89% spectroscopic yield and characterized as described elsewhere. [1]

Walk from **3.3-b** via **4.3-a** (reaction **U5**).

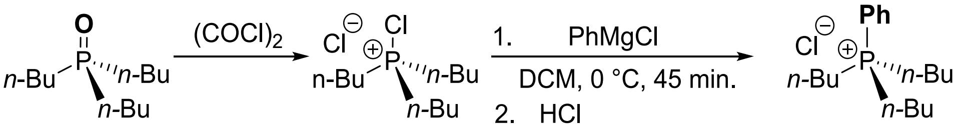

3.3-b **4.3-a** **2.3-a**

This was prepared in 95% spectroscopic yield and characterized as described elsewhere.[1]

4.15. Di-*n*-butyl(*iso*-butyl)phenylphosphonium chloride **2.3-b** from **4.2-a** (reaction **U3**)

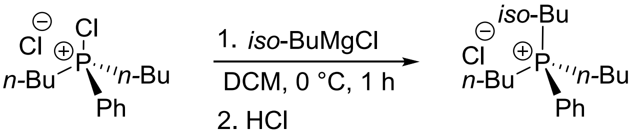

4.2-a **2.3-b** 91% (84%)

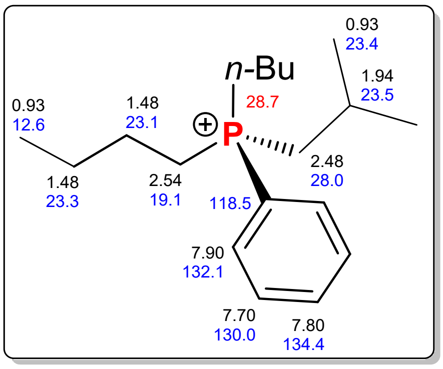

Prepared in 91% yield following general procedure B over 1 h at 0 °C and after column chromatography (95:5 MeCN/2-propanol to 50:50 MeCN/2-propanol) isolated as clear oil (138 mg, 84%): HRMS (ES$^+$) calculated for $C_{18}H_{32}P^+$ = 279.2242, found 279.2230. ^{31}P NMR (121 MHz, CD_3CN) δ 28.7 ppm; ^{1}H NMR (400 MHz, CD_3CN) δ 7.95 – 7.85 (m, 2H), 7.83 – 7.76 (m, 1H), 7.74 – 7.64 (m, 2H), 2.58 – 2.52 (m, 4H), 2.48 (dd, *J* = 13.0, 6.6 Hz, 2H), 1.94 (m, 1H), 1.57 – 1.39 (m, 8H), 0.96 – 0.89 (m, 12 H) ppm; ^{13}C NMR (101 MHz, CD_3CN) δ 134.4 (d, *J* = 3.1 Hz), 132.1 (d, *J* = 8.7 Hz), 130.0 (d, *J* = 11.9 Hz), 118.5 (d, *J* = 79.4 Hz), 28.0 (d, *J* = 46.9 Hz), 23.5 (d, *J* = 4.6 Hz), 23.4 (d, *J* = 1.5 Hz), 23.3 (d, *J* = 6 Hz), 23.1 (d, *J* = 4.0 Hz), 19.1 (d, *J* = 49.0 Hz), 12.6 ppm.

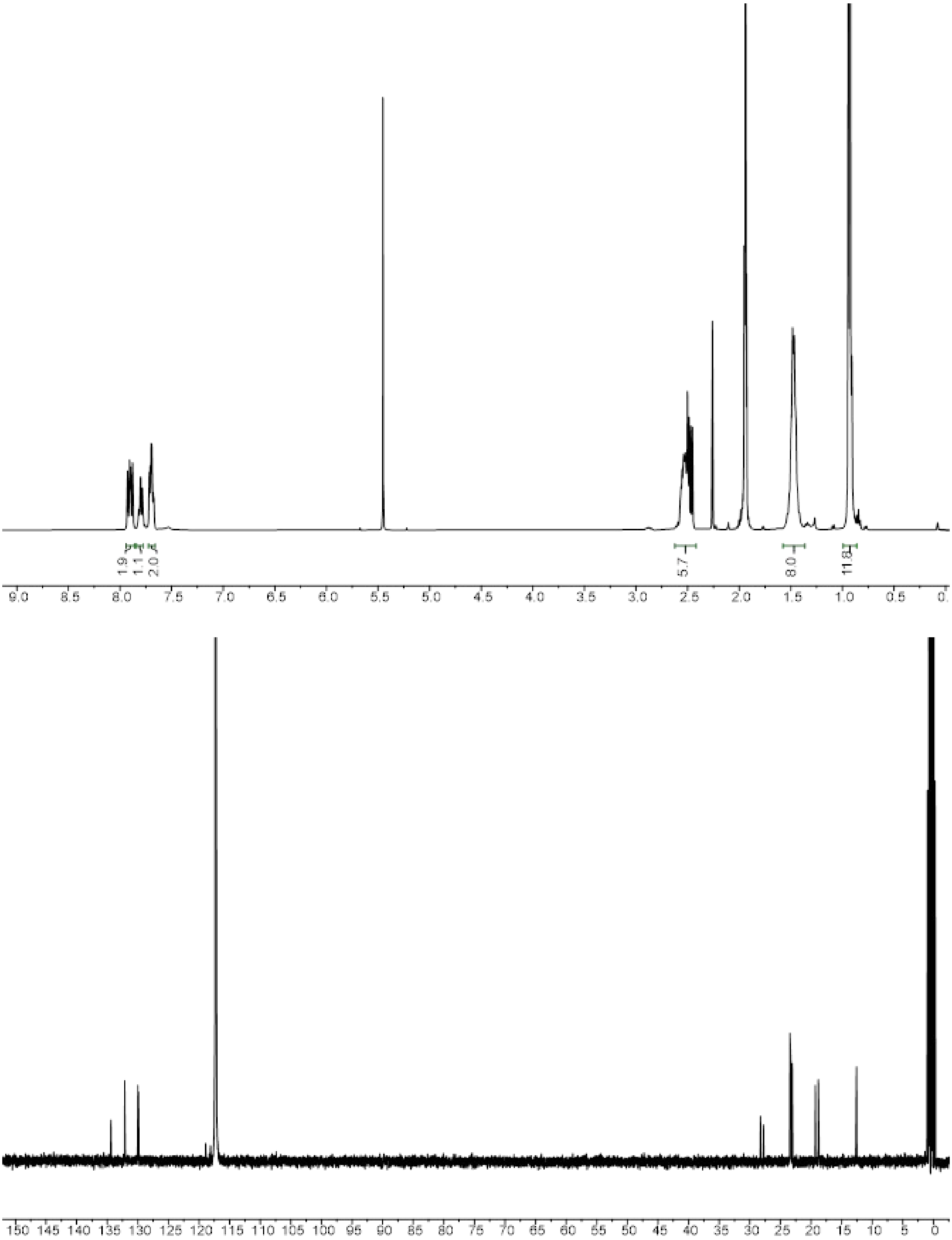

Figure S7. Top: ^{1}H NMR spectrum of **2.3-b**; bottom: ^{13}C NMR spectrum of **2.3-b.**

4.16. Di-*n*-butyl(phenyl)(*iso*-propyl)phosphonium chloride **2.3-c** from **4.2-a** (reaction **U3**)

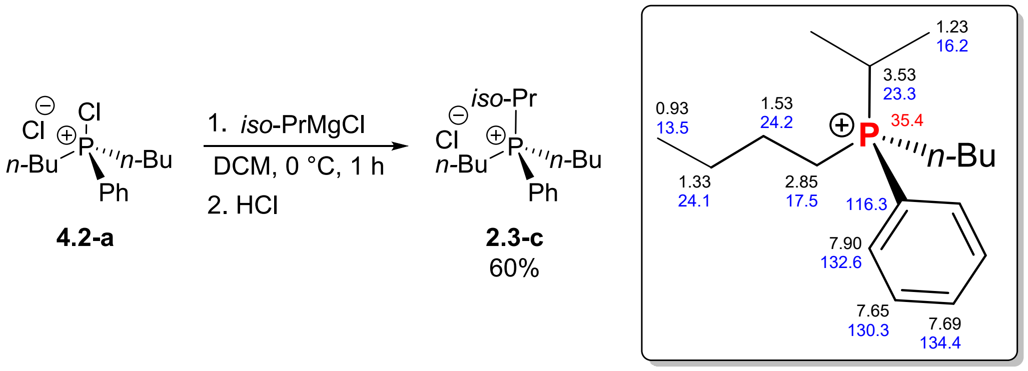

Prepared in 60% spectroscopic yield following general procedure B over 1 h at 0 °C and was characterized without further purification. The following signals were identified and assigned to **2.3-c**: ^{31}P NMR (121 MHz, $CDCl_3$) δ 35.4 ppm; ^{1}H NMR (400 MHz, $CDCl_3$) δ 7.95 – 7.85 (m, 2H), 7.71 – 7.66 (m, 1H), 7.70 – 7.62 (m, 2H), 3.62 – 3.43 (m, 1H), 3.03 – 2.65 (m, 4H), 1.61 – 1.42 (m, 4H), 1.42 – 1.29 (m, 4H), 1.32 – 1.15 (m, 6H), 1.02 – 0.87 (t, *J* = 7.1 Hz, 6H) ppm; ^{13}C NMR (101 MHz, $CDCl_3$) δ 134.4 (d, *J* = 2.8 Hz), 132.6 (d, *J* = 8.0 Hz), 130.3 (d, *J* = 11.3 Hz), 116.3 (d, *J* = 76.4 Hz), 24.2 (d, *J* = 4.6 Hz), 24.1 (d, *J* = 15.2 Hz), 23.3 (d, *J* = 46.2 Hz), 17.5 (d, *J* = 47.0 Hz), 16.2 (d, *J* = 2.7 Hz), 13.5 (d, *J* = 2.2 Hz) ppm.

4.17. *n*-Butyl(ethyl)(phenyl)(propyl)phosphonium chloride **2.3-d** from **4.2-b** (reaction **U3**)

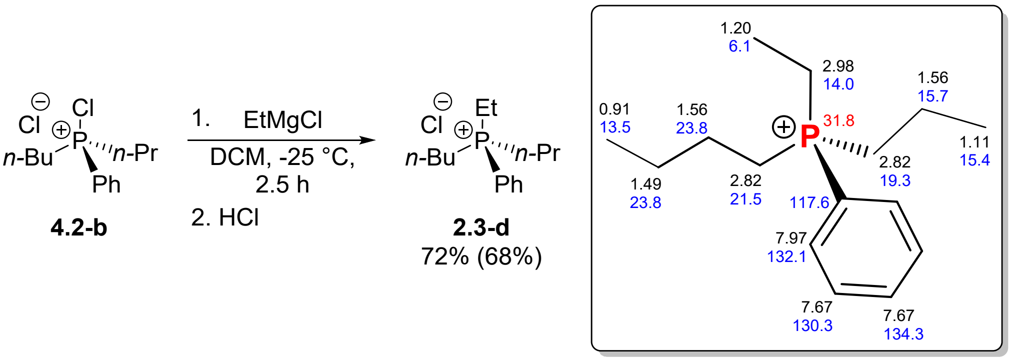

Prepared in 72% yield following general procedure B over 2.5 h at -25 °C. After column chromatography (95:5 MeCN/2-propanol to 50:50 MeCN/2-propanol) **2.3-d** was obtained as clear oil (927 mg, 68%): HRMS (ES$^+$) calculated for $C_{15}H_{26}P^+$ = 237.1772, found 237.1762. ^{31}P NMR (162 MHz, $CDCl_3$) δ 31.8 ppm; ^{1}H NMR (400 MHz, $CDCl_3$) δ 7.99 – 7.90 (m, 2H), 7.73 – 7.61 (m, 3H), 2.98 (dq, *J* = 12.9, 7.6 Hz, 2H), 2.85 – 2.69 (m, 4H), 1.62 – 1.49 (overlapping m, 4H), 1.51 – 1.39 (m, 2H), 1.20 (dt, *J* = 19.0, 7.3 Hz, 3H), 1.11 (td, *J* = 7.3, 1.5 Hz, 3H), 0.91 (t, *J* = 7.0 Hz, 3H) ppm; ^{13}C NMR (101 MHz, $CDCl_3$) δ 134.3 (d, *J* = 3.0 Hz), 132.1 (d, *J* = 8.5 Hz), 130.3 (d, *J* = 11.8 Hz), 117.6 (d, J = 79.0 Hz), 23.8 (d, *J* = 15.6 Hz) 23.8 (d, *J* = 4.3 Hz), 21.5 (d, *J* = 48.1 Hz), 19.3 (d, *J* = 48.4 Hz), 15.7 (d, *J* = 4.3 Hz), 15.4 (d, *J* = 16.3 Hz), 14.0 (d, *J* = 49.6 Hz), 13.5, 6.1 (d, *J* = 5.3 Hz) ppm.

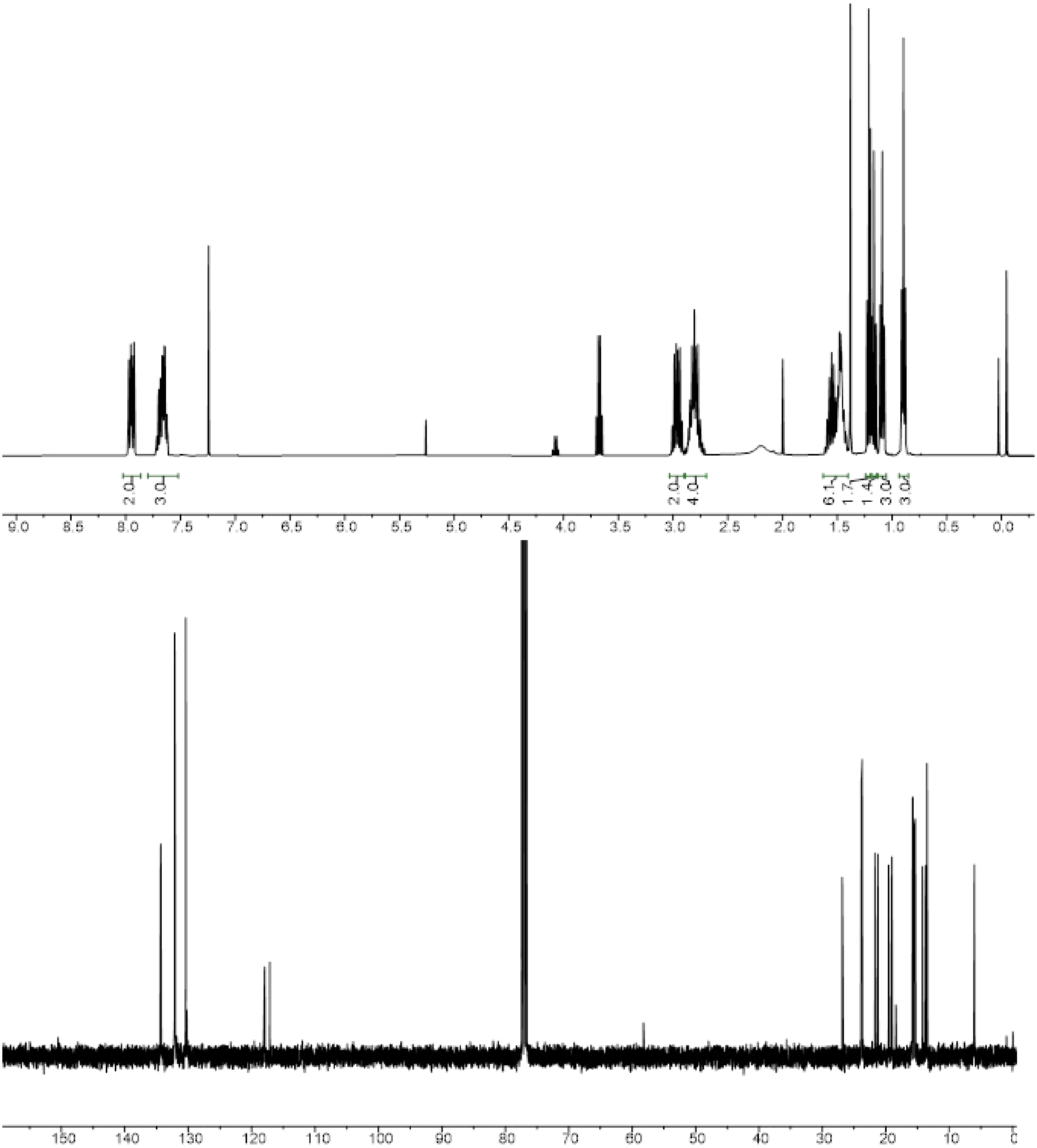

Figure S8. Top: ^{1}H NMR spectrum of **2.3-d**; bottom: ^{13}C NMR spectrum of **2.3-d.**

4.18. *n*-Butyl(ethyl)(methyl)(*n*-propyl)phosphonium chloride **2.4-a** from **4.3-b** (reaction **U4**)

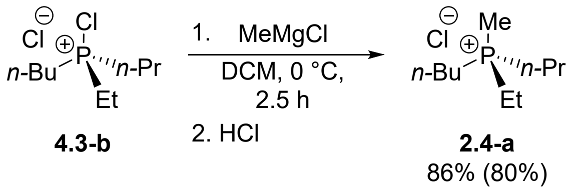

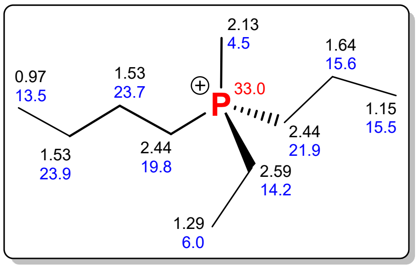

Prepared in 87% yield following general procedure B over 2.5 h at 0 °C. After column chromatography (95:5 MeCN/2-propanol to 50:50 MeCN/2-propanol) **2.4-a** was obtained as yellow oil (57 mg, 80%): HRMS (ES$^+$) m/z: calculated for $C_{10}H_{24}P^+$ 175.1616, found 175.1608. ^{31}P NMR (121 MHz, $CDCl_3$) δ 33.0 ppm; ^{1}H NMR (500 MHz, $CDCl_3$) δ 2.59 (apparent dq, *J* = 13.3, 7.7 Hz, 2H), 2.52 – 2.36 (m, 4H), 2.13 (d, *J* = 13.6 Hz, 3H), 1.64 (apparent td, *J* = 16.0, 7.3 Hz, 1H), 1.58 – 1.46 (m, 4H), 1.29 (dt, *J* = 18.9, 7.7 Hz, 3H), 1.15 (td, *J* = 7.3, 1.5 Hz, 3H), 0.97 (t, *J* = 7.0 Hz, 3H) ppm; ^{13}C NMR (126 MHz, $CDCl_3$) δ 23.9 (d, *J* = 15.3 Hz), 23.7 (d, *J* = 4.7 Hz), 21.9 (d, *J* = 48.6 Hz), 19.8 (d, *J* = 48.9 Hz), 15.6 (d, *J* = 4.8 Hz), 15.5 (d, *J* = 16.6 Hz), 14.2 (d, *J* = 50.1 Hz), 13.5, 6.0 (d, *J* = 5.4 Hz), 4.5 (d, *J* = 52.4 Hz) ppm.

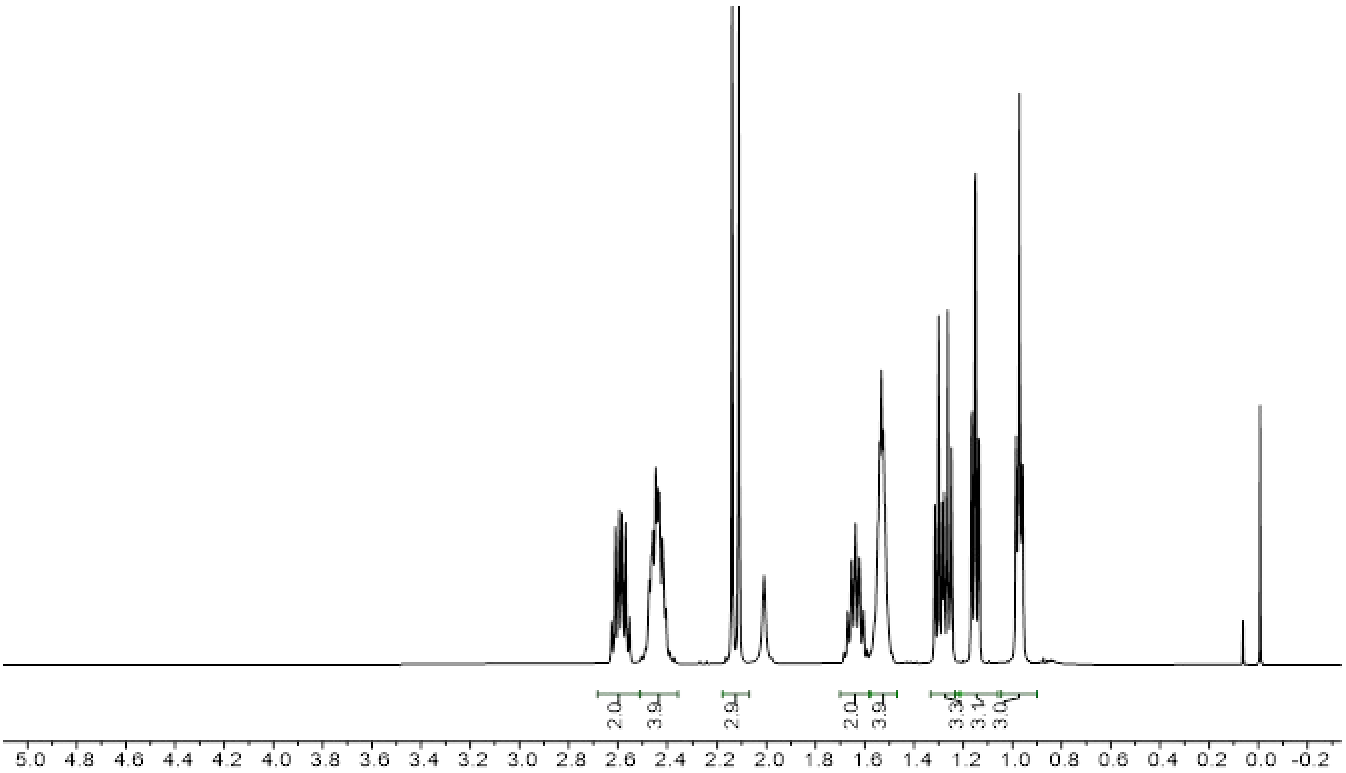

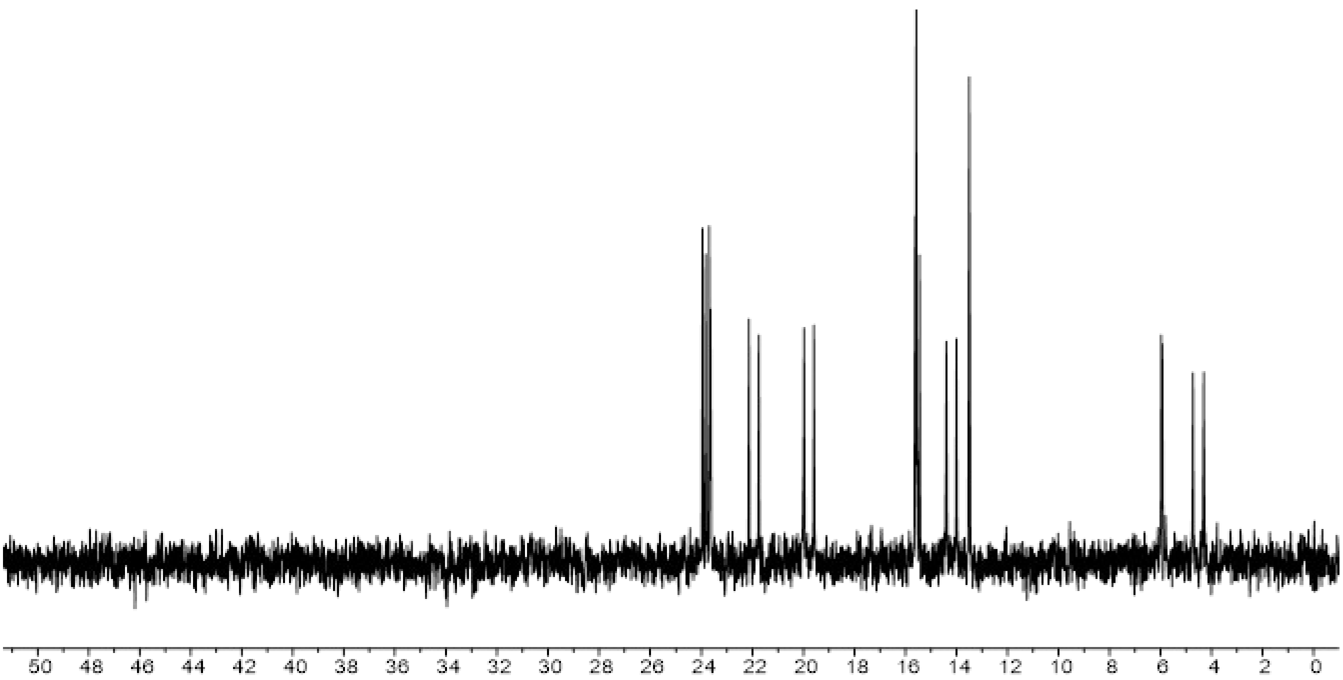

Figure S9. Top: ^{1}H NMR spectrum of **2.4-a;** bottom**:** [13]**C** NMR spectrum of **2.4-a**.

5. Phosphine Oxides: Synthesis and Characterization Data

5.1. *n*-Butyl(diphenyl)phosphine oxide **3.1-a**

Umpolung Walk**: 3.1-a** from **2.1-a** (reaction **H1**)

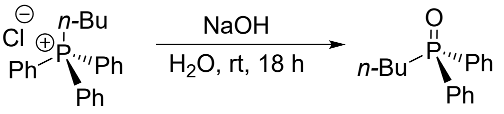

2.1-a **3.1-a** 98%

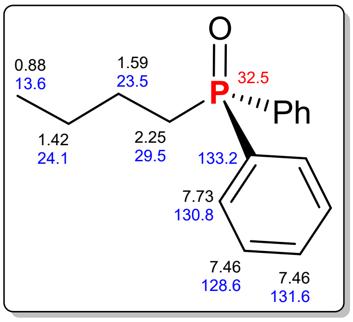

Prepared quantitatively following general procedure **C** over 18 h at room temperature and was characterized without further purification. Isolated as a white solid (3.164 g, 98%): HRMS (ES$^+$) m/z: calculated for $C_{16}H_{20}OP$ = 259.1252, found 259.1256. ^{31}P NMR (162 MHz, $CDCl_3$) δ 32.5 ppm; ^{1}H NMR (400 MHz, $CDCl_3$) δ 7.78 – 7.68 (m, 4H), 7.53 – 7.39 (m, 6H), 2.30 – 2.18 (m, 2H), 1.67 – 1.51 (m, 2H), 1.41 (apparent dq, J = 14.4, 7.3 Hz, 2H), 0.88 (t, J = 7.3 Hz, 3H) ppm; ^{13}C NMR (101 MHz, $CDCl_3$) δ 133.2 (d, J = 98.0 Hz), 131.6 (d, J = 2.7 Hz), 130.8 (d, J = 9.1 Hz), 128.6 (d, J = 11.6 Hz), 29.5 (d, J = 72.1 Hz), 24.1 (d, J = 15.1 Hz), 23.5 (d, J = 4.1 Hz), 13.6 ppm. This was previously described elsewhere.[6]

Umpolung Walk**: 3.1-a** from **2.2-g** (reaction **W3**)

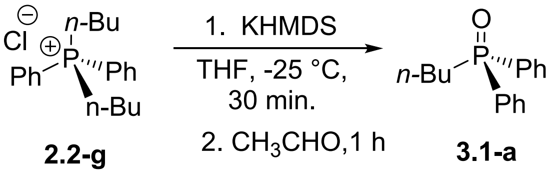

2.2-g **3.1-a**

Prepared following general procedure **D** and purified by chromatography. Isolated as a white solid (0.202 g, 88%).

5.2. Methyldiphenylphosphine oxide[7] **3.1-b** from **2.1-b** (reaction H1)

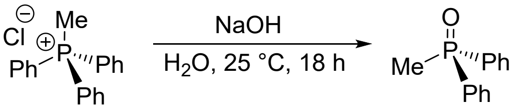

2.1-b **3.1-b** 99%

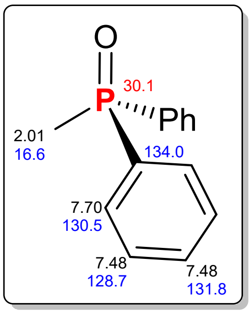

Prepared quantitatively following general procedure **C** over 18 h at room temperature and was characterized without further purification. Isolated as a white solid (3.508 g, 99%). ^{31}P NMR (162 MHz, $CDCl_3$) δ 30.1 ppm; ^{1}H NMR (400 MHz, $CDCl_3$) δ 7.78 – 7.65 (m, 4H), 7.54 – 7.38 (m, 6H), 2.01 (d, J = 13.1 Hz, 3H) ppm; ^{13}C NMR (101 MHz, $CDCl_3$) δ 134.0 (d, J = 103.7 Hz), 131.8 (d, J = 2.7 Hz), 130.5 (d, J = 9.8 Hz), 128.7 (d, J = 12.1 Hz), 16.6 (d, J = 73.6 Hz) ppm.

5.3. Ethyldiphenylphosphine oxide[8] **3.1-c** from **2.1-c** (reaction **H1**)

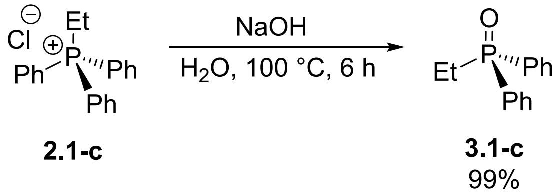

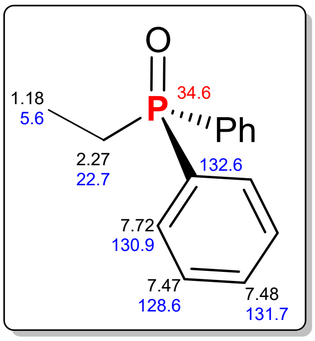

Prepared quantitatively following general procedure **C** using 15-fold excess of NaOH (5.4 M solution) over 6 h at reflux and was characterized without further purification. Isolated as white solid (487 mg, 99%). ^{31}P NMR (162 MHz, $CDCl_3$) δ 34.6 ppm; ^{1}H NMR (400 MHz, $CDCl_3$) δ 7.75-7.70 (m, 4H), 7.50-7.45 (m, 6H), 2.30-2.20 (m, 2H), 1.18 (dt, J = 17.3, 7.5 Hz, 3H) ppm; ^{13}C NMR (101 MHz, $CDCl_3$) δ 132.6 (d, J = 101.3 Hz), 131.7 (d, J = 2.5 Hz), 130.9 (d, J = 9.1 Hz), 128.6 (d, J = 11.5 Hz), 22.7 (d, J = 71.2 Hz), 5.6 (d, J = 5.0 Hz) ppm.

5.4. Di-*n*-butylphenylphosphine oxide[9] **3.2-a**

Umpolung Walk: **3.2-a** from **2.2-g** (reaction **H2**)

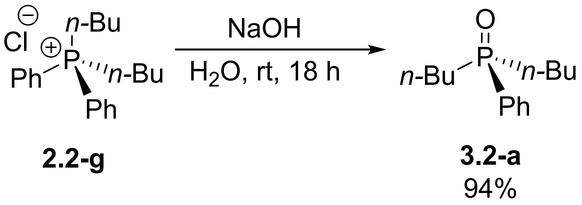

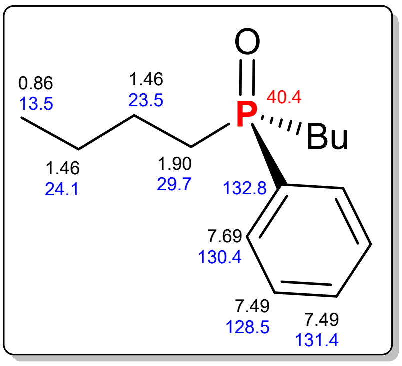

Prepared following general procedure **C** over 18 h and characterized without further purification. Isolated as a white solid (1.191 g, 94%): HRMS (ES$^+$) calculated for $C_{14}H_{24}OP$ = 239.1565, found 239.1571. ^{31}P NMR (162 MHz, $CDCl_3$) δ 40.4 ppm; ^{1}H NMR (400 MHz, $CDCl_3$) δ 7.72 – 7.65 (m, 2H), 7.53 – 7.44 (m, 3H), 2.02 – 1.78 (m, 4H), 1.68 – 1.50 (m, 8H), 0.86 (t, J = 7.2 Hz, 6H) ppm; ^{13}C NMR

(101 MHz, $CDCl_3$) δ 132.8 (d, *J* = 91.7 Hz), 131.4 (d, *J* = 2.6 Hz), 130.4 (d, *J* = 8.9 Hz), 128.5 (d, *J* = 11.0 Hz), 29.7 (d, *J* = 68.6 Hz), 24.1 (d, *J* = 14.5 Hz), 23.5 (d, *J* = 4.2 Hz), 13.5 ppm.

Umpolung Walk: **3.2-a** from **2.3-a** (reaction **W2**)

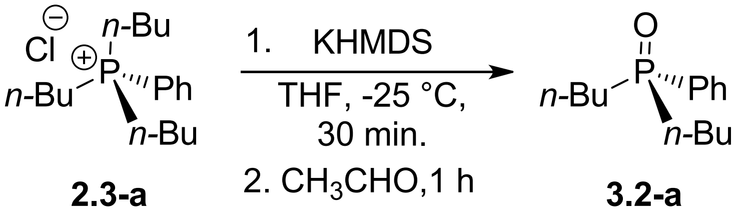

2.3-a **3.2-a**

Prepared following general procedure **D** over 1 h and purified by chromatography (233 mg, 95%).

5.5. *n*-Butyl(phenyl)(*n*-propyl)phosphine oxide **3.2-b** from **2.2-i** (reaction **H2**)

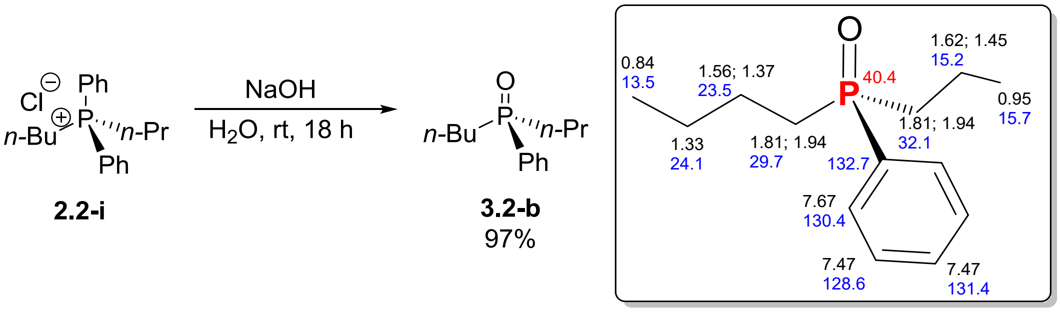

Prepared following general procedure **C** over 18 h and characterized without further purification. Isolated as white solid (2.229 g, 97%): HRMS (ES+) calculated for $C_{13}H_{22}OP$ = 225.1408, found 225.1405. ^{31}P NMR (162 MHz, $CDCl_3$) δ 40.4 ppm; ^{1}H NMR (400 MHz, $CDCl_3$) δ 7.70 – 7.62 (m, 2H), 7.50 – 7.42 (m, 3H), 2.01 – 1.87 (m, 2H), 1.87 – 1.74 (m, 2H), 1.71 – 1.52 (m, 2H), 1.51 – 1.32 (m, 4H), 0.95 (td, *J* = 7.3 Hz, *J* = 1.1 Hz, 3H), 0.84 (t, *J* = 7.2 Hz, 3H) ppm; ^{13}C NMR (101 MHz, $CDCl_3$) δ 132.7 (d, *J* = 91.5 Hz), 131.4 (d, *J* = 2.7 Hz), 130.4 (d, *J* = 8.7 Hz), 128.6 (d, *J* = 11.0 Hz), 32.1 (d, *J* = 68.3 Hz), 29.7 (d, *J* = 68.3 Hz), 24.1 (d, *J* = 14.5 Hz), 23.5 (d, *J* = 4.2 Hz), 15.7 (d, *J* = 15.1 Hz), 15.2 (d, *J* = 4.1 Hz), 13.5 ppm.

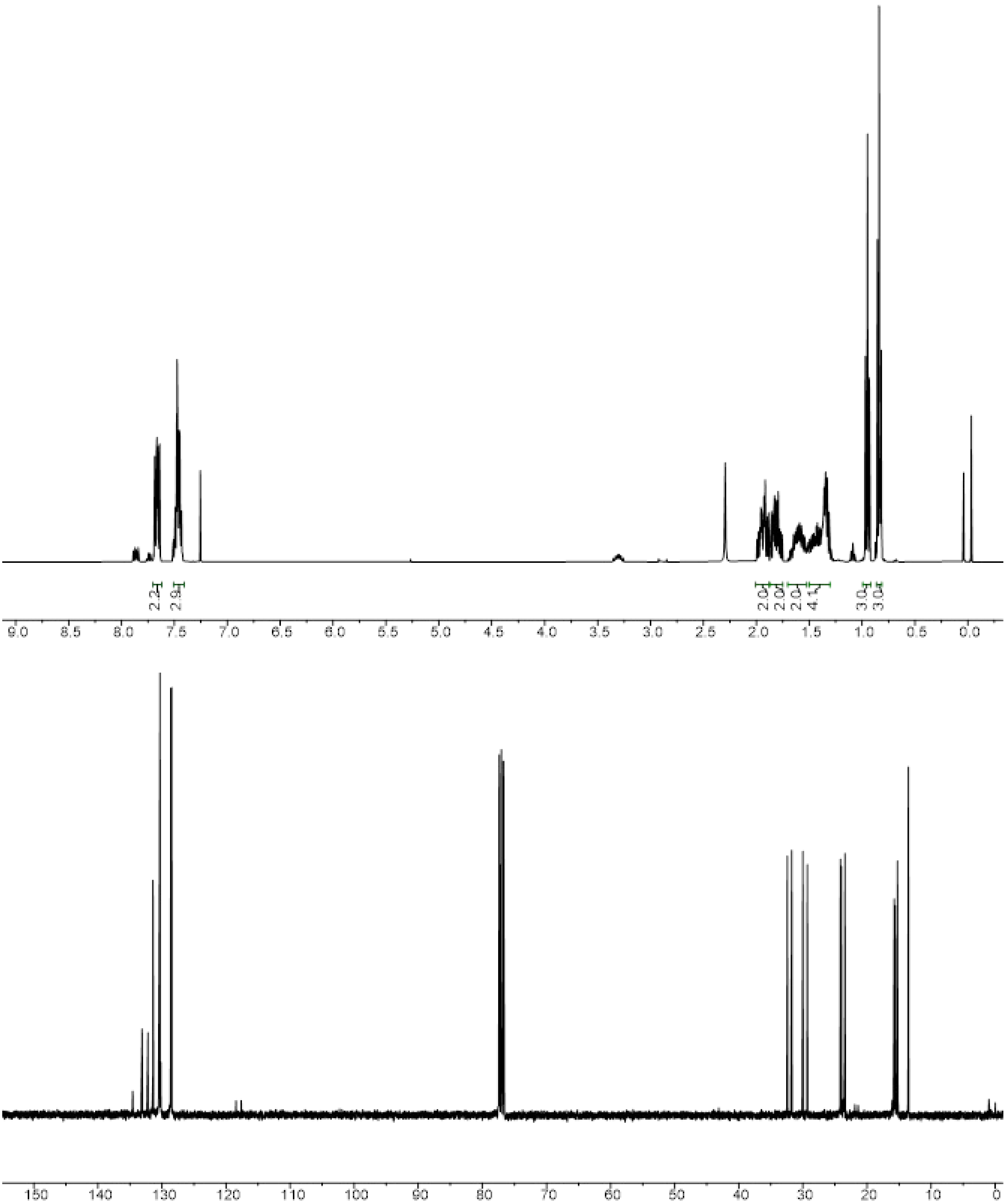

Figure S10. Top: ^{1}H NMR spectrum of **3.2-b;** bottom**:** [13]**C** NMR spectrum of **3.2-b**.

5.6. *n*-Butyl(ethyl)(*n*-propyl)phosphine oxide **3.3-a** from **2.3-d** (reaction **H3**)

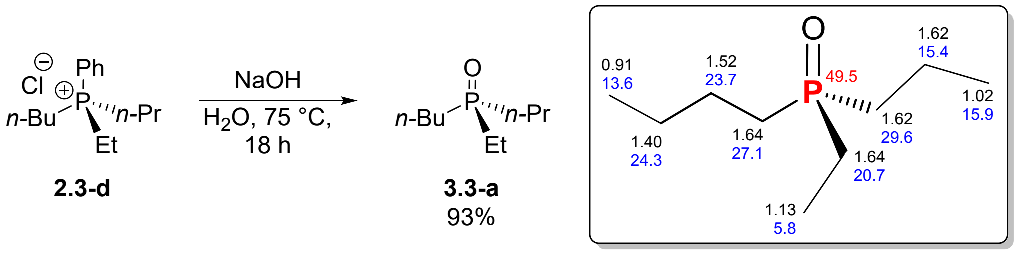

Prepared following general procedure **C** using 7.6-fold excess of NaOH (12.6 M solution) over 18 h at 75 °C and characterized without further purification.ii Isolated as clear oil (480 mg, 93%): HRMS (ES$^+$) calculated for $C_9H_{22}OP$ = 177.1408, found 177.1414. ^{31}P NMR (121 MHz, $CDCl_3$) δ 49.5 ppm; ^{1}H NMR (500 MHz, $CDCl_3$) δ 1.72 – 1.55 (m, 8H), 1.56 – 1.47 (m, 2H), 1.40 (h, *J* = 8.0 Hz, 2H), 1.13 (dt, *J* = 16.3 Hz, *J* = 7.7 Hz, 3H), 1.02 (td, *J* = 7.0 Hz, *J* = 1.2 Hz, 3H), 0.91 (t, *J* = 7.3 Hz, 3H) ppm; ^{13}C NMR (126 MHz, $CDCl_3$) δ 29.6 (d, *J* = 64.9 Hz), 27.1 (d, *J* = 64.9 Hz), 24.3 (d, *J* = 13.9 Hz), 23.7 (d, *J* = 4.1 Hz), 20.7 (d, *J* = 65.9 Hz), 15.9 (d, *J* = 14.7 Hz), 15.4 (d, *J* = 3.8 Hz), 13.6, 5.8 (d, *J* = 4.8 Hz) ppm.

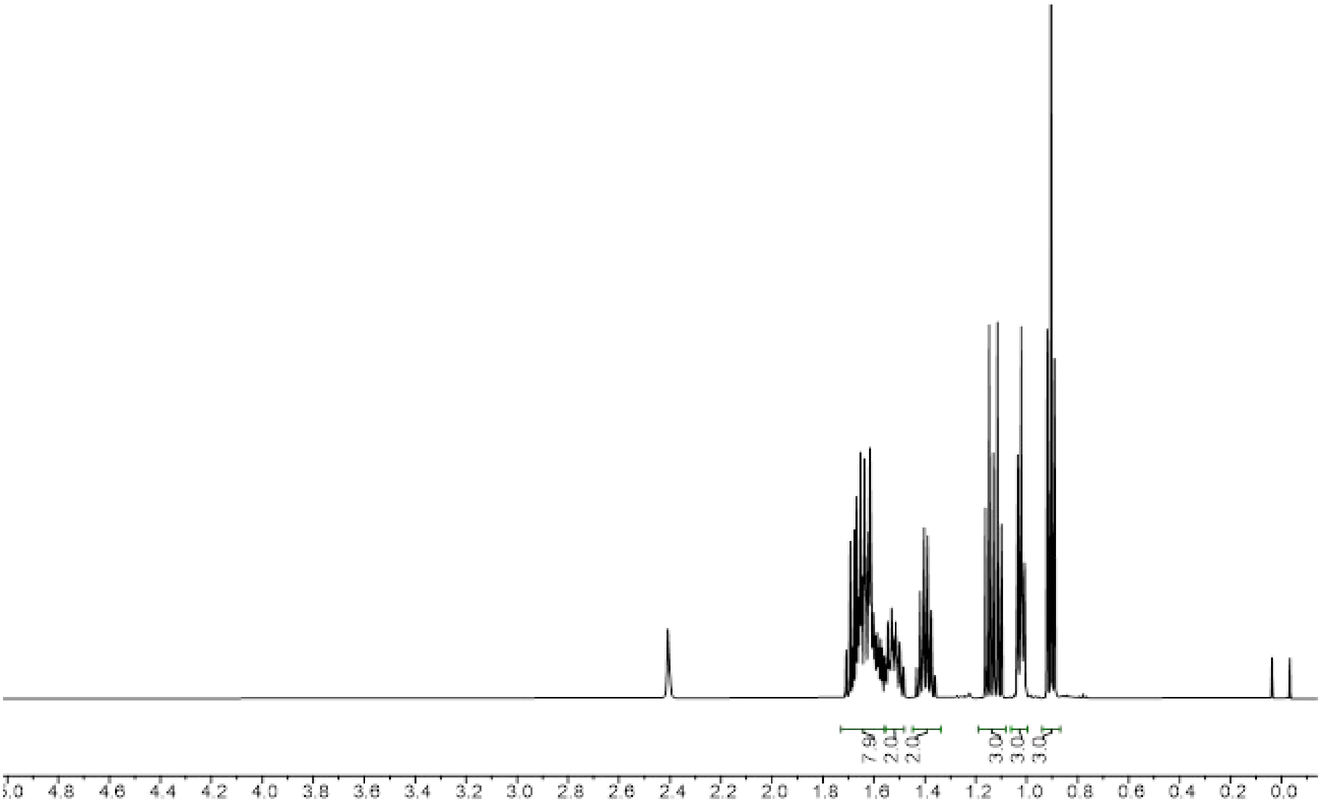

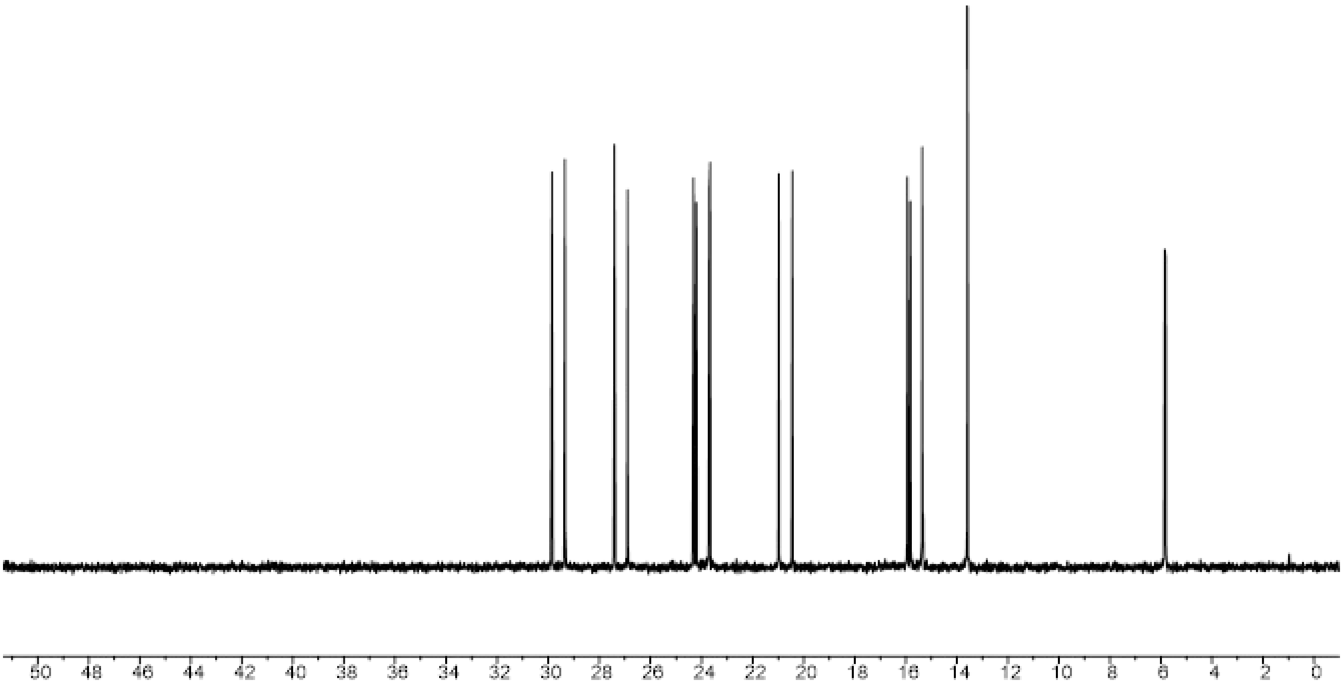

Figure S11. Top: ^{1}H NMR spectrum of **3.3-a**; bottom: ^{13}C NMR spectrum of **3.3-a**.

5.7. Ethyldiphenylphosphine oxide **3.1-c** from **5.1-b** (Main text, Scheme 10)

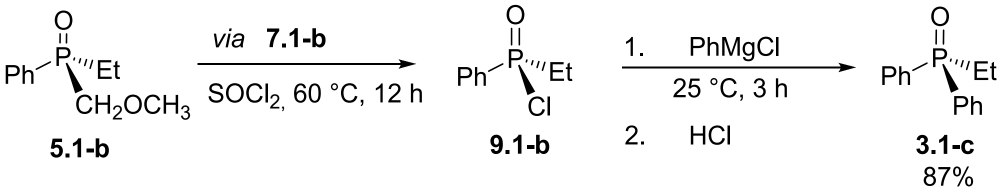

The chloride **9.1-b** was prepared in 92% yield (Section 8.10 below). The solvent was removed under reduced pressure and the residue (26 mg, 0.14 mmol, 1.00 equivalent) was redissolved in DCM (0.70 ml) to give a 0.2 M solution. Under agitation, phenylmagnesium chloride (2.0 M in THF, 0.08 mL, 0.16 mmol, 1.14 equivalents) was added. The resultant reaction mixture was stirred at 25 °C for three hours. After this time, the reaction mixture was quenched by 2.0 M HCl in diethyl ether (0.08 mL, 1.14 equivalents) and formation of a white precipitate was observed. This was diluted with DCM (10 mL), then washed with water (2 x 1.50 mL) and brine (1 x 3.00 mL). The DCM phase was dried over magnesium sulphate and concentrated *in vacuo* to yield an oily residue which crystallized on standing. ^{31}P NMR (121 MHz, $CDCl_3$) spectroscopic analysis of the crude product indicated the presence of **3.1-c** and two unknown species in a 97:1:2 ratio. Please refer to Section 5.3 for characterization of **3.1c**.

5.8. Methyldiphenylphosphine oxide **3.1-b** from **5.1-a** (Main text Scheme 10)

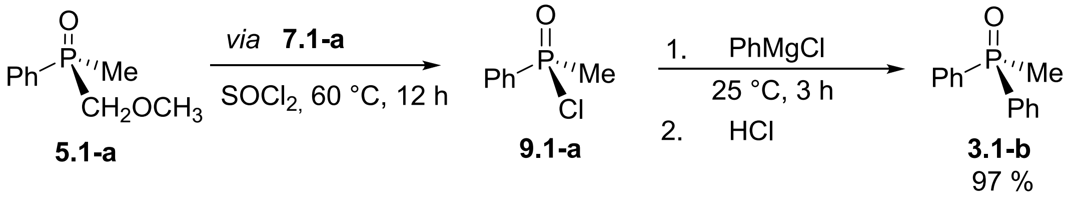

The chloride **9.1-a** was prepared in 92% yield (Section 8.6 below). The solvent was removed under reduced pressure and the residue (42 mg, 0.24 mmol, 1.00 equivalent) was redissoved in DCM (1.20 ml) to give a 0.2 M solution. The solution was treated with phenylmagnesium chloride (2.0 M in THF, 0.15 mL, 0.30 mmol, 1.25 equivalents) and the resultant reaction mixture was stirred at 25 °C for three hours. After this time, the reaction mixture was quenched by the addition of 2.0 M HCl in diethyl ether (0.15 mL, 1.25 equivalents. Then, DCM (15 mL) was added and the organic phase was washed with water (2 x 1.50 mL) and brine (1 x 3.00 mL). The DCM phase was dried over magnesium sulphate and concentrated *in vacuo* to yield an oily residue which crystallized on standing (45 mg, 82%). For characterization of **3.1-b**, please refer to Section 5.2.

6. MOM-derived Quaternary Phosphonium Salts 6

6.1. (Methoxymethyl)triphenylphosphonium chloride[10] **6.0-a** from **1.0-a**

This material is available commercially.

Alternatively, it can be prepared following a published procedure and isolated as a white solid (in 96% yield): ^{31}P NMR (121 MHz, $CDCl_3$) δ 17.6 ppm; ^{1}H NMR (400 MHz, $CDCl_3$) δ 7.80-7.75 (m, 9H), 7.69-7.60 (m, 6H), 5.81 (d, J = 4.0 Hz, 2H), 3.66 (d, J = 0.8 Hz, 3H); ^{13}C NMR (101 MHz, $CDCl_3$) δ 135.3 (d, J = 3.1 Hz), 134.1 (d, J = 10.0 Hz), 130.4 (d, J = 12.7 Hz), 116.6 (d, J = 85.9 Hz), 65.9 (d, J = 68.6 Hz), 62.7 (d, J = 13.4 Hz).

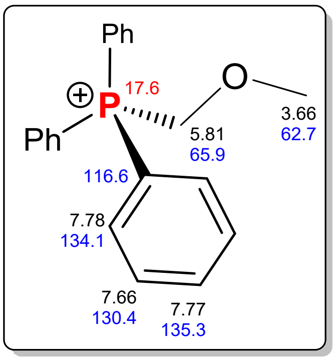

6.2. (Methoxymethyl)(methyl)diphenylphosphonium chloride **6.1-a** from **7.0-a**

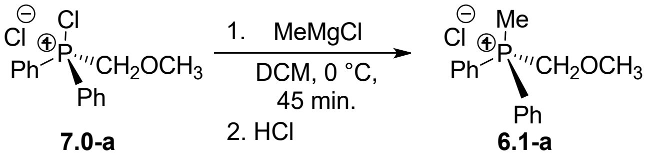

7.0-a

6.1-a
89% (85%)

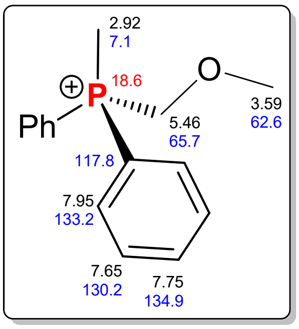

Prepared in 89% yield following general procedure **B** at 0 °C. Recrystallization from chloroform/ethyl acetate gave **6.1-a** (239 mg, 85%) as transparent crystalline sheets: HRMS (ES$^+$) calculated for $C_{15}H_{18}OP^+$ 245.1095, found 245.1098. ^{31}P NMR (121 MHz, $CDCl_{3zz}$) δ 18.6 ppm; ^{1}H NMR (500 MHz, $CDCl_3$) δ 7.99 – 7.91 (m, 4H), 7.78 – 7.71 (m, 2H), 7.70 – 7.62 (m, 4H), 5.46 (d, J = 4.4 Hz, 2H), 3.59 (d, J = 0.7 Hz, 3H), 2.92 (d, J = 14.2 Hz, 3H) ppm; ^{13}C NMR (126 MHz, $CDCl_3$) δ 134.9 (d, J = 3.0 Hz), 133.2 (d, J = 10.1 Hz), 130.2 (d, J = 12.8 Hz), 117.8 (d, J = 84.5 Hz), 65.7 (d, J = 67.9 Hz), 62.6, 7.1 (d, J = 53.4 Hz) ppm.

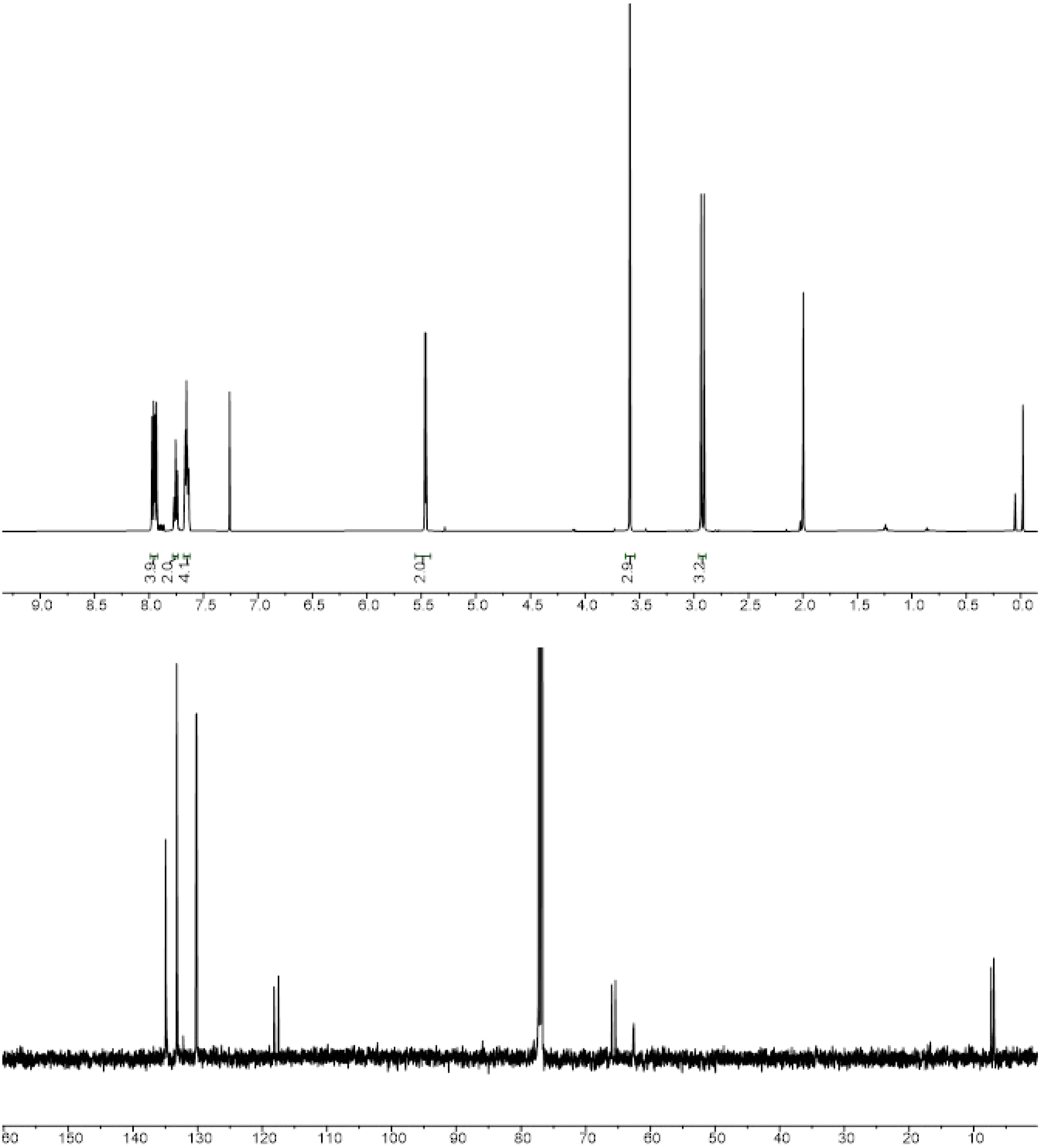

Figure S12. Top: ^{1}H NMR spectrum of **6.1-a**; ^{13}C NMR spectrum of **6.1-a.**

6.3. (Ethyl)(methoxymethyl)diphenylphosphonium chloride **6.1-b** from **7.0-a**

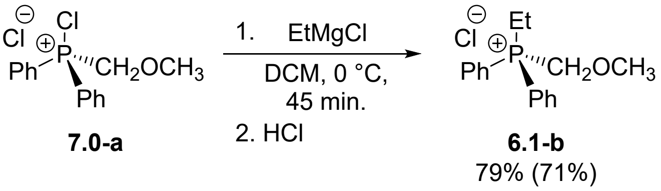

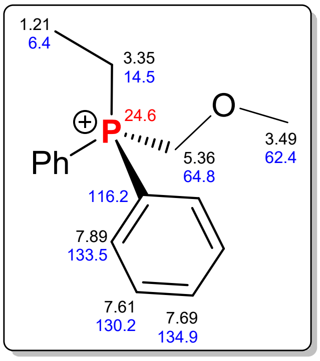

Prepared in 79% yield following general procedure **B** at 0 °C. Recrystallization from chloroform/ethyl acetate gave **6.1-b** (1.737 g, 71%) as white crystals: HRMS (ES$^+$) calculated for $C_{16}H_{20}OP^+$ 259.1252, found 259.1245. ^{31}P NMR (162 MHz, $CDCl_3$) δ 24.6 ppm; ^{1}H NMR (400 MHz, $CDCl_3$) δ 7.97 – 7.80 (m, 4H), 7.74 – 7.67 (m, 2H), 7.66 – 7.54 (m, 4H), 5.36 (d, *J* = 4.4 Hz, 2H), 3.49 (d, *J* = 0.8 Hz, 3H), 3.35 (dq, *J* = 13.2, 7.5 Hz, 2H), 1.21 (dt, *J* = 19.8, 7.6 Hz, 3H) ppm; ^{13}C NMR (101 MHz, $CDCl_3$) δ 134.9 (d, *J* = 3.1 Hz), 133.5 (d, *J* = 9.4 Hz), 130.2 (d, *J* = 12.2 Hz), 116.2 (d, *J* = 82.1 Hz), 64.8 (d, *J* = 66.4 Hz), 62.4 (d, *J* = 12.7 Hz), 14.5 (d, *J* = 48.7 Hz), 6.4 (d, *J* = 5.4 Hz) ppm.

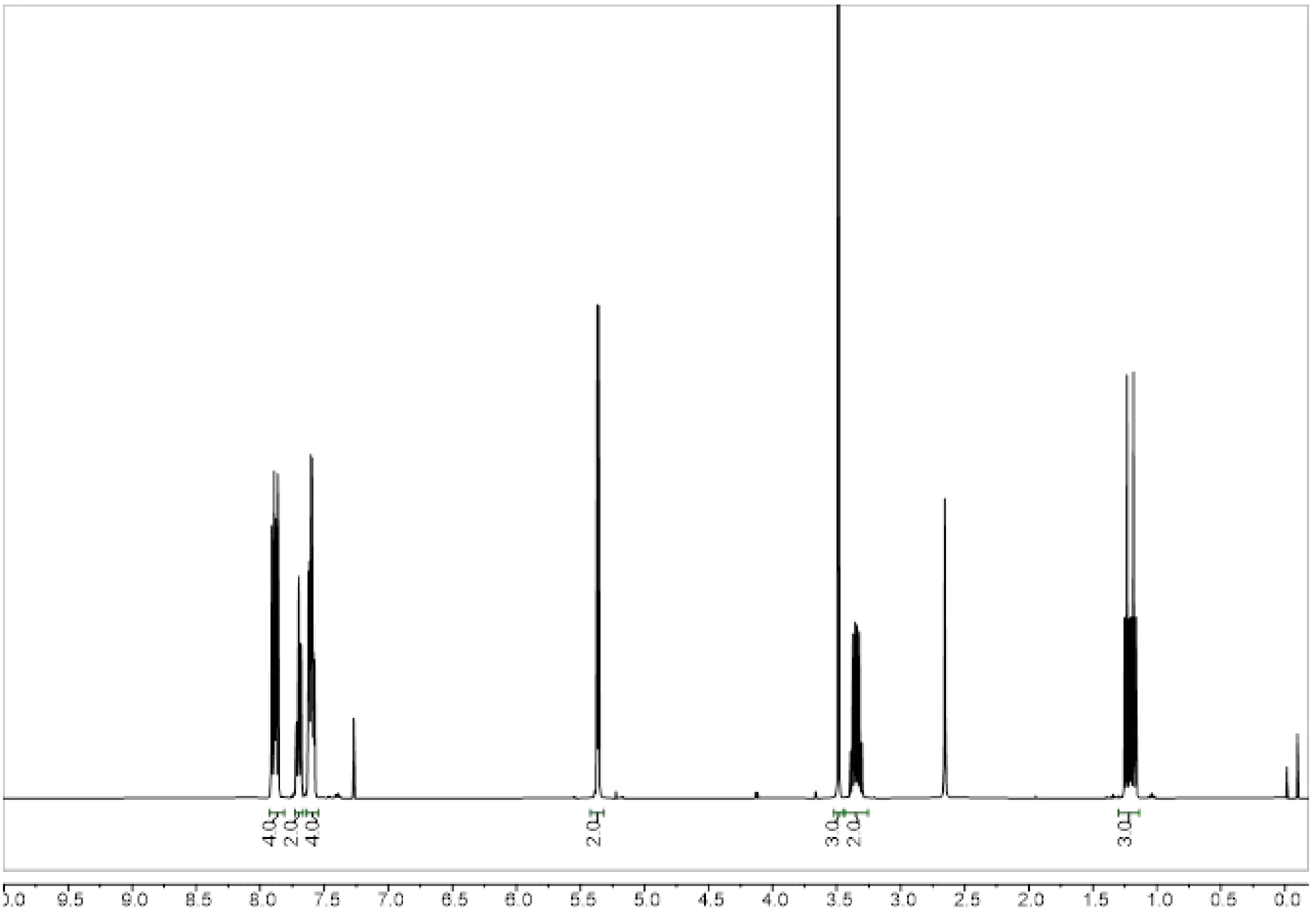

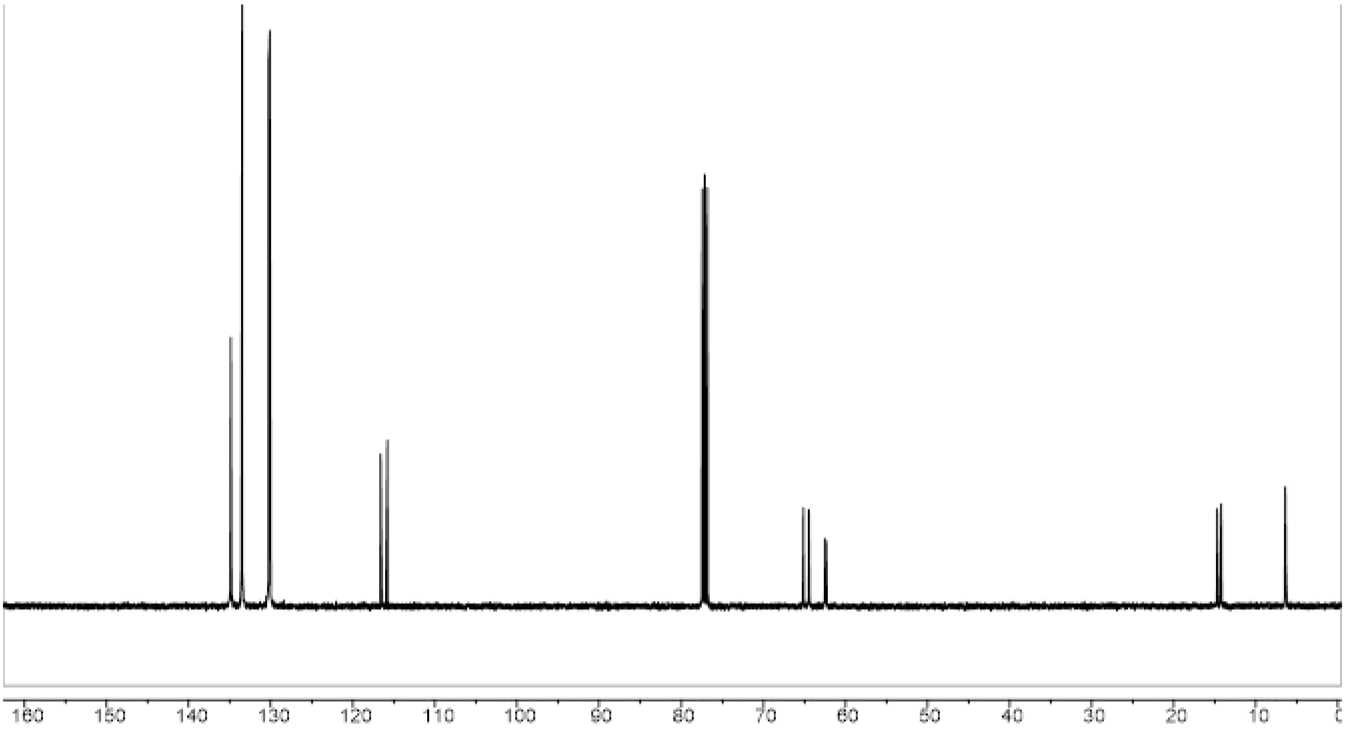

Figure S13. Top: ^{1}H NMR spectrum of **6.1-b**; ^{13}C NMR spectrum of **6.1-b.**

6.4. (*n*-Butyl)(methoxymethyl)diphenylphosphonium chloride **6.1-c** from **7.0-a**

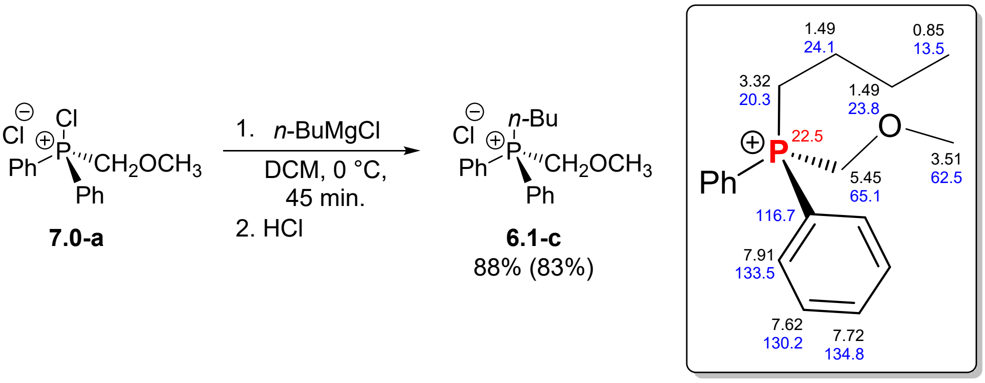

Prepared in 88% yield following general procedure **B** at 0 °C. Recrystallization from chloroform/ethyl acetate gave **6.1-c** (268 mg, 83%) as fine white crystals. ^{31}P NMR (162 MHz, $CDCl_3$) δ 22.5 ppm; ^{1}H NMR (400 MHz, $CDCl_3$) δ 7.96 – 7.85 (m, 4H), 7.76 – 7.68 (m, 2H), 7.66 – 7.58 (m, 4H), 5.45 (d, *J* = 4.2 Hz, 2H), 3.51 (d, *J* = 0.8 Hz, 3H), 3.38 – 3.25 (m, 2H), 1.58 – 1.38 (m, 4H), 0.85 (t, *J* = 7.0 Hz, 3H) ppm; ^{13}C NMR (101 MHz, $CDCl_3$) δ 134.8 (d, *J* = 3.1 Hz), 133.5 (d, *J* = 9.3 Hz), 130.2 (d, *J* = 12.2 Hz), 116.7 (d, *J* = 82.2 Hz), 65.1 (d, *J* = 66.0 Hz), 62.5 (d, *J* = 12.5 Hz), 24.1 (d, *J* = 4.8 Hz), 23.8 (d, *J* = 16.1 Hz), 20.3 (d, *J* = 47.1 Hz), 13.5 ppm.

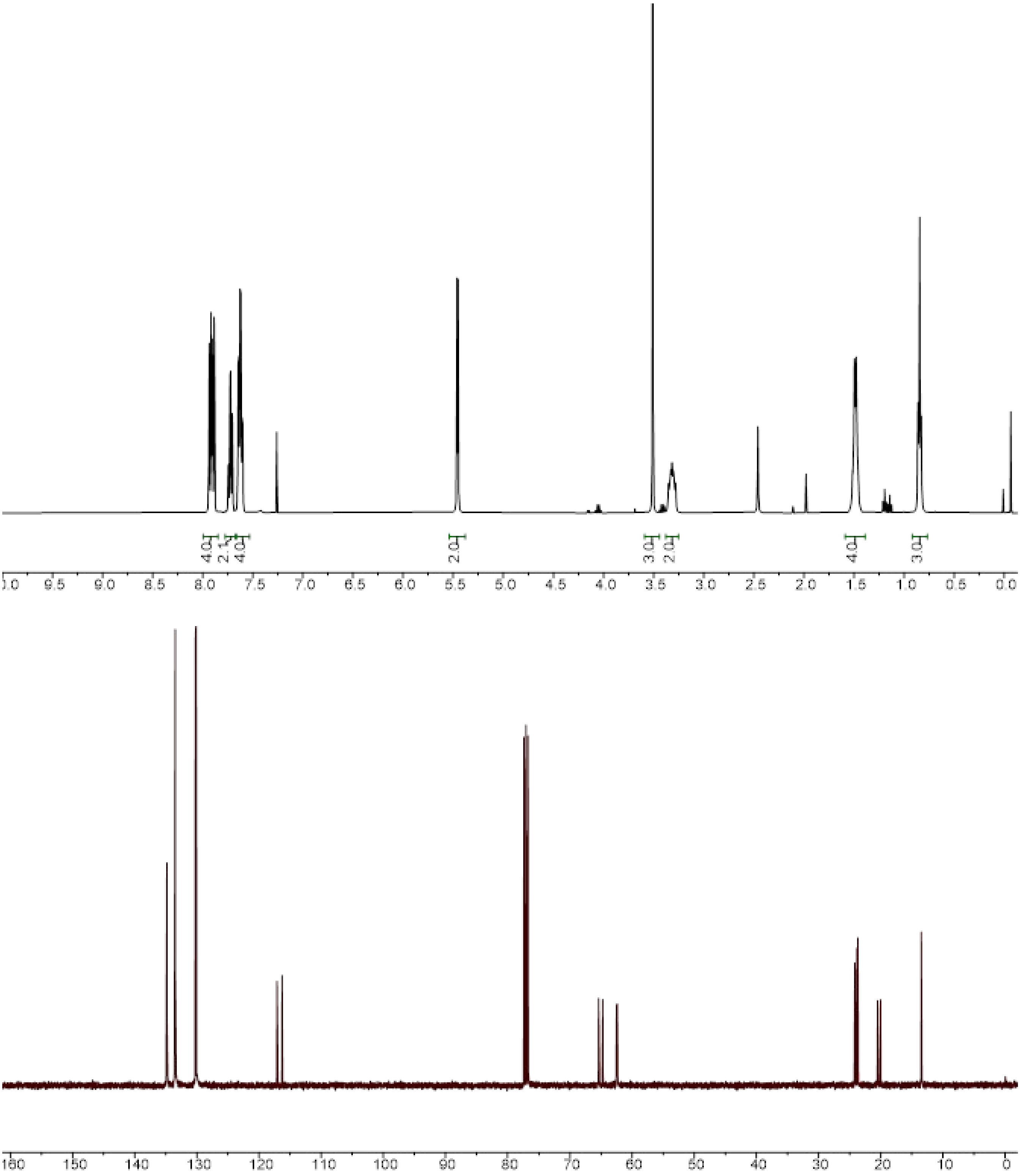

Figure S14. Top: ^{1}H NMR spectrum of **6.1-c**; ^{13}C NMR spectrum of **6.1-c.**

6.5. (*iso*-Butyl)(methoxymethyl)diphenylphosphonium chloride **6.1-d** from **7.0-a**

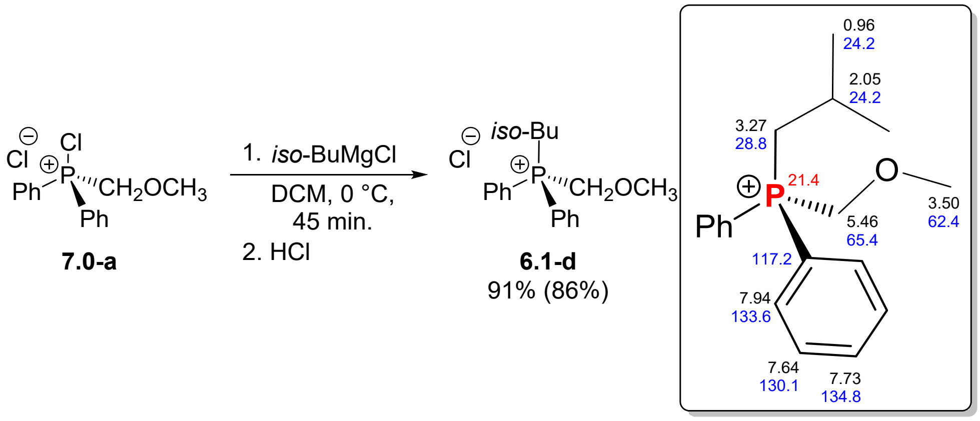

Prepared in 91% yield following general procedure **B** at 0 °C. Recrystallization from chloroform/ethyl acetate afforded **6.1-d** (278 mg, 86%) as white crystals. ^{31}P NMR (162 MHz, $CDCl_3$) δ 21.4 ppm; ^{1}H NMR (400 MHz, $CDCl_3$) δ 7.98 – 7.88 (m, 4H), 7.78 – 7.68 (m, 2H), 7.69 – 7.58 (m, 4H), 5.46 (d, J = 4.1 Hz, 2H), 3.50 (d, J = 0.8 Hz, 2H), 3.27 (dd, J = 13.5, 6.6 Hz, 2H), 2.10 – 1.97 (m, 1H), 0.96 (dd, J = 6.7, 1.0 Hz, 6H) ppm; ^{13}C NMR (101 MHz, $CDCl_3$) δ 134.8 (d, J = 3.1 Hz), 133.6 (d, J = 9.5 Hz), 130.1 (d, J = 12.2 Hz), 117.2 (d, J = 81.9 Hz), 65.4 (d, J = 65.6 Hz), 62.4 (d, J = 12.4 Hz), 28.8 (d, J = 44.6 Hz), 24.2 (d, J = 9.5 Hz), 24.2 (d, J = 5.3 Hz) ppm.

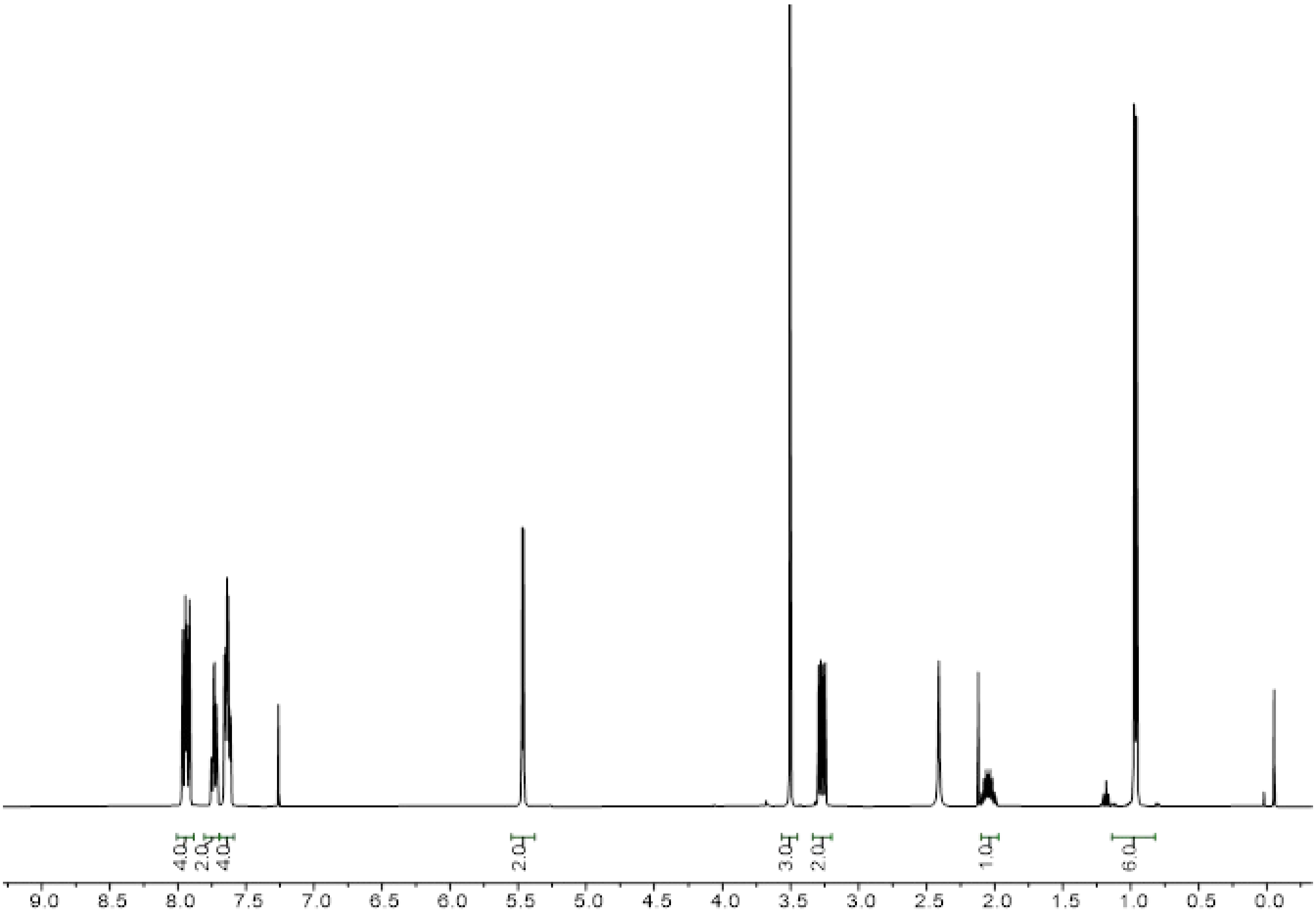

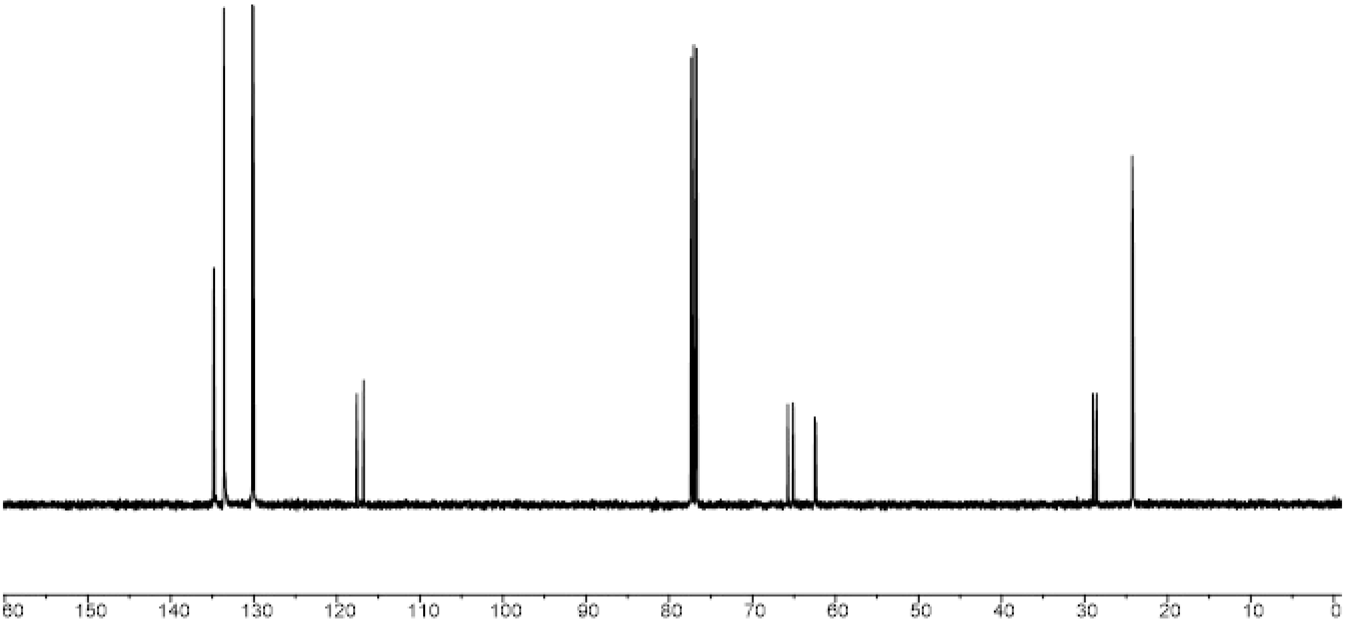

Figure S15. Top: ^{1}H NMR spectrum of **6.1-d**; ^{13}C NMR spectrum of **6.1-d.**

6.6. (Benzyl)(methoxymethyl)diphenylphosphonium chloride[11] **6.1-e** from **7.0-a**

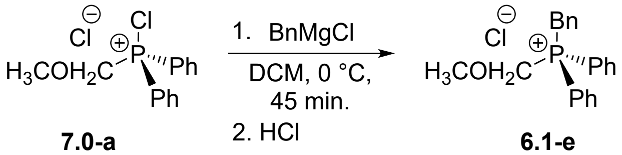

7.0-a **6.1-e** 62%

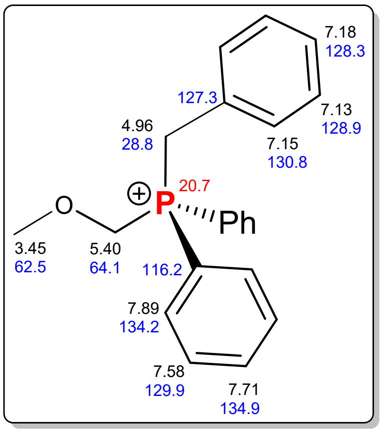

Prepared following general procedure **B** at 0 °C. Precipitation from chloroform/ethyl acetate afforded **6.1-e** (199 mg, 62%) as off-white solid: HRMS (ES$^+$) calculated for $C_{21}H_{22}OP^+$ 321.1407, found 321.1407. ^{31}P NMR (121 MHz, $CDCl_3$) δ 20.7 ppm; ^{1}H NMR (500 MHz, $CDCl_3$) δ 7.93 – 7.83 (m, 4H), 7.78 – 7.67 (m, 2H), 7.58 (td, *J* = 7.8 Hz, *J* = 3.4 Hz, 4H), 7.22 – 7.09 (m, 5H), 5.40 (d, *J* = 4.0 Hz, 2H), 4.96 (d, *J* = 15.4 Hz, 2H), 3.45 (d, *J* = 0.8 Hz, 3H) ppm; ^{13}C NMR (126 MHz, $CDCl_3$) δ 134.9 (d, *J* = 2.9 Hz), 134.2 (d, *J* = 9.2 Hz), 130.8 (d, *J* = 5.5 Hz), 129.9 (d, *J* = 12.2 Hz), 128.9 (d, *J* = 3.3 Hz), 128.3 (d, *J* = 3.8 Hz), 127.3 (d, *J* = 8.6 Hz), 116.2 (d, *J* = 82.0 Hz), 64.1 (d, *J* = 66.4 Hz), 62.5 (d, *J* = 12.1 Hz), 28.8 (d, *J* = 43.9 Hz) ppm.

7. **Preparation of MOM-derived phosphine oxides 5.0-a, 5.1-a, 5.1-b** from salts **6**

7.1. Methoxymethyldiphenylphosphine oxide[12] **5.0-a** from **6.0-a**

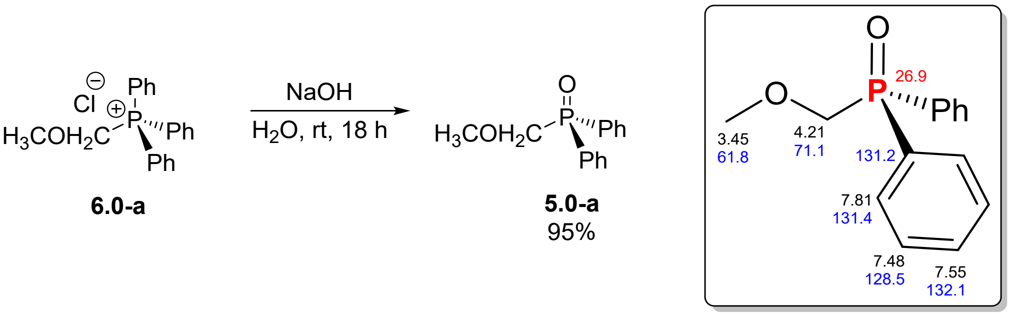

Prepared quantitatively using general procedure **C**. **5.0-a** (8.655 g, 95%) was isolated as a white solid: HRMS (ES+) m/z: calculated for $C_{14}H_{15}O_2NaP$ = 269.0707, found 269.0697. ^{31}P NMR (162 MHz, $CDCl_3$) δ 26.9 ppm; ^{1}H NMR (400 MHz, $CDCl_3$) δ 7.87 – 7.75 (m, 4H), 7.61 – 7.51 (m, 2H), 7.51 – 7.44 (m, 4H), 4.21 (d, *J* = 6.3 Hz, 2H), 3.45 (d, *J* = 0.7 Hz, 3H) ppm; ^{13}C NMR (101 MHz, $CDCl_3$) δ 132.1 (d, *J* = 2.8 Hz), 131.4 (d, *J* = 9.5 Hz), 131.2 (d, *J* = 98.7 Hz), 128.5 (d, *J* = 11.9 Hz), 71.1 (d, *J* = 87.9 Hz), 61.8 (d, *J* = 11.7 Hz) ppm

7.2. Methyl(methoxymethyl)phenylphosphine oxide **5.1-a** from **6.1-a**

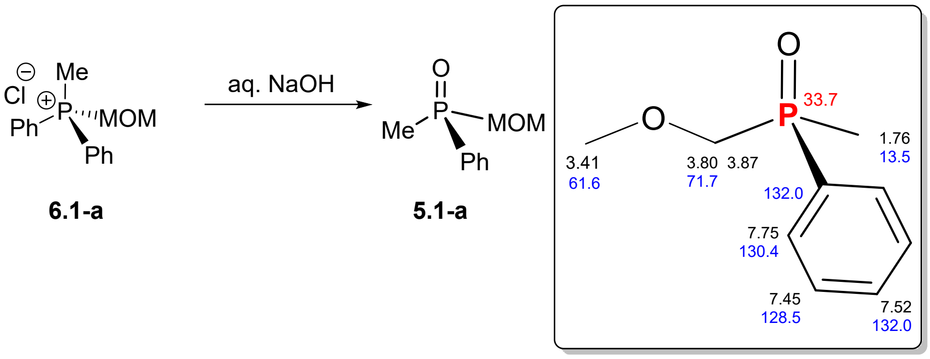

Prepared using 5 equivalents NaOH at room temperature according to general procedure **C** (350 mg, 94%). HRMS (ES^{+}) m/z: calculated for $C_{10}H_{16}O_2P$ = 185.0731 found 185.0733. ^{31}P NMR (121 MHz, $CDCl_3$) δ 33.7 ppm; ^{1}H NMR (400 MHz, $CDCl_3$) δ 7.75 (dd, *J* = 12 Hz, 8.0 Hz, 2H), 7.52 (t, *J* = 8.0 Hz, 1H), 7.45 (t, *J* = 8.0 Hz, 2H), 3.87 (dd, *J* = 12.7, 7.7 Hz, 1H), 3.80 (dd, *J* = 12.7, 6.0 Hz, 1H), 3.41 (s, 3H), 1.76 (d, *J* = 13.4 Hz, 3H) ppm; ^{13}C NMR (101 MHz, $CDCl_3$) δ 132.0 (d, *J* = 96 Hz), 132.0 (d, *J* = 3 Hz), 130.4 (d, *J* = 9.5 Hz), 128.5 (d, *J* = 11.6 Hz), 71.7 (d, *J* = 87 Hz), 61.6 (d, *J* = 12.6 Hz), 13.5 (d, *J* = 70 Hz) ppm.

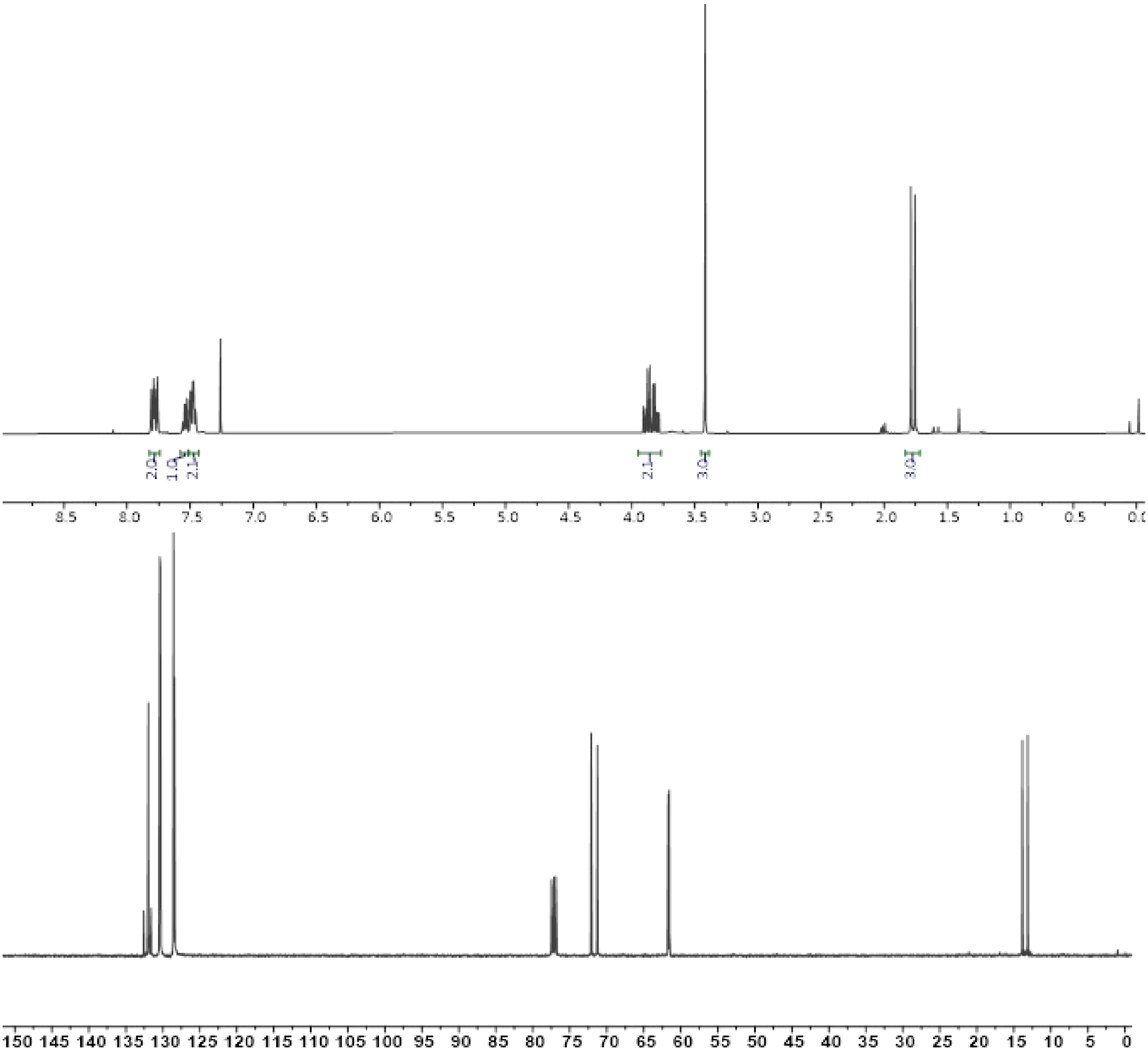

Figure S16. Top: ^{1}H NMR spectrum of **5.1-a**; bottom: ^{13}C NMR spectrum of **5.1-a.**

7.3. Ethyl(methoxymethyl)phenylhosphine oxide **5.1-b** from **6.1-b**

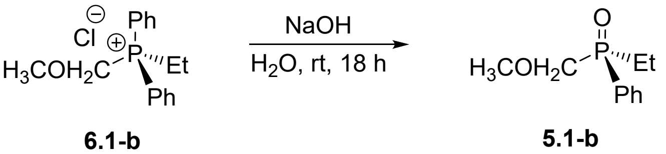

6.1-b **5.1-b** 97%

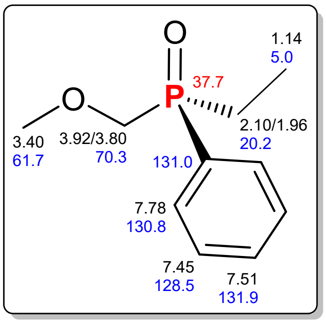

Prepared quantitatively using ca. 10 equivalents NaOH at room temperature according to general procedure **C** (601 mg, 96%). HRMS (ES^{+}) m/z: calculated for $C_{10}H_{16}O_2P$ = 199.0888, found 199.0888. ^{31}P NMR (121 MHz, $CDCl_3$) δ 37.7 ppm; ^{1}H NMR (400 MHz, $CDCl_3$) δ 7.82 – 7.74 (m, 2H), 7.57 – 7.46

(overlapping m, 1H), 7.48 – 7.42 (overlapping m, 2H), 3.92 (dd, *J* = 12.8, 7.0 Hz, 1H), 3.80 (dd, *J* = 12.8, 7.0 Hz, 1H), 3.40 (d, *J* = 0.7 Hz, 3H), 2.10 (apparent dtd, *J* = 15.2, 7.6 Hz, 3.2 Hz, 1H), 1.96 (apparent dtd, *J* = 15.2, 7.6, 3.2 Hz, 1H), 1.14 (dt, *J* = 17.2, 7.7 Hz, 3H) ppm; ^{13}C NMR (101 MHz, $CDCl_3$) δ 131.9 (d, *J* = 2.8 Hz), 131.0 (d, *J* = 93.0 Hz), 130.8 (d, *J* = 8.9 Hz), 128.5 (d, *J* = 11.4 Hz), 70.3 (d, *J* = 84.4 Hz), 61.7 (d, *J* = 12.6 Hz), 20.2 (d, *J* = 69.4 Hz), 5.0 (d, *J* = 5.3 Hz) ppm.

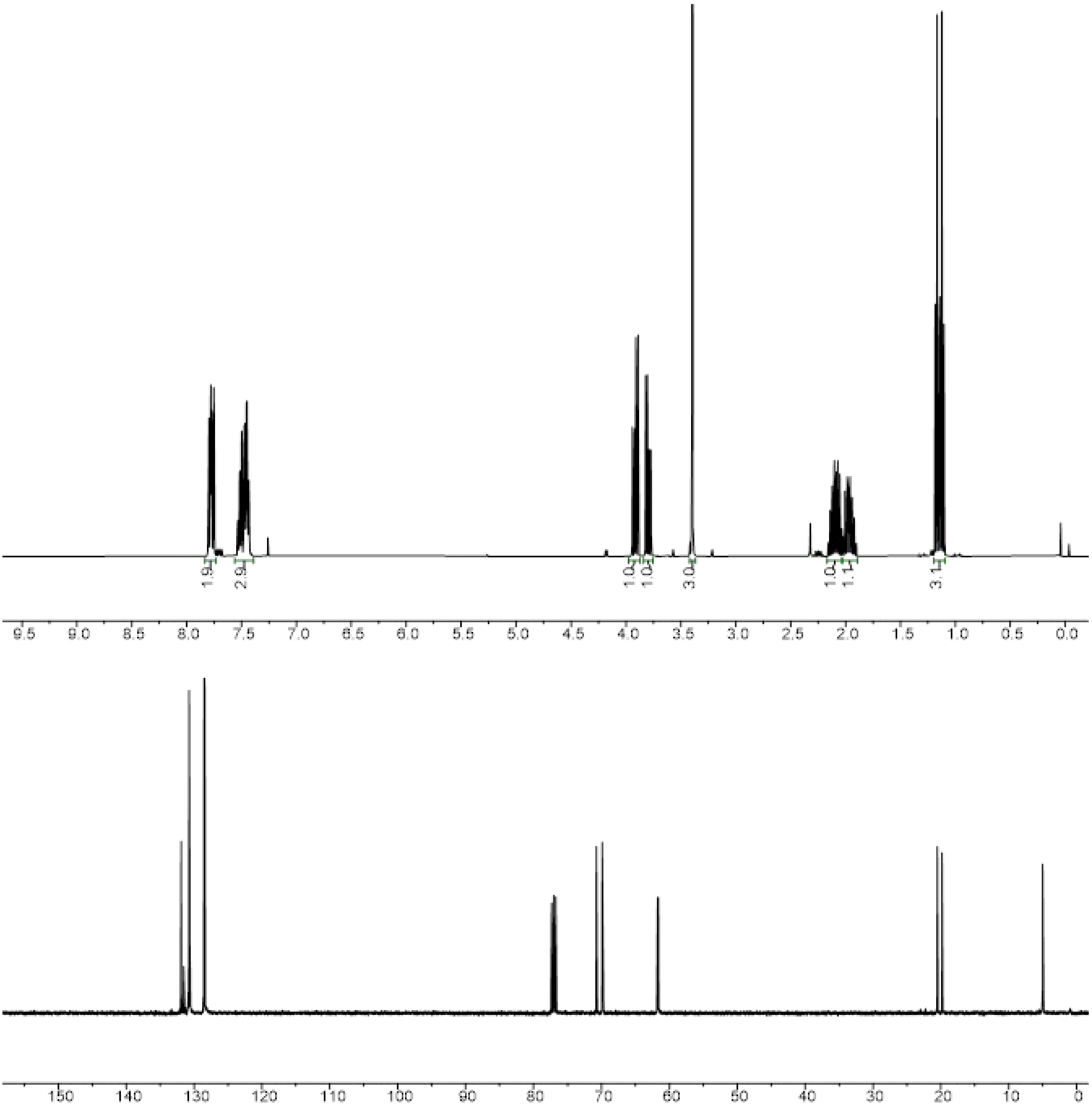

Figure S17. Top: ^{1}H NMR spectrum of **5.1-b**; bottom: ^{13}C NMR spectrum of **5.1-b.**

8. MOM-derived P-chlorophosphonium salts 7

8.1. Chloro(methoxymethyl)diphenylphosphonium chloride **7.0-a** from phosphine oxide **5.0-a**

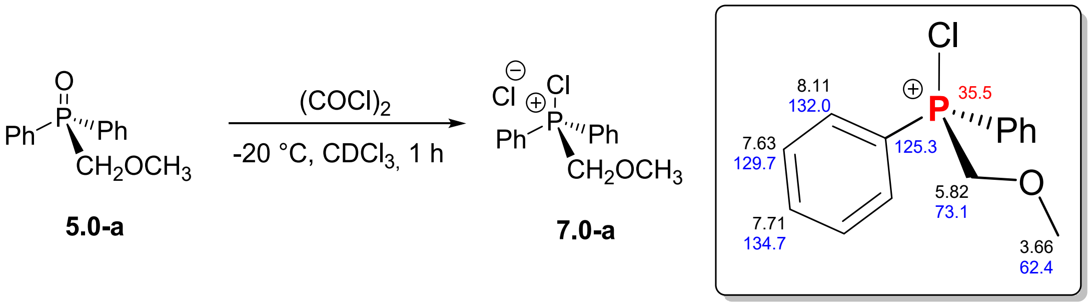

This was prepared quantitatively following general procedure **A** at -20 °C using $CDCl_3$ as reaction solvent. ^{31}P NMR (-20 °C, 243 MHz, $CDCl_3$) δ 35.5 ppm; ^{1}H NMR (600 MHz, $CDCl_3$) δ 8.15 – 8.05 (m, 4H), 7.74 – 7.68 (m, 2H), 7.67 – 7.60 (m, 4H), 5.82 (d, J = 3.0 Hz, 2H), 3.66 (d, J = 1.9 Hz, 3H) ppm; ^{13}C NMR (151 MHz, $CDCl_3$) δ 134.7 (broad), 132.0 (broad d, J = 12.6 Hz), 129.7 (broad d, J = 16.0 Hz), 125.3 (broad d, J = 98.8 Hz), 73.1 (d, J = 68.0 Hz), 62.4 (d, J = 8.0 Hz) ppm.

8.2. Collapse of **7.0-a**

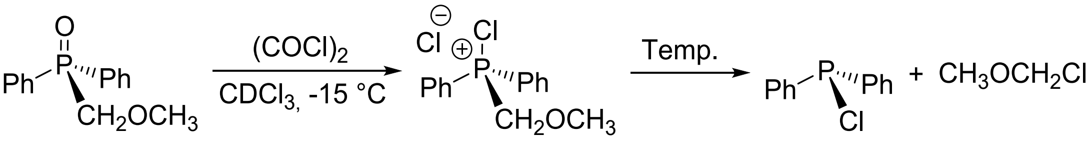

5.0-a **7.0-a** **8.0-a**

To a dry NMR tube, an appropriate amount of **5.0-a** (0.10 mmol, 1.00 equivalent) was added under an atmosphere of nitrogen and dissolved in $CDCl_3$ (1.00 mL) to give a 0.1 M solution. This was cooled to -15 °C and oxalyl chloride (0.10 mmol, 1.00 equivalent) was added whereupon the NMR tube was shaken briefly to ensure homogeneity. The reaction was monitored by ^{31}P NMR at the applicable temperature whereby the decrease in intensity of the ^{31}P NMR signal of CPS **7.0-a** served as tool to assess reaction progress, as exemplified in Figure S19. The rate constants k_1 at each temperature were determined by standard regression analysis for a first order process.

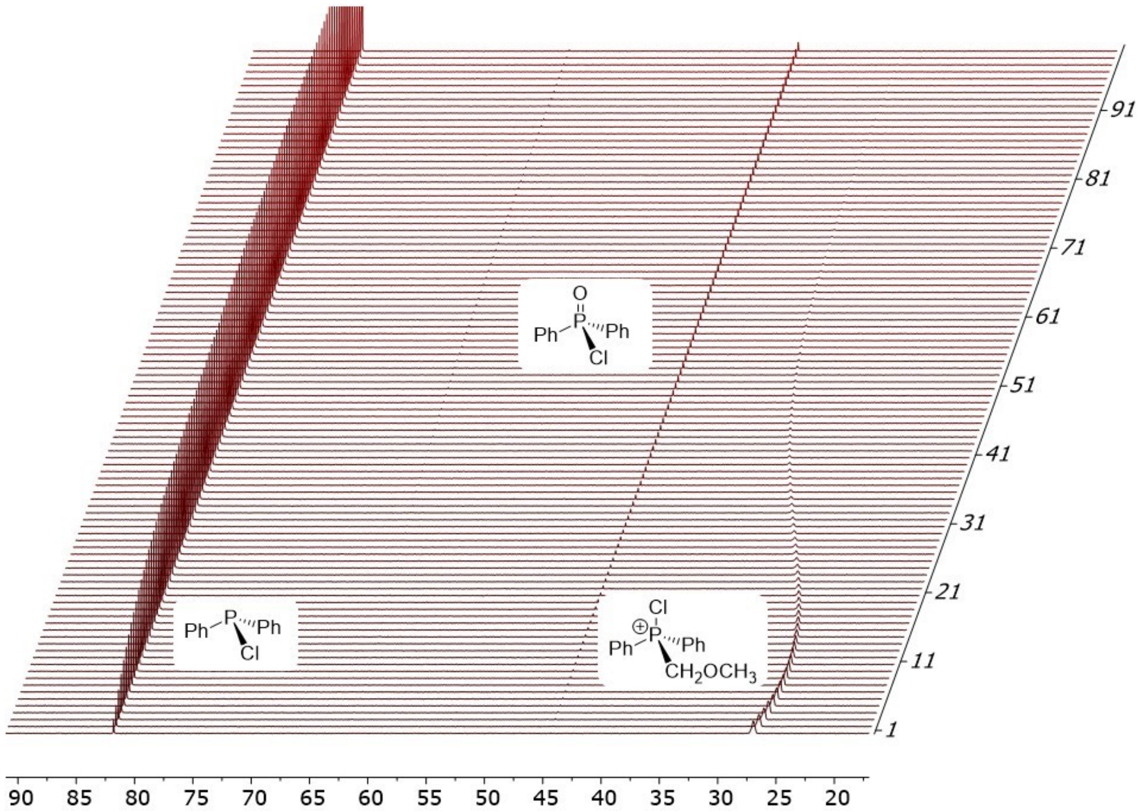

Figure S18. Example of stacked ^{31}P NMR spectra showing the progress of nucleophilic collapse or **7.0-a** as explained in 8.2.

8.3. *P*-chlorodiphenylphosphine **8.0-a** from **5.0-a** via **7.0-a**

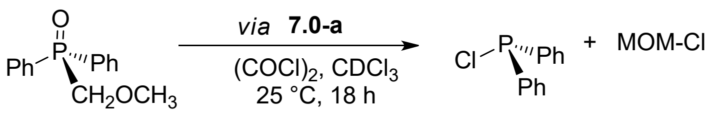

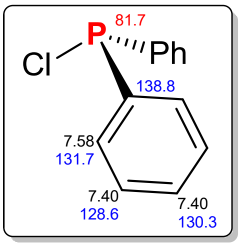

A 25 mL Schlenk flask was charged with **5.0-a** (145 mg, 0.59 mmol, 1.00 equivalent) and the material was dried under vacuum and $CDCl_3$ (2.90 mL) was added to give a 0.2 M solution of **5.0-a**. To this, oxalyl chloride (52 µL, 0.61 mmol, 1.03 equivalents) was added using a nitrogen-flushed syringe and effervescence was immediately observed. The resulting reaction mixture was stirred at ambient temperature for 18 hours. After this time, a sample (0.70 mL) was removed and submitted for NMR analysis, which indicated the presence of **8.0-a**, **7.0-a** and **5.0-a** in a 95:4:1 ratio.

Assigned to MOM-Cl: ^{1}H NMR (400 MHz, $CDCl_3$) δ 5.45 (s, 2H), 3.50 (s, 3H) ppm; ^{13}C NMR (101 MHz, $CDCl_3$) δ 84.6, 57.6 ppm.

Assigned to **8.0-a**: ^{31}P NMR (162 MHz, $CDCl_3$) δ 81.7 ppm; ^{1}H NMR (400 MHz, $CDCl_3$) δ 7.63 – 7.54 (m, 2H), 7.44 – 7.36 (m, 3H) ppm; ^{13}C NMR (101 MHz, $CDCl_3$) δ 138.8 (d, *J* = 32.5 Hz), 131.7 (d, *J* = 24.6 Hz), 130.3, 128.6 (d, *J* = 6.9 Hz) ppm.

8.4. *P*-chloro(methoxymethyl)(methyl)phenylphosphonium chloride **7.1-a** from phosphine oxide **5.1-a**

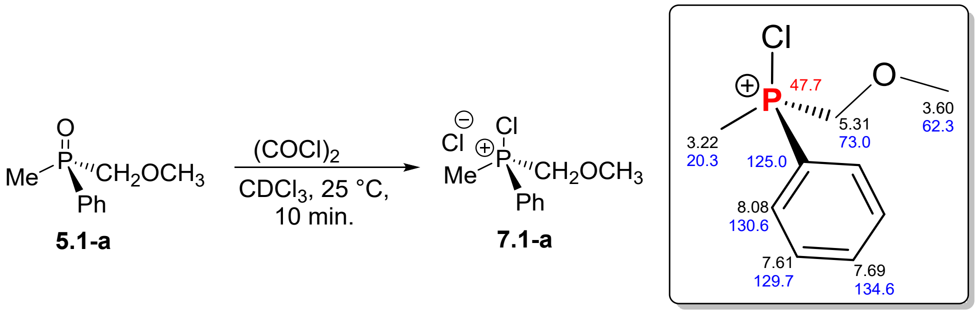

Prepared quantitatively following general procedure **A** in $CDCl_3$ at 25 °C. ^{31}P NMR (162 MHz, $CDCl_3$) δ 47.7 ppm; ^{1}H NMR (400 MHz, $CDCl_3$) δ 8.15 – 8.07 (m, 2H), 7.74 – 7.68 (m, 1H), 7.63 – 7.59 (m, 2H), 5.31 (d, *J* = 2.0 Hz, 2H), 3.60 (d, *J* = 1.7 Hz, 3H), 3.22 (d, *J* = 14.0 Hz, 3H) ppm; ^{13}C NMR (101 MHz, $CDCl_3$) δ 134.6 (d, *J* = 3.0 Hz), 130.6 (d, *J* = 9.0 Hz), 129.7 (d, *J* = 13.0 Hz), 125.0 (d, *J* = 90.0 Hz), 73.0 (d, *J* = 69.0 Hz), 62.3 (d, *J* = 10.0 Hz), 20.3 (d, *J* = 57.0 Hz) ppm.

8.5. *P*-chloro(methyl)phenylphosphine[13] **8.1-a** from **5.1-a** via **7.1-a**

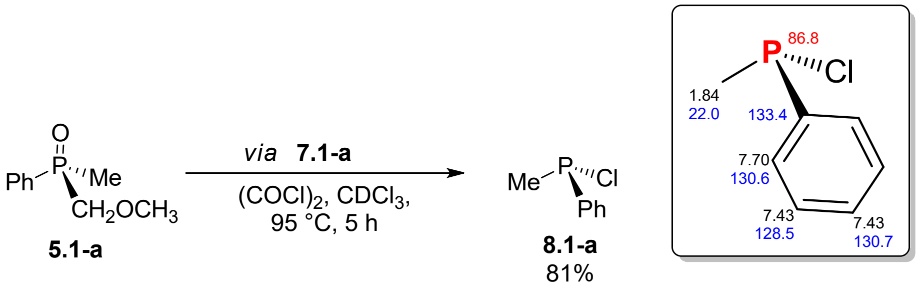

Prepared following general procedure **A** using **5.1-a** (42 mg, 0.23 mmol) and oxalyl chloride (26 µL, 0.30 mmol, 1.3 equivalents) in $CDCl_3$ (0.70 mL) at 25 °C in a sealed tube. The reaction mixture was then heated at 95 °C for 5 hours after which time ^{31}P NMR analysis of the reaction mixture indicated **8.1-a**, **7.1-a, 9.1-a,** in 81:5:14 ratio.

Assigned to **8.1a**: ^{31}P NMR (162 MHz, $CDCl_3$) δ 86.8 ppm; ^{1}H NMR (400 MHz, $CDCl_3$) δ 7.72 – 7.68 (m, 2H), 7.47 – 7.41 (m, 3H), 1.84 (d, *J* = 9.0 Hz, 3H) ppm; ^{13}C NMR (101 MHz, $CDCl_3$) δ 133.4 (d, *J* = 37.0 Hz), 130.7 (d, *J* = 3.0 Hz), 130.6 (d, *J* = 19.0 Hz), 128.5 (d, *J* = 5.0 Hz), 22.0 (d, *J* = 29.0 Hz) ppm.

8.6. Methyl(phenyl)phosphinic chloride[14] **9.1-a** from **5.1-a**

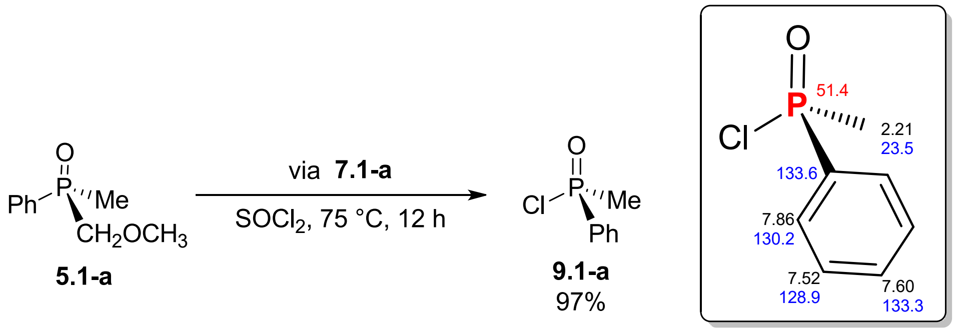

Prepared following general procedure **A** using **5.1-a** (92 mg, 0.5 mmol) and thionyl chloride (0.36 mL, 5 mmol, 10 equivalents) at 25 °C in a sealed tube. The reaction mixture was then heated at 75 °C for 12 hours after which time ^{31}P NMR analysis indicated the presence of **7.1-a** (3%) and **9.1-a** (97%) which was characterised without further purification: ^{31}P NMR (162 MHz, $CDCl_3$) δ 51.4 ppm. ^{1}H NMR (400 MHz, $CDCl_3$) δ 7.87 (dd, J = 15.0, 7.0 Hz, 2H), 7.60 (t, J = 7.0 Hz, 1H), 7.55 – 7.50 (m, 3H), 2.21 (d, J = 14.0 Hz, 3H) ppm; ^{13}C NMR (101 MHz, $CDCl_3$) δ 133.6 (d, J = 117 Hz), 133.3 (d, J = 3.1 Hz), 130.2 (d, J = 12.0 Hz), 128.9 (d, J = 14.4 Hz), 23.5 (d, J = 84.6 Hz).

8.7. *P*-Chloro(methoxymethyl)(ethyl)phenylphosphonium chloride **7.1-b** from **5.1-b**

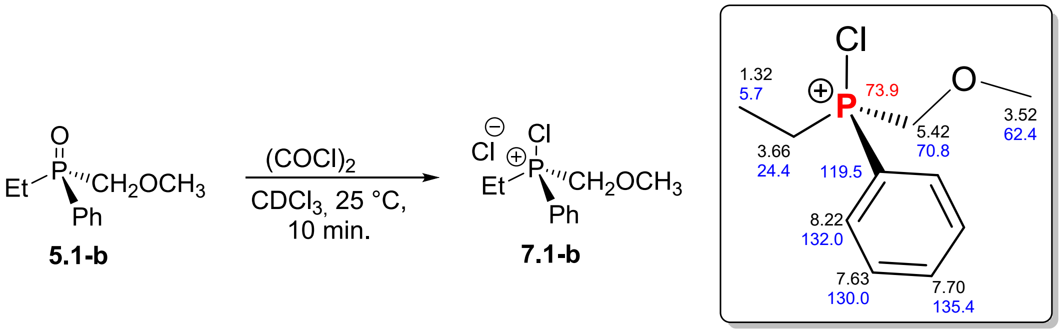

Prepared quantitatively following general procedure **A** in $CDCl_3$. ^{31}P NMR (202 MHz, $CDCl_3$) δ 73.9 ppm; ^{1}H NMR (500 MHz, $CDCl_3$) δ 8.22 (ddd, J = 14.6, 7.5, 1.7 Hz, 2H), 7.74 – 7.68 (m, 1H), 7.66 – 7.60 (m, 2H), 5.42 (d, J = 2.2 Hz, 2H), 3.66 (apparent dt, J = 15.6, 7.7 Hz, 2H), 3.52 (s, 3H), 1.32 (dt, J = 24.4, 7.4 Hz, 3H) ppm; ^{13}C NMR (126 MHz, $CDCl_3$) δ 135.4 (d, J = 3.4 Hz), 132.0 (d, J = 12.2 Hz), 130.0 (d, J = 14.3 Hz), 119.5 (d, J = 82.7 Hz), 70.8 (d, J = 61.3 Hz), 62.4 (d, J = 10.4 Hz), 24.4 (d, J = 45.3 Hz), 5.7 (d, J = 7.2 Hz) ppm.

8.8. Chloro(ethyl)phenylphosphine[13] **8.1-b** from **5.1-b** via **7.1-b**

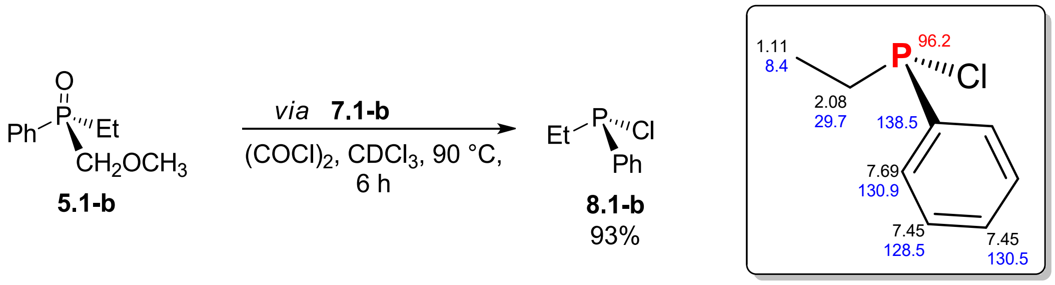

Prepared following general procedure **A** using **5.1-b** (115 mg, 0.59 mmol) and oxalyl chloride (60 µL, 0.72 mmol, 1.20 equivalents) in $CDCl_3$ (0.70 mL) at 25 °C in a sealed tube. The reaction mixture was then heated at 90 °C for 6 hours after which time ^{31}P NMR analysis of the reaction mixture indicated **8.1-b**,[8] **7.1-b** and **9.1-b** in a 93:5:2 ratio. ^{31}P NMR (162 MHz, $CDCl_3$) δ 96.2 ppm; ^{1}H NMR (400 MHz, $CDCl_3$): δ 7.77 – 7.62 (m, 2H), 7.48 – 7.40 (m, 3H), 2.13 – 2.02 (m, 2H), 1.11 (dt, J = 16.5, 7.6 Hz, 3H) ppm; ^{13}C NMR (101 MHz, $CDCl_3$): δ 138.5 (d, J = 34.4 Hz), 130.9 (d, J = 24.9 Hz), 130.5, 128.5 (d, J = 7.4 Hz), 29.7 (d, J = 26.9 Hz), 8.4 (d, J = 14.7 Hz) ppm.

8.9. Thermal collapse of **7.1-b** under various conditions

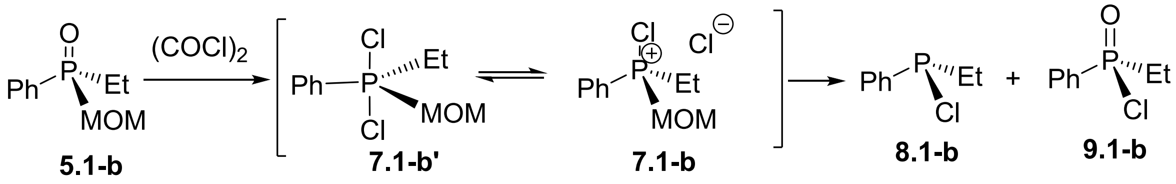

8.9.1. in $CDCl_3$

7.1-b was prepared quantitatively following general procedure A using **5.1-b** (40 mg, 0.2 mmol) and oxalyl chloride (25 µL, 0.30 mmol, 1.5 equivalents) in $CDCl_3$ (0.70 mL) at 25 °C in a sealed tube. The reaction mixture was then heated at 40 °C for 16 hours after which time ^{31}P NMR analysis of the reaction mixture indicated (121 MHz, $CDCl_3$) δ 96.4 (**8.1-b**, 6%), 70.2 (**7.1-b**, 47%), 58.7 (**9.1-b**, 26%).

8.9.2. in C_6D_6

Following general procedure **A**, **5.1-b** (28 mg, 0.14 mmol) was treated with oxalyl chloride (12 µL, 0.14 mmol) in C_6D_6 (0.70 mL). The reaction mixture was then heated at 60 °C for 96 hours after which time ^{31}P NMR (162 MHz, C_6D_6) indicated: δ 96.2 (**8.1-b**, 61%), 55.2 (**9.1-b**, 6%), 34.4 (unknown, 29%), -18.8 (**7.1-b’**, 5%) ppm.

8.9.3. in toluene

Following general procedure **A**, **5.1-b** (59 mg, 0.30 mmol) and oxalyl chloride (31 µL, 0.36 mmol, 1.20 equivalents) were combined in toluene (1.50 mL). The reaction mixture was then heated at 75 °C for 24 h after which time the ^{31}P NMR (162 MHz, C_6D_6) indicated δ 95.9 (**8.1-b**, 79%), 54.8 (**9.1-b**, 12%), -17.1 (**7.1-b'**, 9%) ppm.

8.10. Ethylphenylphosphinic chloride[13] **9.1-b** from **5.1-b**

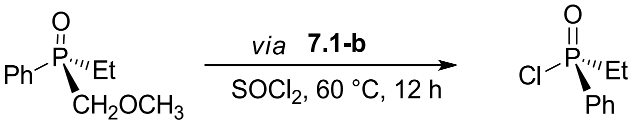

5.1-b

9.1-b
92%

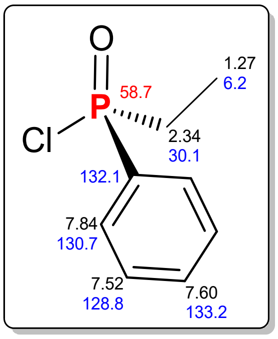

Prepared following General procedure A using **5.1-b** (98 mg, 0.50 mmol) and thionyl chloride (0.36 mL, 5 mmol, 10 equivalents) in a sealed tube. The reaction mixture was then heated at 75 °C for 12 hours after which time ^{31}P NMR analysis indicated the presence of **7.1-b (**8%)and **9.1-b** (92%): ^{31}P NMR (121 MHz, $CDCl_3$) δ 58.7; ^{1}H NMR (400 MHz, $CDCl_3$) δ 7.84 (dd, *J* = 9.0, 8.0 Hz, 2H), 7.60 (t, *J* = 8.0 Hz, 1H), 7.55-7.51 (m, 2H), 2.34 (m, 2H), 1.27 (dt, J = 22.4, 7.6 Hz, 3H) ppm; ^{13}C NMR (101 MHz, $CDCl_3$) δ 132.1 (d, *J* = 117 Hz), 133.2 (d, *J* = 3.1 Hz), 130.7 (d, *J* = 12.0 Hz), 128.8 (d, *J* = 14.4 Hz), 30.1 (d, *J* = 84.6 Hz), 6.2 (d, *J* =6 Hz, 3H).

9. Phosphines 1: Synthesis and Characterization Data

9.1. Ethyldiphenylphosphine[14] **1.1-b** from **5.0-a**

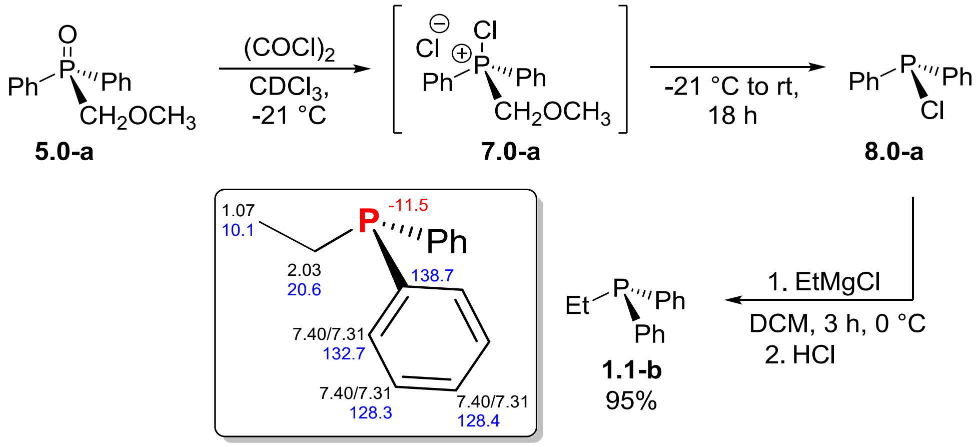

The chloride **8.0-a** was prepared from **5.0a** (148 mg, 0.60 mmol) and the volatile MOM-Cl was removed *in vacuo* to yield an oily residue, which was dissolved in DCM (2.30 mL) and cooled to 0 °C. Ethylmagnesium chloride (2.7 M in THF, 0.26 mL, 0.70 mmol, 1.16 equivalents) was added dropwise and the reaction mixture was stirred at 0 °C for 3 hours prior to the addition of a 2.0 M solution of HCl in diethyl ether (0.35 mL, 0.70 mmol, 1.16 equivalents). After 10 min the reaction solvent was removed *in vacuo* at ambient temperature to yield an oily residue. ^{31}P NMR analysis indicated the presence of **1.1-b**, **3.1-c** and **5.0-a** in a 95:1:4 ratio. To the combined reaction mixture, under an atmosphere of nitrogen, DCM (10.00 mL) was added, the organic phase was washed with deaerated water (2 x 2 mL) and concentrated *in vacuo* to yield an oily residue. The crude **1.1-b** (122 mg) was characterized without further purification: ^{31}P NMR (121 MHz, $CDCl_3$) δ -11.5 ppm; ^{1}H NMR (400 MHz $CDCl_3$) δ 7.44 – 7.37 (m, 4H), 7.35 – 7.28 (m, 6H), 2.03 (q, J = 7.6 Hz, 2H), 1.07 (dt, J = 17.0, 7.6 Hz, 3H) ppm; ^{13}C NMR (101 MHz, $CDCl_3$) δ 138.7 (d, J = 12.9 Hz), 132.7 (d, J = 18.3 Hz), 128.4, 128.3 (d, J = 6.5 Hz), 20.6 (d, J = 10.2 Hz), 10.1 (d, J = 16.4 Hz) ppm.

9.2. 4-Chlorophenyl(diphenyl)phosphine[15] **1.0-b** from **5.0-a**

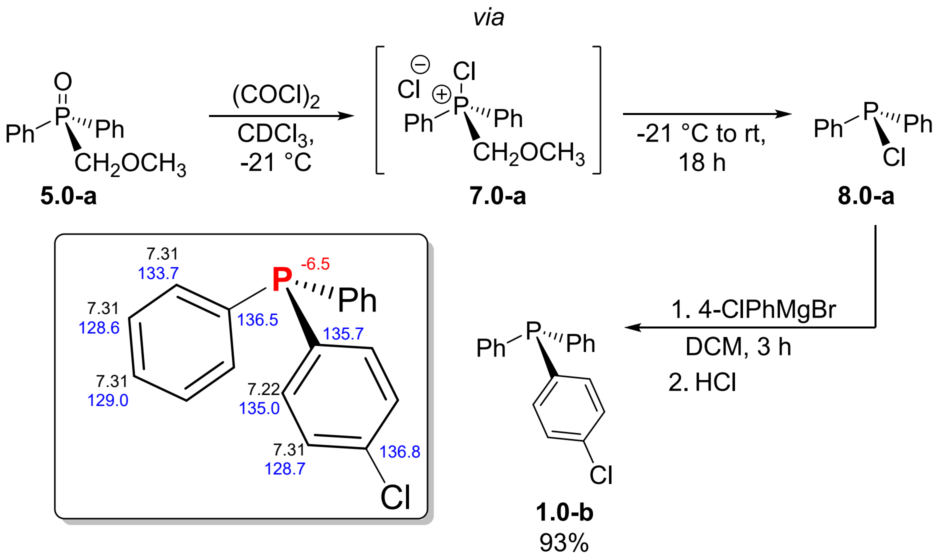

The chloride **8.0-a** was prepared from **5.0a** (159 mg, 0.65 mmol) and the volatile MOM-Cl was removed *in vacuo* to yield an oily residue, which was dissolved in DCM (3.2 mL) and cooled to 0 °C. Then, 4-chlorophenylmagnesium bromide (1.0 M in Et_2O, 0.98 mL, 0.98 mmol, 1.50 equivalents) was added dropwise and the reaction mixture was stirred at 0 °C for 3 hours prior to the addition of a 2.0 M solution of HCl in diethyl ether (0.5 mL, 1.0 mmol, 1.50 equivalents). After 10 min the reaction solvent was removed *in vacuo* at ambient temperature to yield an oily residue. ^{31}P NMR analysis indicated the presence of **1.1-c** (93%). To the combined reaction mixture, under an atmosphere of nitrogen, DCM (15.0 mL) was added, the organic phase was washed with deaerated water (2 x 2.5 mL) and concentrated *in vacuo* to yield an oily residue. The phosphine **1.0-b** (182 mg) was characterized without further purification: ^{31}P NMR (121 MHz, $CDCl_3$) δ -6.5 ppm; ^{1}H NMR (400 MHz $CDCl_3$) δ 7.37 – 7.25 (m, 12H), 7.25 – 7.19 (m, 2H) ppm; ^{13}C NMR (101 MHz, $CDCl_3$) δ 136.8 (d, *J* = 2 Hz), 136.5 (d, *J* = 9.9 Hz), 135.7 (d, *J* = 12.1 Hz), 135.0 (d, *J* = 19.9 Hz), 133.7 (d, *J* = 19.6 Hz), 129.0, 128.7 (d, *J* = 7.1 Hz), 128.6 (d, *J* = 7.1 Hz) ppm.

9.3. Methyl(diphenyl)phosphine[14] **1.1-a** from **5.1-a**

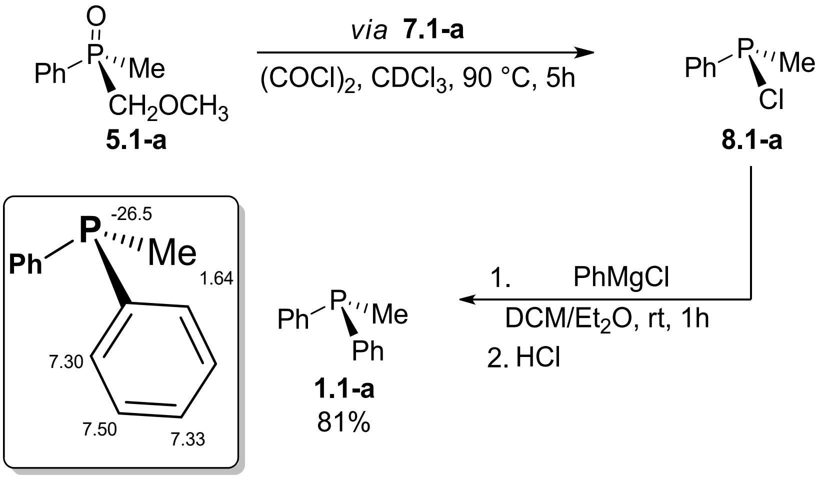

The chloride **8.1-a** was prepared from **5.1-a** (46 mg, 0.25 mmol) and oxalyl chloride (26 µL, 0.30 mmol, 1.2 equivalents) in $CDCl_3$ (0.70 mL, see 8.5) and the volatile MOM-Cl was removed *in vacuo* at 0 °C to yield an oily residue which was dissolved in a 1:1 v/v mixture of DCM and ether (2.00 mL). The solution was cooled to 0 °C and, under vigorous agitation, phenylmagnesium chloride (2.0 M in THF, 0.25 mL, 0.5 mmol, 2.00 equivalents) was added. The reaction mixture was stirred at room temperature for one hour prior to the addition of a 4 M solution of HCl in deaerated water (0.125 mL, 0.5 mmol, 2.0 equivalents). The organic layer was washed twice with deaerated water (2.0 mL) and concentrated *in vacuo* to yield an oily residue; ^{31}P NMR analysis indicated the presence of **1.1-a** (81%) and **3.1-b (**19 %). The crude mixture was purified by flash chromatography (silica; pentane) to give **1.1-a** (32 mg, 64%) as an oil: ^{31}P NMR (162 MHz, $CDCl_3$) δ, -26.5 ppm; ^{1}H NMR (400 MHz, $CDCl_3$) δ 7.50 (m, 4H), 7.33 (m, 2H), 7.30 (m, 4H), 1.64 (d, *J* = 3 Hz, 3H) ppm.

9.4. Ethyl(phenyl)-o-tolylphosphine[16] **1.1-c** from **5.1-b**

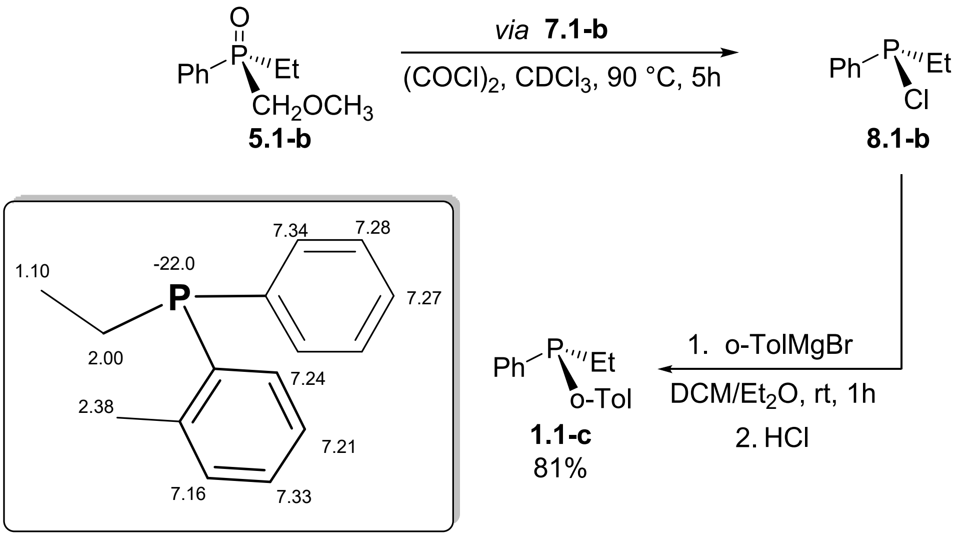

The chloride **8.1-b** was prepared from **5.1-b** (50 mg, 0.25 mmol) and oxalyl chloride (26 µL, 0.30 mmol, 1.2 equivalents) in $CDCl_3$ (0.70 mL, see 8.5) and the volatile MOM-Cl was removed *in vacuo* at 0 °C to yield an oily residue which was dissolved in a 1:1 v/v mixture of DCM and ether (2.00 mL). The solution was cooled to 0 °C and, under vigorous agitation, o-tolylmagnesium bromide (1.0 M in THF, 0.5 mL, 0.5 mmol, 2.00 equivalents) was added. The reaction mixture was stirred at room temperature for one hour prior to the addition of a 4 M solution of HCl in deaerated water (0.125 mL, 0.5 mmol, 2.0 equivalents). The organic layer was washed twice with deaerated water (2.0 mL) and concentrated *in vacuo* to yield an oily residue; ^{31}P NMR analysis indicated the presence of **1.1-c** (81%) and unknown species (18 %). The crude mixture was purified by flash chromatography (silica; pentane) to give **1.1-c** (41 mg, 72%) as an oil: ^{31}P NMR (162 MHz, $CDCl_3$) δ, -22 ppm; ^{1}H NMR (400 MHz, $CDCl_3$) δ 7.34-7.32 (m, 3H), 7.29-7.27 (m, 3H), 7.25-7.24 (m, 1H), 7.21-7.15 (m, 2H), 2.38 (s, 3H), 2.1-2.0 (m, 2H), 1.10 (dt, J = 17.0 Hz, 7.0 Hz, 3H) ppm.

9.5. Methoxymethyldiphenylphosphine[15] **10.0-a** from **7.0-a**

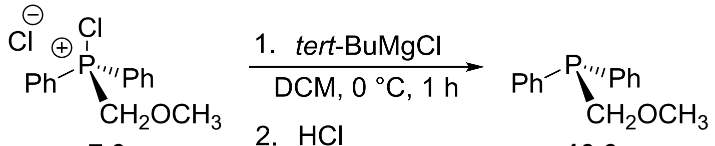

7.0-a **10.0-a** 62%

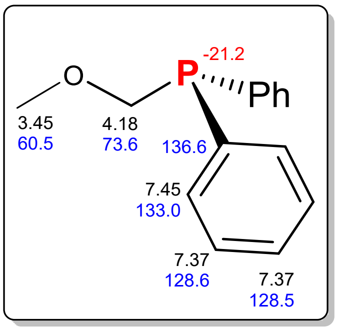

Using a 0.20 M DCM solution of **7.0-a** (5.00 mL, 1.00 mmol) and *tert*-butylmagnesium chloride (1.7 M solution in THF, 1.17 mL, 2.00 mmol) according to general procedure A at 0 °C. ^{31}P NMR (121 MHz, $CDCl_3$) analysis revealed the presence of **5.0-a**, two unknown species and **10.0-a** in a 26:9:3:62 ratio. Phosphine **10.0-a** was characterized without further purification: ^{31}P NMR (202 MHz, CD_2Cl_2) δ -21.2 ppm; ^{1}H NMR (500 MHz, CD_2Cl_2) δ 7.50 – 7.41 (m, 4H), 7.39 – 7.34 (m, 6H), 4.18 (d, J = 5.2 Hz, 2H), 3.45 (s, 3H) ppm; ^{13}C NMR (126 MHz, CD_2Cl_2) δ 136.6 (d, J = 11.3 Hz), 133.0 (d, J = 17.8 Hz), 128.6 (d, J = 11.8 Hz), 128.5 (d, J = 6.6 Hz), 73.6 (d, J = 6.1 Hz), 60.5 (d, J = 8.5 Hz) ppm.

10. **Computational Data**

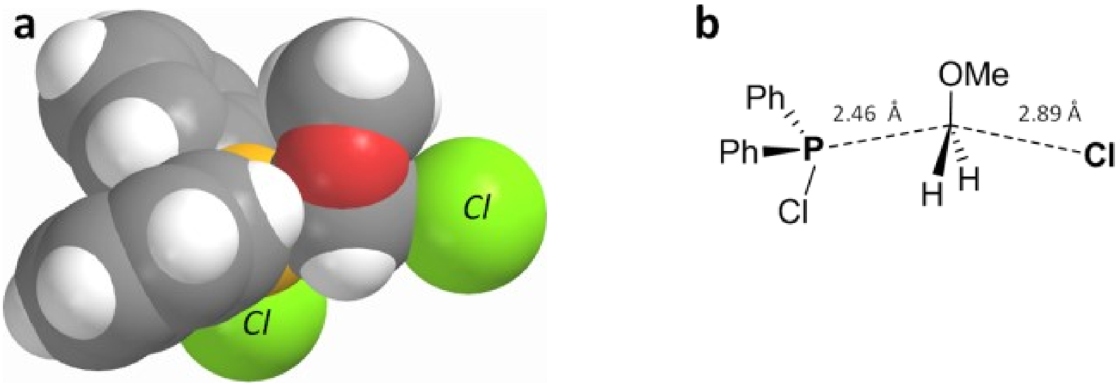

The DFT energy calculations have been done at the B3LYP/6-31G* level (SPARTAN10 suite of programs) using default convergence criterion 3 x 10^{-4} hartrees/bohr.

Table S1. Total energies of ionic and molecular species (DCM, 25 °C)

Structure	Code	*E*° , Ht	*G*°, Ht	*S*°, kcal/mol	Imaginary frequencies cm^{-1}
Ion pair	**7.0**	-1879.51431	-1879.29776	122.59	none
Transition state	**7.0-TS**	-1879.48813	-1879.27420	124.76	-232

Atom coordinates:

7.0

1	P1	0.018337005	0.120929971	0.817535322	P.3	1	M0001
2	C2	1.725256250	0.168762875	0.240861422	C.ar	1	M0001
3	C3	4.320602177	0.197500766	-0.757720970	C.ar	1	M0001
4	C4	2.685532491	1.009965406	0.826331282	C.ar	1	M0001
5	C5	2.056222376	-0.660697728	-0.847320611	C.ar	1	M0001
6	C6	3.362334040	-0.638062717	-1.334074484	C.ar	1	M0001
7	C7	3.982535660	1.021286920	0.318010451	C.ar	1	M0001
8	H8	2.430733165	1.643740187	1.668751869	H	1	M0001
9	H9	1.321231643	-1.312861624	-1.321719950	H	1	M0001
10	H10	3.621004141	-1.280931618	-2.169586682	H	1	M0001
11	H11	4.727870371	1.670822975	0.765406112	H	1	M0001
12	H12	5.333481345	0.205543383	-1.149016559	H	1	M0001
13	C13	-1.075397369	1.124690033	-0.189444196	C.ar	1	M0001
14	C14	-2.751962534	2.724708617	-1.725495565	C.ar	1	M0001
15	C15	-0.543357579	2.222261264	-0.887565334	C.ar	1	M0001
16	C16	-2.448008979	0.828443956	-0.255273728	C.ar	1	M0001
17	C17	-3.277541137	1.633088189	-1.030429629	C.ar	1	M0001
18	C18	-1.389336290	3.019840020	-1.652853111	C.ar	1	M0001
19	H19	0.517257122	2.446343561	-0.842493035	H	1	M0001
20	H20	-2.848659989	-0.019737557	0.287207574	H	1	M0001
21	H21	-4.336553829	1.404666502	-1.092440177	H	1	M0001
22	H22	-0.983452501	3.867423692	-2.195287665	H	1	M0001
23	H23	-3.407437866	3.346371629	-2.327679685	H	1	M0001
24	Cl24	-0.453692470	-2.876940187	-2.446055308	Cl	1	M0001
25	Cl25	-0.014759534	0.847419298	2.726332401	Cl	1	M0001
26	C26	-0.523689792	-1.628956411	0.942407541	C.3	1	M0001
27	H27	-0.544355979	-2.036410481	-0.088290461	H	1	M0001
28	H28	0.255127501	-2.158464031	1.515117768	H	1	M0001
29	O29	-1.774771845	-1.660415089	1.581722010	O	1	M0001
30	C30	-2.279798735	-2.993652478	1.695905504	C.3	1	M0001

31	H31	-2.399196310	-3.456615990	0.708724169	H	1	M0001
32	H32	-1.611065691	-3.611826059	2.309736969	H	1	M0001
33	H33	-3.249522842	-2.911910569	2.190021840	H	1	M0001

7.0-TS

1	P1	-0.174563352	-0.509272517	0.855179957	P.3	1	M0008[3]
2	C2	1.614586484	-0.323521657	0.573002226	C.ar	1	M0008[3]
3	C3	4.358224162	-0.122751050	0.103465240	C.ar	1	M0008[3]
4	C4	2.372480053	0.654570801	1.240554730	C.ar	1	M0008[3]
5	C5	2.237140182	-1.199059750	-0.330612881	C.ar	1	M0008[3]
6	C6	3.609069465	-1.089301461	-0.566805851	C.ar	1	M0008[3]
7	C7	3.740463451	0.748548036	1.006628281	C.ar	1	M0008[3]
8	H8	1.893760942	1.330389550	1.942499889	H	1	M0008[3]
9	H9	1.657195746	-1.955878487	-0.850841358	H	1	M0008[3]
10	H10	4.090013483	-1.763546979	-1.268463976	H	1	M0008[3]
11	H11	4.325456174	1.499539862	1.528598834	H	1	M0008[3]
12	H12	5.426850345	-0.046566049	-0.073806508	H	1	M0008[3]
13	C13	-0.914922049	1.129024195	0.529035165	C.ar	1	M0008[3]
14	C14	-2.189092600	3.523482538	-0.165975897	C.ar	1	M0008[3]
15	C15	-0.328823471	1.995254889	-0.410002818	C.ar	1	M0008[3]
16	C16	-2.151539134	1.466696375	1.105909770	C.ar	1	M0008[3]
17	C17	-2.776924019	2.662802982	0.764672635	C.ar	1	M0008[3]
18	C18	-0.968862242	3.187031275	-0.751809629	C.ar	1	M0008[3]
19	H19	0.624479041	1.750792722	-0.866388184	H	1	M0008[3]
20	H20	-2.615566235	0.808950484	1.834600561	H	1	M0008[3]
21	H21	-3.724236724	2.921791893	1.227272110	H	1	M0008[3]
22	H22	-0.506407407	3.855068249	-1.472131179	H	1	M0008[3]
23	H23	-2.682627558	4.452991925	-0.432842673	H	1	M0008[3]
24	Cl24	-2.910281411	-4.908462179	0.405542050	Cl	1	M0008[3]
25	Cl25	-0.359402991	-0.721068255	2.939576923	Cl	1	M0008[3]
26	C26	-1.178516411	-2.613222904	0.074764967	C.3	1	M0008[3]
27	H27	-0.501152833	-3.182978179	0.697748752	H	1	M0008[3]
28	H28	-2.152104815	-2.341587160	0.464340746	H	1	M0008[3]
29	O29	-0.972832002	-2.705049963	-1.206104202	O	1	M0008[3]
30	C30	-2.031521013	-2.207278061	-2.064681915	C.3	1	M0008[3]
31	H31	-2.980887086	-2.641673626	-1.743738984	H	1	M0008[3]
32	H32	-2.050372430	-1.114998478	-2.019287215	H	1	M0008[3]
33	H33	-1.779083693	-2.540719008	-3.069899533	H	1	M0008[3]

11. References

1. A. C. Vetter, K. Nikitin, D. G. Gilheany, *Chem. Commun.* 2018, 54, 5843-5846.
2. F. Dornhaus, M. Bolte, H.-W. Lerner, M. Wagner, *Eur. J. Inorg. Chem.* 2006, 1777-1785.
3. P. A. Byrne, D. G. Gilheany, *J. Am. Chem. Soc.* 2012, 134, 9225-9239.
4. H.-J. Cristau, Y. Ribeill, *Synthesis*, 1988, 11, 911 – 912.
5. T.A. Albright, W. J. Freeman, E. E. Schweizer, *J. Am. Chem. Soc.* 1975, 97, 2946-2950.
6. T. Huang, T. Chen, L-B. Han, *J. Org. Chem.*, 2018, 83, 2959- 2965
7. M. Stankevic, J. Pisklak, K. Wlodarczyk, *Tetrahedron,* 2016, 72, 810-824.
8. J. Xu, P. Zhang, Y. Gao, Y. Chen, G. Tang, Y. Zhao, *J. Org. Chem.* , 2013, 78, 8176-8183.
9. J. Yang, J. Xiao, T. Chen, S.-F. Yin, L.-B. Han, *Chem. Commun.,* 2016, 52, 12233-12236.
10. P. Xing, Z.-g. Huang, Y. Jin, B. Jiang, *Synthesis*, 2013, 45, 596-600.
11. A.M. Aguiar, K. C. Hansen, J. T. Mague *J. Org. Chem.*, 1967, 32, 2383-2387
12. C. Tejo, J. Hao Pang, D. Y. Ong, M. Oi, M. Uchiyama, R. Takita, S. Chiba *Chem. Commun.*, 2018, 54,1782-1785.
13. W. Wolfsberger, *J. Organomet. Chem.*, 1986, 317, 167-173.
14. A. Haque, K.M. Alenezi, H.E. Moll, M.S. Khan, W.-Y. Wong, Molecules, 2022, 27, 4253.
15. Y. Li, S. Das, S. Zhou, K. Junge, M. Beller, *J. Am. Chem. Soc.,* 2012, 134, 9727−9732.
16. K. Nikitin, E. V. Jennings, S. S. Al Sulaimi, Y. Ortin, D. G. Gilheany, *Angew. Chem. Int. Ed.,* 2018**,** 57, 1480-1484.
